# Structural basis of the regulation by CDK11 kinase of early spliceosome activation and evidence for its proofreading by DHX15 helicase

**DOI:** 10.1038/s41467-026-75109-2

**Published:** 2026-07-03

**Authors:** Zhenwei Zhang, Vinay Kumar, Sumeru Panta, Jiayun Zhong, Olexandr Dybkov, Haohao Dong, Berthold Kastner, Henning Urlaub, Holger Stark, Reinhard Lührmann

**Affiliations:** 1 State Key Laboratory of Biotherapy and Department of Rheumatology and Immunology, West China Hospital, Sichuan University, Chengdu, China; 2 Emeritus Group Cellular Biochemistry, Max-Planck-Institute for Multidisciplinary Sciences, Göttingen, Germany; 3 Department of Structural Dynamics, Max-Planck-Institute for Multidisciplinary Sciences, Göttingen, Germany; 4 Bioanalytical Mass Spectrometry, Max-Planck-Institute for Multidisciplinary Sciences, Göttingen, Germany; 5 State Key Laboratory of Biotherapy and National Clinical Research Center for Geriatrics, West China Hospital, Sichuan University, Chengdu, China; 6 Bioanalytics Group, Institute for Clinical Chemistry, University Medical Center Göttingen, Göttingen, Germany

**Keywords:** RNA splicing, Cryoelectron microscopy

## Abstract

Formation of the activated human spliceosome (B^act^) involves major structural rearrangements, leading to the catalytically active U2/U6 RNA core. This process involves at least two intermediates, pre-B^act-1^ and pre-B^act-2^, and is regulated by CDK11-mediated phosphorylation of the U2 snRNP protein SF3B1. However, the mechanisms of this essential step are poorly understood. Here we present the cryo-EM structure of a spliceosome stalled – by the CDK11 inhibitor OTS964 – in a previously undescribed early-activated state, termed pre-B^act-OTS^, shortly after dissociation of U4 snRNP. In pre-B^act-OTS^, the U2-SF3B6 protein is retained in a C-terminal region of the super-helical U2-SF3B1 HEAT domain (SF3B1^HEAT^) that clamps the U2/branch-site helix. In contrast, in pre-B^act-1^, SF3B6 is repositioned to SF3B1’s N-terminal HEAT repeats, thereby preventing a steric clash of SF3B6 with PRP8 during the pre-B^act-OTS^-to-pre-B^act-1^ transition. We infer that the CDK11-mediated phosphorylation of SF3B1 drives the relocation of SF3B6, gating progression to B^act^ formation. In pre-B^act-OTS^, we also located the RNA helicase DHX15 at the N-terminal region of SF3B1^HEAT^, assisted by the SR140/SPF45/CHERP/SUGP1 protein complex. These results suggest the involvement of DHX15 in kinase-mediated proofreading of the early-activated spliceosome, by competing with CDK11’s phosphorylation of SF3B1, and thus with relocation of SF3B6 at SF3B1^HEAT^.

## Abstract (web summary)

Human spliceosome activation requires phosphorylation of U2 protein SF3B1. Here the authors determine the cryo-EM structure of an early activated complex, stalled by a CDK11 inhibitor, revealing a kinase-driven remodeling step in which the activation is gated and appears subjected to proofreading.

## Introduction

For each round of pre-mRNA splicing, a spliceosome is assembled, catalytically activated, and, after splicing catalysis, dismantled^1^. The stepwise recruitment of the U1 and U2 small nuclear RNPs (snRNPs) and the U4/U6.U5 tri-snRNP, plus numerous proteins, to the pre-mRNA, generates the spliceosomal B complex^2^. The latter is transformed into an activated spliceosome, termed B^act^, by dramatic changes in its protein composition and structure that lead to the formation of the catalytic U2/U6 RNA network.

Spliceosome activation is initiated by the RNA helicase BRR2, which unwinds the U4/U6 duplex in the B complex, leading to the expulsion of the U4 snRNP. That dissociation allows U6 snRNA to restructure (forming an internal stem-loop, ISL^3^) and to engage in new base-pair formation with U2, generating U2/U6 helices Ia and Ib^4^. Tertiary interactions between a bulged-out nucleotide of the U6 ISL (U74 in humans) and the U6 catalytic triad AGC nucleotides, which are part of U2/U6 helix Ib, as well as the U6 GA dinucleotide directly downstream of its ACAGA box, lead to formation of the catalytic U6 snRNA triplex and enables U6 to position two Mg^2+^ ions that participate directly in splicing catalysis^5–7^. During activation about 25 proteins, including the U4/U6 and B-specific proteins, leave the nascent B^act^ complex, while 25 new proteins are recruited, including the retention and splicing (RES) complex, the PRP19 complex (nineteen complex, hNTC), and NTR proteins^8^. Recently, two studies, using small-molecule splicing inhibitors, revealed cryo-EM structures of three human activated-spliceosome precursors, termed pre-B^act-1^ and pre-B^act-2^^9^, or just pre-B^act^^10^, the latter representing an intermediate assembly stage that arises after pre-B^act-2^ but before the formation of mature B^act^ complexes. These structures not only provided insights into the stepwise folding pathway of the U2/U6 catalytic RNA core and the sequential protein exchanges that occur during activation^9,10^; they also revealed that U2 snRNP, along with other protein and RNA structural elements, undergoes large-scale, stepwise repositioning during the transitions from B to pre-B^act^ and from pre-B^act^ to B^act^, although the driving forces underlying these remodeling events remain poorly understood.

During the B-to-B^act^ transition, the U2 protein SF3B1 is heavily phosphorylated, raising the possibility that such post-translational modifications could play a part in the activation process^11,12^. SF3B1 contains an unstructured N-terminal region and 20 C-terminal HEAT repeats, the latter forming a C-shaped scaffold that clamps the U2/branch-site (BS) helix during spliceosome assembly and activation^13,14^. Phosphorylation occurs primarily at numerous TP and SP dipeptide repeats located within the N-terminal region of SF3B1^15,16^. Recently it was demonstrated that the cyclin-dependent kinase 11 (CDK11) complex, comprising the three subunits CDK11, cyclinL1 and the protein SAP30BP^17^, is the major kinase that phosphorylates SF3B1, and that the phosphorylation can be blocked by OTS964, a highly selective inhibitor of CDK11^17–19^. Moreover, it was observed that OTS964 inhibited spliceosome activation, which further showed that phosphorylation of SF3B1 by CDK11 kinase is an essential regulatory event during the transformation of the B into the mature B^act^ complex^18^. However, it is still unclear which of the remodeling steps that occur during spliceosome activation (such as the BRR2-mediated U4 RNP displacement or formation of one of the pre-B^act^ complexes) require phosphorylation of SF3B1.

Several RNA helicases that drive the rearrangements required during spliceosome assembly have also been proposed as establishing fidelity in splicing by a kinetic proofreading mechanism^20,21^ in which the DEAD/H-box ATPase promotes an optimum substrate by acting after a productive step and by rejecting a suboptimal substrate (or incorrectly structured complex) and consequently driving the substrate into an “off-pathway” state^21–23^. Such aberrant, off-pathway spliceosomes may then be dismantled by the RNA helicase DHX15 (also termed PRP43). DHX15 is well known for its role in dismantling the terminal ILS spliceosome, in cooperation with its ATPase-activating G-patch protein TFIP11^24–26^. Recent studies, however, have suggested that DHX15 may have a more general function in the quality control of pre-mRNA splicing in human cells^27–30^. Moreover, it has been shown that the G-patch protein SUGP1 triggers DHX15’s ATPase activity, to disrupt aberrant, pre-catalytic spliceosome complexes^27,28,31^. It is currently not well understood at which spliceosome assembly stage DHX15 and SUGP1 cause incorrectly formed spliceosomes to dissociate. Whether such putative quality-control mechanisms involve other enzymes, in addition to helicases, is also unknown.

Here, we present the cryo-EM structure of a human spliceosome stalled by the CDK11 inhibitor OTS964 in a novel intermediate assembly state that we term pre-B^act-OTS^ and which arises between the BRR2-mediated dissociation of U4 snRNP and the formation of the pre-B^act-1^ complex. Our structure indicates how the large-scale movement of U2 snRNP is guided, and it suggests a mechanism for the proofreading of early-activated spliceosomes by DHX15: the CDK11-mediated phosphorylation of SF3B1 drives the relocation of the protein SF3B6, gating progression to pre-B^act-1^. In correctly assembled complexes, CDK11 efficiently phosphorylates SF3B1, triggering rapid SF3B6 relocation to HRs 1–2 and allowing further progression of spliceosome activation. Aberrant spliceosomes would impair CDK11 recruitment (and thus SF3B1 phosphorylation), in turn stalling the repositioning of SF3B6 and thus enabling DHX15 to dock to SF3B1^HEAT^, inducing spliceosome disassembly.

## Results

### The cryo-EM structure of the OTS964-stalled spliceosome reveals an early-activated pre-B^act^ complex

**Fig. 1:**
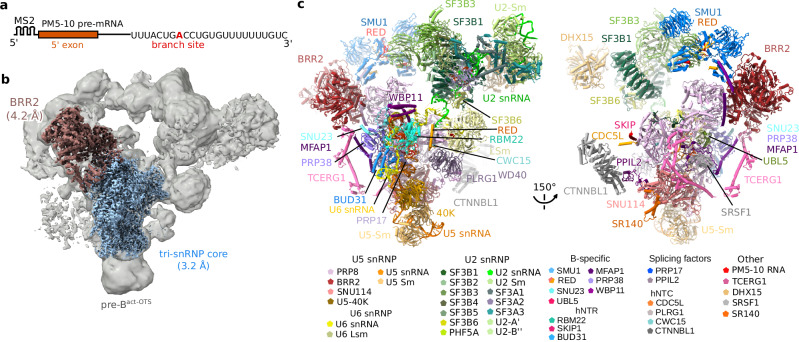
3D structure of human PM5-10 pre-B^act-OTS^ complex. **a**, Schematic of the PM5-10 pre-mRNA construct. The sequence at the branch site and the polypyrimidine tract is shown. The branch-site adenosine is shown in red. For affinity purification of spliceosomes, the PM5-10 pre-mRNA contains three MS2 aptamers at its 5′ end. **b**, EM densities of the pre-B^act-OTS^ complex, with the blue part showing the tri-snRNP core region (3.12 Å average resolution), the brown part showing the BRR2 helicase region (4.2 Å average resolution) and the gray part showing the U2 snRNP and other peripheral regions (15–30 Å resolution). **c**, Two different views of the molecular architecture of the pre-B^act-OTS^ complex. Summary of all modeled proteins and RNAs, color-coded according to the views above.

To elucidate the molecular architecture of an OTS964-stalled spliceosome, we used PM5-10 pre-mRNA, which lacks the second exon (Fig. 1a). It allows efficient B-to-B^act^ complex transformation in HeLa splicing extract^12^ but it stalls spliceosome formation at the activated (B^act^) stage (Supplementary Fig. 1a). In the presence of 0.2 µm OTS964; however, PM5-10 B^act^ complex formation is completely inhibited, and a significant fraction of stalled spliceosomes migrates in native gels in a manner similar to that of B/pre-B^act^ stage complexes (Supplementary Fig. 1a). The IC50 of OTS964 for CDK11 is less than 100 nM^18,19^.

We purified the OTS964-stalled complexes by affinity chromatography, using MS2 tag-containing PM5-10 pre-mRNA (Fig. 1a) and glycerol-gradient centrifugation. Consistently with previous observations^18^ and with the absence of B^act^ complexes in native gels (Supplementary Fig. 1a), OTS964 blocked the phosphorylation of SF3B1 in our gradient-purified 40S spliceosomes, as shown by immunoblots with anti-SF3B1 antibodies (Supplementary Fig. 1b). The enriched spliceosomes contained similar amounts of pre-mRNA, U2, U5 and U6 snRNAs, but reduced amounts of U4 snRNA and U4-associated proteins (Supplementary Fig. 1c and Supplementary Data 1), indicating that a significant fraction of the 40S spliceosomes had undergone BRR2-mediated unwinding of U4/U6 snRNAs. In addition to U2 and the major U5 proteins, most of the B-specific proteins (except for FBP21), the hNTC and numerous hNTR proteins (such as RBM22, BUD31, SKIP and PPIL2), as well as IBC proteins, were found to be abundant, while the RES complex proteins and the group of late B^act^ proteins (CWC22, RNF113A, CWC27, SRRM2) was essentially absent (Supplementary Data 1). Our biochemical results thus indicate that, in the presence of OTS964, a significant fraction of spliceosomes is stalled after BRR2-mediated activation but before mature B^act^ formation – that is, in a pre-B^act^-type state.

**Fig. 2:**
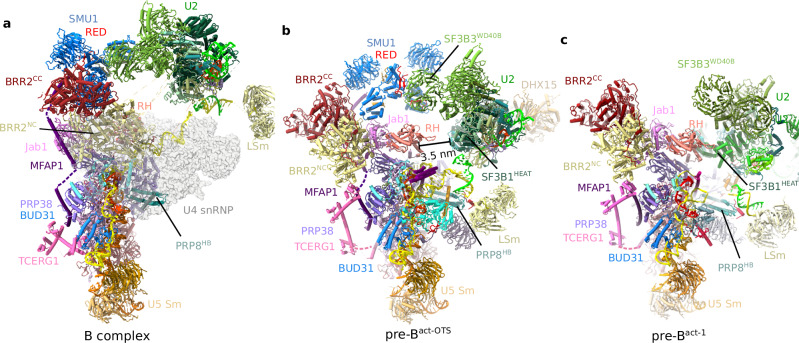
Stalling of U2 snRNP translocation at an intermediate position in PM5-10 pre-B^act-OTS.^ Structural remodeling from spliceosomal B complex (**a**, PDB: 8QO9) to pre-B^act-OTS^ (**b**) and pre-B^act-1^ complexes (**c**, PDB: 7ABG). After the departure of U4 snRNP, the U2 snRNP moves towards PRP8^RH^. However, in the pre-B^act-OTS^ complex, it is stalled at an intermediate position such that the SF3B1^HEAT^ is about 3.5 nm away from its position in pre-B^act-1^, where it interacts with PRP8^RH^.

The OTS964-stalled 40S spliceosomes were subsequently investigated by single-particle cryo-EM. 2D and 3D classifications revealed the presence of one major class of particles, whose cryo-EM structure was determined with a resolution of 3.12 Å at its RNP center (Fig. 1b and Supplementary Fig. 2); we did not observe any class averages reminiscent of B- or B^act^-stage complexes. The molecular architecture of the OTS964-stalled complex reveals that BRR2-mediated activation has occurred (consistently with its RNA composition; Supplementary Fig. 1c), and it shows similarities to, but also significant differences from, that of the previously characterized pre-B^act-1^ complex (Figs. 1 and 2). For example, BRR2 and its tightly associated PRP8^Jab1^ domain have translocated from their B complex position to their pre-B^act-1^ position, where PRP8^Jab1^ interacts with the PRP8 RNase H-like domain (PRP8^RH^) as in pre-B^act-1^ (Fig. 2 and Supplementary Fig. 3a, b). PRP8 retains its half-closed conformation as in B, pre-B^act-1^ and pre-B^act-2^, stabilized by the B-specific proteins PRP38, SNU23, MFAP1 and UBL5 (Fig. 1 and Supplementary Fig. 3c). Moreover, NTR proteins BUD31 and RBM22, as well as certain parts of SKIP and CWC15, have adopted positions as in pre-B^act-1^ (Figs. 1 and 2, see also below).

One major difference, however, between the OTS964-stalled complex and pre-B^act-1^ is the position of the U2 snRNP. In pre-B^act-1^, the 5*’* domain of U2 snRNP, containing the SF3B1-7 protein complex, plus the U2/branch site (BS) helix, has moved downwards from its B-complex position, by about 10 nm, toward the PRP8^RT/En^ domain, and its SF3B1 HEAT domain (SF3B1^HEAT^) has docked at PRP8^RH^ (Fig. 2a, c)^9^. In our new complex, the U2 5*’* domain has also moved downwards towards PRP8^RT/En^, but its movement is stalled at an earlier position, so that SF3B1^HEAT^ is still separated from PRP8^RH^ (Fig. 2b). Thus, these data indicate that OTS964 stalls the spliceosome at the stage of a previously unidentified, early-activated assembly intermediate, which we term pre-B^act-OTS964^ (pre-B^act-OTS^ for short).

### Juxtaposition of an extended U6-ILS with the U2 stem-loop1 in pre-B^act-OTS^

**Fig. 3:**
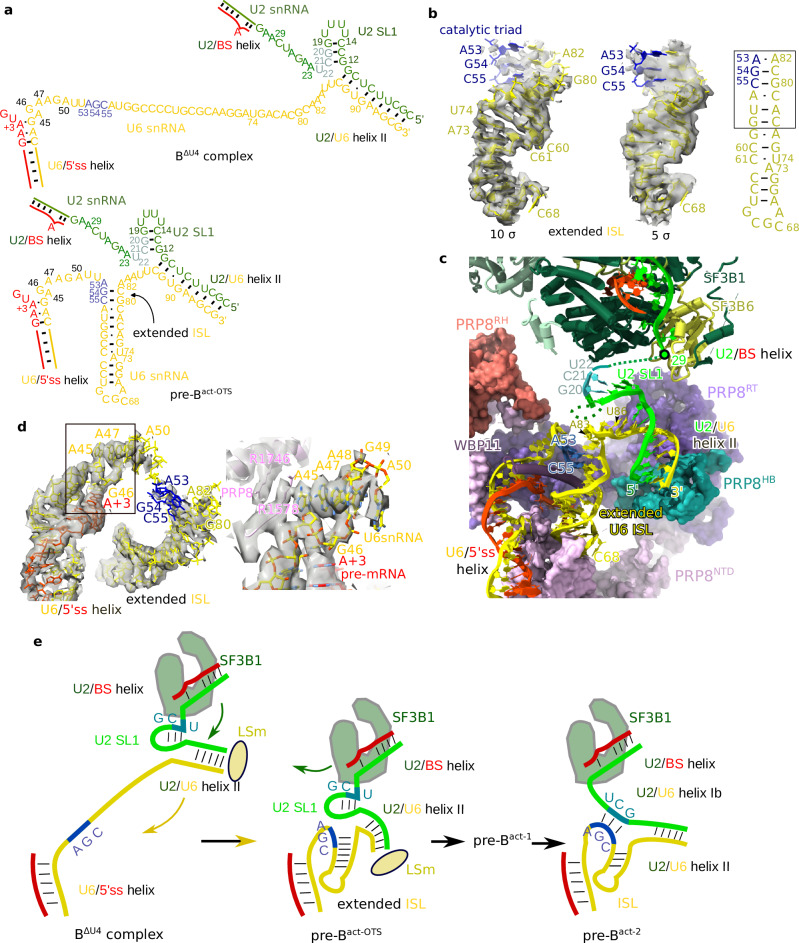
Extended U6 ISL formation and tethering of U2 snRNP to the core of pre-B^act-OTS.^ **a** Schematic representation of the RNA remodeling during the transition from B to pre-B^act-OTS^. The formation of an extended U6 ISL positions the U2 SL1 close to the end of the ISL’s stem. **b** EM density of the extended ISL, with the U6 snRNA model fitted into the density at 10σ threshold and 5σ threshold. Schematic representation of the extended ISL is shown alongside: A73 is stacked within the helix and C68 bulges out from the loop. As indicated by the boxed region, the extended ISL includes six additional base pairs at its base (compared with in pre-B^act-1^), involving, among others, the catalytic A53-G54-C55 triad and U6 nucleotides G80–A82. **c** An overview of the RNA core and the surrounding protein components. The extended ISL is docked onto PRP8^NTD^. WBP11’s long α helix is inserted into the tunnel created by the extended ISL and the extended U6/5*’*ss helix. The U2/U6 helix II is docked onto PRP8^HB^. U2 SL1 is located on top of the U2/U6 helix II, positioning U2’s nucleotides 20–22 close to the U6 catalytic triad (nts 53–55). **d** The EM density of the extended U6/5*’*ss helix, the extended U6 ISL and the linking U6 region. The boxed region is enlarged, and U6 A_45_ is flipped out from the U6/5*’*ss helix, packing against PRP8 residues R1578 and R1746, while stacking with the base of U6 A_47_; U6 G_46_ is also flipped out and stacks with A_+3_ of the pre-mRNA intron. The bases of U6’s nucleotides A47 to A50 are stacked and run perpendicular to the U6/5*’*ss helix, while U6 nts U51 and U52 are flexible, forming the connection to the extended U6 ISL. **e** Schematic depiction of the coordination of the translocation of the U2 5*’* domain (simplified as SF3B1 bound to the U2/BS helix) with the rearrangements of RNA core components during the B-to-pre-B^act-OTS^-to-pre-B^act-2^ complex transition (see text).

The principal functional hallmark of the “pre-B^act-1^ to pre-B^act-2^ to B^act^” remodeling is the stepwise formation of the U2/U6 RNA catalytic site. As the pre-B^act-OTS^ complex is stalled by OTS964 shortly after the BRR2-mediated displacement of U4 snRNP from the B complex, but before the formation of pre-B^act-1^, it was interesting to see whether U6 and U2 snRNAs were also captured in a previously unobserved state. Indeed, we found that U6 snRNA adopts an extended internal stem-loop (ISL) in pre-B^act-OTS^ that differs from its conformation in pre-B^act-1^. This extended ISL includes six additional base pairs at its base, involving the catalytic A53-G54-C55 triad and U6 nucleotides G80–A82 (Fig. 3a, b and Supplementary Fig. 4a)^9^. Within this helix, only A73 remains unpaired and stacked within the helix (Fig. 3b), which also differs from the base-pairings within the ISL in pre-B^act-1 9^. The ISL structure in our pre-B^act-OTS^ complex is similar to yeast U6 snRNA within the U6 snRNP complex, which also exhibits an extended ISL structure that includes the invariant AGC triad at the base of the ISL stem^32,33^. Thus, the extended ISL appears to be a default structure into which this part of U6 RNA folds immediately after BRR2-helicase-mediated unwinding of the U4/U6 stems I and II within the B complex. The loop of the extended U6 ISL (nts 65-69) is docked at PRP8’s N-terminal domain (PRP8^NTD^; Fig. 3c). Our cryo-EM structure further resolves the U6 nts A_45_ to U_52_ that connect the U6/5*’*ss helix (comprising U6 nts 30–44) with the extended U6 ISL (Fig. 3d). The linker region of U6 (45–52), the extended U6 ISL and the U6/5*’*ss helix are further stabilized by the N-terminal α helix of WBP11, which is inserted into a recess created by these U6 RNA structural elements (Supplementary Fig. 4b–d).

In pre-B^act-OTS^, we can also trace the U6 nts A_83_ to U_86_, which connect the U6 ISL with the U2/U6 helix II (Fig. 3a, d). While the U2/U6 helix II in the pre-B^act-1^ complex aligns parallel to the PRP8 HB domain (PRP8^HB^), it is positioned perpendicular to PRP8^HB^ in pre-B^act-OTS^ (Fig. 3c and Supplementary Fig. 4e; discussed further below). A density element with the dimensions of a short RNA helix is located directly adjacent to the upper end of the U2/U6 helix II. We identified this density element as U2 5ʹ stem-loop 1 (SL1), in which U2 nucleotides G12–C14 form intramolecular base pairs with C21–G19 (Fig. 3a, c and Supplementary Fig. 4e). Thus, in pre-B^act-OTS^ the 5*’* part of the U2 RNA has retained the rather compact configuration that it adopts in the B complex.

The above architectural features of the pre-B^act-OTS^ complex suggest that the folding of the extended U6 ISL probably drives the large-scale downward movement of the 5*’* domain of U2 snRNP, that is, upon displacement of U4 snRNA, the single-stranded U6 snRNA will spontaneously fold into the extended ISL^32,33^. As the 5*’* branch of the U6 ISL is connected to the extended U6/5*’*ss helix (which in turn is anchored at PRP8^NTD^), winding of the 3*’* branch of the U6 ISL around the 5*’* branch will pull the 5*’* domain of U2 snRNP via the U2/U6 helix II toward the PRP8 RT/HB domain; upon full formation of the extended ISL and docking of its loop at PRP8^NTD^ (Fig. 3a, c, e and Supplementary Movie 1), spliceosome assembly is transiently stalled at the pre-B^act-OTS^ state. As a result of these coordinated movements, the U2 snRNA SL1 is positioned close to the base of the U6 ISL, thus juxtaposing the U2 sequence G_20_C_21_U_22_ with the U6 catalytic triad AGC. U2 G_20_–U_22_ and the U6 AGC triad are thereby poised to form U2/U6 helix Ib during the following remodeling steps, which transform pre-B^act-OTS^ to pre-B^act-2^ (Fig. 3e).

### Coordination by SMU1/RED of the contra-rotating movements of U2 snRNP and BRR2

**Fig. 4:**
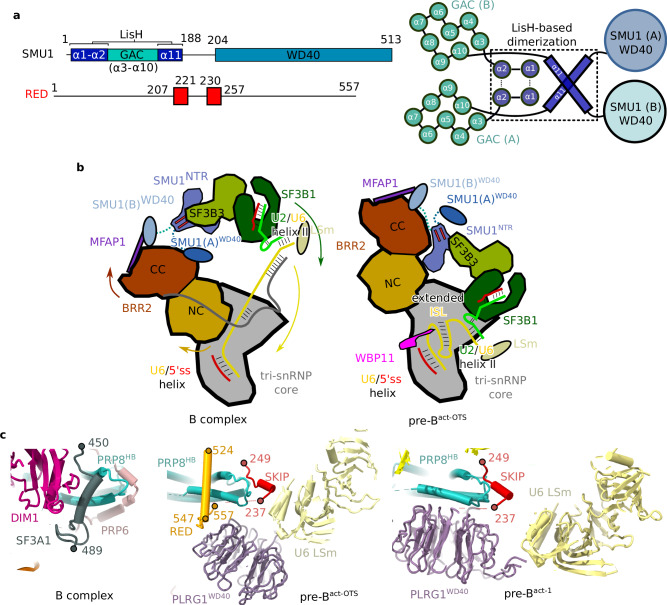
Structural organization of SMU1/RED and NTC proteins in pre-B^act-OTS^ complexes. **a** Schematic of the domain organization of SMU1 and RED, as well as schematic depiction of the SMU1 dimerization domain. LisH, Lissencephaly type 1-like homology; GAC, globular α-helical core; WD40, β-propeller-like domain. The SMU1 α helices 1, 2 and 11 together form the LisH-based dimerization domain. **b** Coordinated movements of the U2 snRNP 5*’* domain, the SMU1/RED tetrameric complex and the BRR2 helicase during the B-to-pre-B^act-OTS^ transition. For details see text and Supplementary Fig. 5b. MFAP1 and WBP11, B-specific proteins. **c** In the B complex, SF3A1 (aa 451–489) binds to the same region of PRP8^HB^ as RED (aa 524–547) in pre B^act-OTS^, suggesting a mutually exclusive interaction between SF3A1 and RED with PRP8^HB^. In pre B^act-OTS^, the binding of RED’s C-terminal α-helix to PRP8^HB^ in pre-B^act-OTS^ is mutually exclusive with that of PLRG1^WD40^ in pre-B^act-1^. This may explain why in pre-B^act-OTS^, the U6 LSm complex and PLRG1^WD40^ occupy an intermediate position at PRP8^HB^ compared with pre-B^act-1^.

SMU1 and RED proteins form a tetrameric complex^34,35^ (Fig. 4a) that bridges between BRR2 and the 5*’* domain of U2 snRNP in the B complex^35^ (Supplementary Fig. 5a, b), while the SMU1/RED complex is displaced from pre-B^act-1 9,35^. It is therefore noteworthy that in pre-B^act-OTS^ the SMU1/RED complex still connects BRR2 and the U2 5*’* domain, although the latter domains have undergone large-scale, contra-rotating translocations (Fig. 2a, b and Supplementary Fig. 5b), suggesting that SMU1/RED may support their complex remodeling events during the early stage of spliceosome activation.

Comparison of the interfaces of SMU1/RED with SF3B3 of the U2 snRNP 5*’* domain and with BRR2 in B and pre-B^act-OTS^ reveals the movements that these elements undergo during this transition (Fig. 4b and Supplementary Fig. 5b, c and Supplementary Movie 2). During the transition from B to pre-B^act-OTS^, the 5*’* domain of U2 snRNP moves downward by about 9 nm and turns clockwise by about 45° (Fig. 4b, Supplementary Fig. 5b), while BRR2 rotates clockwise by about 100° towards U2 snRNP (Supplementary Movies 2,3). While the interface between U2 snRNP’s 5*’* domain and one of SMU1/RED’s GAC (globular alpha-helical core) domains is maintained during the transition, the movement of U2 is coupled with the docking of SMU1’s second GAC domains to BRR2^CC^ (Fig. 4b, Supplementary Fig. 5c and Supplementary Movies 2,3). This leads to the formation of a new bridge between BRR2 and the U2 5*’* domain (Fig. 2b and Supplementary Fig. 5b), which stabilizes the position of the latter in the pre-B^act-OTS^ complex.

Furthermore, the N- and C-terminal regions of RED, whose functions have until now remained enigmatic, also appear to contribute to the stabilization of the pre-B^act-OTS^ state. For example, a C-terminal α helix of RED (aa 524–547), predicted by AlphaFold3 (AF3)^36^, has docked onto PRP8^HB^, fitting well into the corresponding density of our map; it is also consistent with our crosslinks (Supplementary Figs. 4e and 5 d, and Supplementary Data 2). We note that the binding of RED’s α helix to PRP8^HB^ is mutually exclusive with that of an α helix of SF3A1 in the B complex (Fig. 4c). The anchoring of SMU1/RED at the PRP8 core appears to be supported by RED’s N-terminal region, which latches onto the U2 snRNP 5*’* domain, but also interacts with PRP8^HB^, as indicated by crosslinks to the latter domain, the SF3B1 C-terminal HEAT domain and SF3B6 (Supplementary Fig. 5d). In summary, the SMU1/RED complex appears to play at least two previously unobserved roles in the early phase of spliceosome activation: it coordinates the contra-rotating movements of BRR2 and the U2 5*’* domain during the B-to-pre-B^act-OTS^ transition, and it stabilizes the U2 snRNP position in the latter state.

### Location of NTC/NTR proteins at intermediate positions in the pre-B^act-OTS^ complex

The location of the C-terminal region of RED at PRP8^HB^ in pre-B^act-OTS^ also affects the docking of several NTC proteins at PRP8. For example, in pre-B^act-OTS^, the WD40 domain of the NTC protein PLRG1 (PLRG1^WD40^) occupies an intermediate docking site at the rim of PRP8^HB^, as its position on PRP8^HB^ in pre-B^act-1^ is mutually exclusive with that of RED’s C-terminal α helix in pre-B^act-OTS^ (Fig. 2b, c, Fig. 4c and Supplementary Fig. 4e). This intermediate placement of PLRG1^WD40^ also affects the position of the adjacent LSm ring, which likewise adopts an intermediate position at the rim of PRP8^HB^ (Fig. 2b, c, Fig. 4c and Supplementary Fig. 4e). Consequently, the U2/U6 helix II, tethered to the LSm ring by the 3ʹ end of U6 RNA, is oriented perpendicularly to PRP8^HB^ in pre-B^act-OTS^, contrasting with its parallel alignment with PRP8^HB^ in pre-B^act-1^ (Fig. 2b, c and Supplementary Fig. 4e). In addition, the CDC5L Myb domains, stably bound to PRP8^RT^ in pre-B^act-1 9^, remain flexible in pre-B^act-OTS^. Our crosslinking data suggest that the region of RED preceding its PRP8^HB^-bound C-terminal α helix is located close to the binding site of the CDC5L Myb domains at PRP8^RT^ in pre-B^act-OTS^ (Supplementary Fig. 5d and Supplementary Data 2). Thus, RED may also hinder, at least in part, the stable binding of CDC5L^Myb^ in pre-B^act-OTS^. Nevertheless, we were able to map a short α helix of CDC5L (aa 147–159) that bridges the NTC protein CTNNBL1 and the long α helix of SKIP (bound to PRP8^RT^), suggesting that initial contacts between CDC5L and the early activated spliceosome involves this bridge (Supplementary Fig. 6a, b).

Interestingly, CTNNBL1’s HEAT domain exhibits extensive crosslinks to other NTR proteins such as PPIL2, which is already docked in an intermediate position at SNU114 in pre-B^act-OTS^ (Supplementary Fig. 6c), and even to numerous IBC proteins, which are also abundant in our spliceosome preparation, but not yet stably positioned (Supplementary Fig. 6d). Taken together, these results indicate that, in pre-B^act-OTS^, certain NTC/NTR proteins are positioned at an intermediate assembly stage, poised to adopt their positions in pre-B^act-1^ (see also below).

### The SF3B6/SF3B1^394-430^ module as a molecular switch, operated by CDK11 kinase

**Fig. 5:**
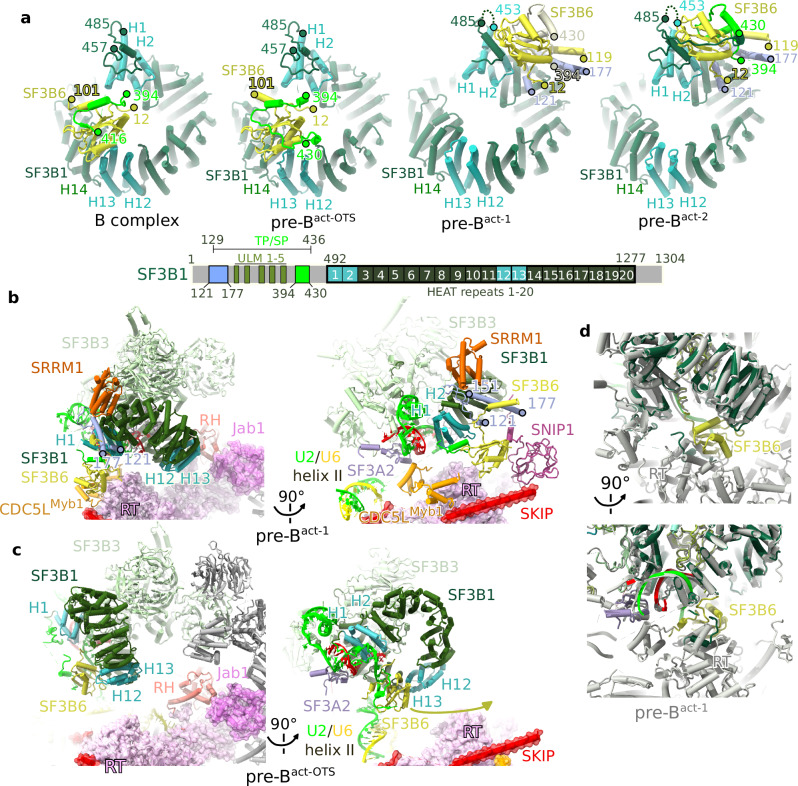
Repositioning of SF3B6 at SF3B1^HEAT^ as a prerequisite for pre-B^act-1^ formation. **a** Structural comparison of B (PDB:8QO9)^35^, pre-B^act-OTS^, pre-B^act-1^ (PDB:7ABG) and pre-B^act-2^ (PDB:7ABI)^9^ reveals a translocation of SF3B6 across SF3B1^HEAT^ during the transition from pre-B^act-OTS^ to pre-B^act-1/2^. During the pre-B^act-OTS^ to pre-B^act-1^ transition, SF3B6 moves as a single unit with the interacting SF3B1 part (aa 394–430). Residues 121–177 (blue-gray) and 394–430 (green) were not modeled in the original pre-B^act-1^ structure, but are visible in the experimental EM map (EMD-11695)^9^. Schematic representation of the domain organization of SF3B1 is shown below. Phosphorylation sites (TP/SP) cluster between residues 129 and 436. ULM, UHM ligand motif; HEAT, Huntington, Elongation Factor 3, PR65/A, TOR domain. **b**,** c** Translocation of SF3B6 from SF3B1 HEAT repeats (HRs) 12–14 in the pre-B^act-OTS^ complex to HRs 1–2 in the pre-B^act-1^ complex. The translocation of SF3B6 is accompanied by the docking of SF3B1^HEAT^ to PRP8^RH^. The SNIP1-mediated recruitment of the RES complex to pre-B^act-1^ complex further stabilizes the new position of SF3B6 at HRs 1–2. **d** The retention of SF3B6 at SF3B1 HRs 12–14 in pre-B^act-OTS^ is incompatible with the formation of pre-B^act-1^. The structure of SF3B6, bound to SF3B1 HRs 12–14 in pre-B^act-OTS^ (colored model), is superimposed upon the pre-B^act-1^ structure (shown in gray) by alignment through SF3B1^HEAT^. The overlay shows that the association of SF3B6 on SF3B1 HRs 12–14 would prohibit the docking of SF3B1^HEAT^, as SF3B6 would clash with PRP8^RT^.

The results described so far reveal the remodeling events of the spliceosome that occur after BRR2-mediated activation of the B complex, up to the stage where CDK11 phosphorylates the N-terminal region of SF3B1 (aa 1–490, SF3B1^N^, Fig. 5a). We next addressed the question of how the phosphorylation of SF3B1^N^ could trigger the progression of the activation phase of the spliceosome. Searching for a structural basis for this connection, we noticed that the position of the SF3B6 protein—which contains an RNA recognition motif, RRM—at SF3B1^HEAT^ is strikingly different in pre-B^act-OTS^ compared with in pre-B^act-1^. In pre-B^act-OTS^, a C-terminal region within SF3B1^N^ comprising aa 394–430 wraps around the RRM domain of SF3B6, forming a module that we term SF3B6/SF3B1^394-430 ^^10,13,37,38^ (Fig. 5a and Supplementary Fig. 7a). In early spliceosomes such as A-like, pre-B and B complexes, SF3B6/SF3B1^394-430^ is associated with SF3B1 HEAT repeats (HRs) 12–14 (Fig. 5a)^35,38,39^. In pre-B^act-1^, it is translocated to the N-terminal HRs 1–2, thus freeing HRs 12–14 for docking onto PRP8^RH^ (Fig. 5a, b and Supplementary Movie 4). In contrast, pre-B^act-OTS^ retains the SF3B6/SF3B1^394-430^ module at HRs 12–14 (Fig. 5a, c), and this retention creates steric constraints: during progression to pre-B^act-1^, SF3B1^HEAT^ would have to traverse PRP8^RT^ to dock onto PRP8^RH^, but the bulky SF3B6/SF3B1^394-430^ module at HRs 12–14 would certainly clash with PRP8^RT^ (Fig. 5b–d and Supplementary Movie 5). These architectural constraints explain why in pre-B^act-OTS^ the movement of the U2 5*’* domain is transiently stalled before its passage along the PRP8^RT^ domain.

The above results indicate that pre-B^act-OTS^ can only be transformed to pre-B^act-1^ once the SF3B6/SF3B1^394-430^ module has been translocated from HRs 12–14 to HRs 1–2 of SF3B1^HEAT^. Given that SF3B6 is directly associated with part of SF3B1^N^ (i.e., aa 394–430), we may further conclude that the CDK11-dependent phosphorylation of SF3B1^N^ is the major driving force for triggering this repositioning, and thus for controlling the molecular switch of the SF3B6/SF3B1^394-430^ module (see also below).

### Structural organization of SF3B1^N^ in pre-B^act-OTS^ and its anchoring between SF3B1^HEAT^ and PRP8^RT^

As the structural basis of the molecular switch of the SF3B6/SF3B1^394-430^ module is only poorly understood, we first aimed for a better understanding of the structural organization of the N-terminal domain of SF3B1 and its location in pre-B^act-OTS^. AF3 predicts that SF3B1^N^ is predominantly unstructured, yet our crosslinking data revealed a surprising degree of organization of this region. Instead of interacting randomly with other core spliceosome proteins (as would have been expected for a highly disordered protein domain), the central region of SF3B1^N^ (aa 120–340) exhibits numerous crosslinks with the SF3B6/SF3B1^394-430^ module (Supplementary Fig. 7a and Supplementary Data 2). Our intramolecular crosslinks further reveal that the SF3B1^N^ central region crosslinks extensively within itself (Supplementary Fig. 7a), suggesting that in pre-B^act-OTS^ it may form a flexible, yet compact, intramolecular protein network that interacts dynamically with the SF3B6/SF3B1^394-430^ module.

While the ca. 100 N-terminal residues of SF3B1 are apparently not involved in this intramolecular network, our crosslinks suggest that they may reach out to PRP8^RT^ (Supplementary Data 2). Indeed, AF3 predicts a high-confidence interaction of SF3B1 (aa 30–100) with PRP8^RT^ at residues around 1400–1500 (Supplementary Fig. 7b, c). Moreover, major parts of the predicted SF3B1 model (aa 30–62 and aa 92–100) can be fitted into our EM density (Supplementary Fig. 7b). We note that the binding of SF3B1^30-100^ to PRP8^RT^ is mutually exclusive with the binding of PRP6^190-208^ in the B complex, and with SKIP^405-421^ in later pre-B^act^ complexes (Supplementary Fig. 7d), indicating that SF3B1^30-100^ is anchored to PRP8^RT^ only after the large-scale translocation of the U2 5*’* domain during the B-to-pre-B^act-OTS^ transition. This anchoring would restrict the location of the central SF3B1^N^ network between the SF3B6/SF3B1^394-430^ module at the HEAT domain and the opposing PRP8^RT^ domain (Supplementary Fig. 7a). Thereby, SF3B1^N^ may re-orient its TP/SP-rich central domain, thereby presenting a high-affinity binding site for the CDK11 kinase, facilitating its recruitment, and thus allowing efficient phosphorylation of SF3B1^N^ (see Discussion below).

### A possible dual role of SF3B1^N^ phosphorylation

To gain insight into the potential role(s) of the phosphorylation of SF3B1^N^ in driving the relocation of the SF3B6/SF3B1^394-430^ module along SF3B1^HEAT^, we first used mass spectrometry to map the SF3B1 phosphopeptides isolated from PM5-10 B^act^ complexes. 20 phosphorylated residues could be mapped and, except for serine residues 190, 194 and 322, all other phosphorylated residues displayed the preferred S/T-Pro recognition motif for CDK family kinase phosphorylation (Supplementary Fig. 8a). Notably, the majority of phosphorylation sites were clustered in the central region of SF3B1^N^, suggesting that these modifications may disrupt the SF3B1^N^ intramolecular network that we observed in pre-B^act-OTS^, thus destabilizing the anchoring of at least part of the SF3B1 N-terminus and the SF3B6/SF3B1^394-430^ module at PRP8^RT^ and SF3B1^HEAT^, respectively. Consistent with this idea, we observed significantly fewer intramolecular crosslinks within SF3B1^N^ in pre-B^act-1^ and pre-B^act-2^ complexes^9^ (Supplementary Fig. 7a), where the SF3B6/SF3B1^394-430^ module had translocated to HRs 1–2. Furthermore, no crosslinks were detected between the SF3B1 N terminus and the PRP8^RT^ in the latter complexes^9^.

Of particular interest are the phosphorylations that were detected at threonine residues within SF3B1^394-430^ (T426) or directly adjacent to it (T434 and T436; Supplementary Fig. 6e), for the following reasons. In pre-B^act-OTS^, these amino acids are located at the interface of SF3B6/SF3B1^394-430^ with HR 12 of SF3B1^HEAT^ (Supplementary Fig. 8b). Thus, their modifications may destabilize the binding of the SF3B6/SF3B1^394-430^ module to the HRs 12–14 and facilitate its translocation towards HRs 1–2. Notably, following the repositioning of the SF3B6/SF3B1^394-430^ module to HRs 1–2, the three phosphothreonine residues are located in pre-B^act-1/2^ and B^act^ at the interface with the RES protein SNIP1 (Supplementary Fig. 8b). This raises the interesting possibility that the phosphorylation of T426, T434 and T436 by CDK 11 may facilitate the SNIP1-mediated recruitment of the RES complex to SF3B6/SF3B1^394-430^, and thus stabilize the binding of the latter module at the N-terminal region of SF3B1^HEAT^. Consistently with this, RES proteins are entirely absent from our pre-B^act-OTS^ spliceosome preparation (Supplementary Data 1), while in pre-B^act-1^, SNIP1 is the only protein of the RES complex stably associated with the spliceosome, which is located at the interface with SF3B1^394-430^ (Fig. 5b and Supplementary Fig 8b). In summary, it appears that SF3B1^N^ phosphorylation may have a dual role: driving the relocation of the SF3B6/B1^N^ and facilitating recruitment of RES protein SNIP1. At the same time, the RES protein(s) may stabilize the docking of the SF3B6/SF3B1^394-430^ module to HRs 1–2 and drive the progression of the spliceosome remodeling towards B^act^ formation.

### Location of DHX15 helicase and G-patch proteins at the SF3B1 HEAT domain

**Fig. 6:**
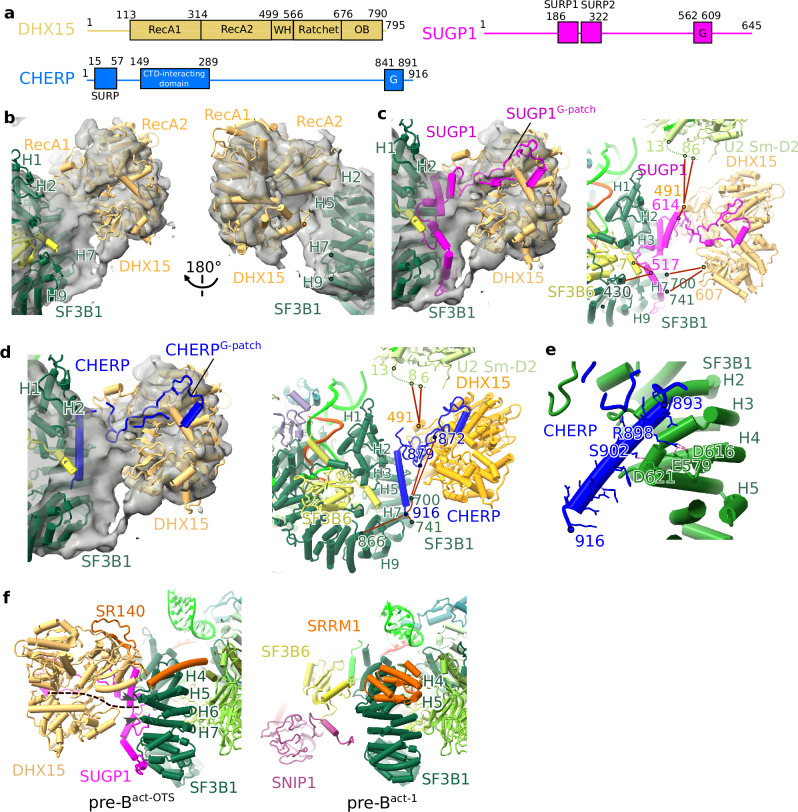
Location of DHX15 and G-patch proteins at SF3B1^HEAT^ in pre-B^act-OTS.^ **a** Schematic depictions of domain organizations of DHX15, CHERP and SUGP1; WH winged helix; OB oligonucleotide/oligosaccharide-binding (OB)-fold domain; G G-patch domain. **b** Two views of the EM density with the model of DHX15 fitted. The map is low-pass filtered to ~15 Å**. c** The EM density fitted with the AF3-predicted structure of the SUGP1-SF3B1 complex^31^. The AF3-predicted model is consistent with crosslinked residues (colored circles with brown connecting lines). **d** The EM density of pre-B^act-OTS^ fitted with the AF3-predicted structure of the CHERP-SF3B1^HEAT^ complex. The long α helix of CHERP interacts with HRs 2, 4 and 5 of SF3B1^HEAT^, and points towards HR 9. Crosslinks are shown as in (**c**) and the AF3-predicted CHERP-SF3B1^HEAT^ model is consistent with the crosslinks. **e** Atomic interactions of CHERP and SF3B1 as predicted by AF3. Dashed pink lines, hydrogen bonds. **f** Mutually exclusive binding of DHX15 and its associated G patch protein (such as SUGP1, as shown here) to the N-terminal domain of SF3B1^HEAT^ in pre-B^act-OTS^, and the SF3B6/SF3B1^394-430^ module (as well as SNIP1 and SRRM1) to the same region in pre B^act-1^. In pre B^act-1^ (and later complexes of the activation phase, including B^act^), the binding of the SF3B6/SF3B1^394–430^ module to HRs 1–2, and the accompanying recruitment of the RES complex is mutually exclusive with the binding of DHX15. Arrow head, exit channel of the intron downstream of the branch site at SF3B1^HEAT^; dashed line, RNA binding channel of DHX15’s helicase domain.

Our results so far suggest that CDK11 kinase regulates the SF3B6/SF3B1^394-430^ module as a molecular switch by controlling its repositioning across the SF3B1 ^HEAT^ domain. In this study, we blocked the phosphorylation of SF3B1^N^ by CDK11, so that SF3B6 could not translocate to the N-terminal HRs of SF3B1^HEAT^. Yet our cryo-EM map reveals a triangular-shaped density that is associated with SF3B1’s N-terminal HEAT domain. Interestingly, guided by crosslinks, we were able to fit the model of the DHX15/PRP43 RNA helicase, which is highly abundant in our pre-B^act-OTS^ preparation, into this density element (Fig. 6a, b and Supplementary Data 2).

Given the presence of unassigned density between the DHX15 model and the adjacent SF3B1 HRs 2–9, we were interested in seeing whether a G-patch protein might also be associated with DHX15 in pre-B^act-OTS^. Notably, three G-patch proteins (SUGP1, CHERP and SPF45) are abundant in our pre-B^act-OTS^ preparation (Supplementary Data 1). Indeed, the model of the DHX15/SUGP1 dimer can be fitted into the triangular-shaped density element of our pre-B^act-OTS^ map, such that the flanking structural elements of the SUGP1 G-patch are positioned in the same region of the HEAT domain as predicted in the trimeric [DHX15-SUGP1-SF3B1^HEAT^] model of Zhang et al^31^. (Fig. 6c). However, although our crosslink data support this modeling (Fig. 6c and Supplementary Data 2), the poorly resolved density between DHX15 and the SF3B1^HEAT^ prevents precise positioning of SUGP1’s α helices, which could arise from sub-stoichiometric SUGP1 binding and/or structural flexibility of the DHX15-SUGP1 complex. However, our data are also compatible with an alternative picture, involving competitive G-patch protein interactions. We noticed several crosslinks between the C-terminal region of CHERP (immediately downstream of its G-patch domain) and residues of the SF3B1 HRs 5–7 (Supplementary Data 2). AF3-based modeling revealed that the C-terminal region of CHERP can indeed form an α helix that binds to HRs 3–4, and can alternatively be docked into our pre-B^act-OTS^ map, consistent with our crosslinks (Fig. 6d, e). Obviously, the binding of CHERP and that of SUGP1 to the SF3B1^HEAT^ are mutually exclusive. It is therefore conceivable that our pre-B^act-OTS^ complex preparation contained distinct populations that share a very similar core structure but have different G-patch-mediated DHX15-SF3B1 connections. We note that our crosslinking dataset also shows crosslinks between SPF45 and DHX15 (Supplementary Data 2), but not with SF3B1^HEAT^. Whether SPF45 may recruit DHX15 to SF3B1 in the context of an aberrantly remodeled early activated spliceosome remains to be seen. As discussed below, SPF45 could also play a role in facilitating the productive interaction of CDK11 kinase with pre-B^act-OTS^ -stage spliceosomes. Previous studies have shown that SPF45 exerts additional functions in pre-mRNA splicing, such as promoting spliceosome formation on very short introns^40,41^, or inducing exon 6 skipping in the FAS receptor pre-mRNA^42^, roles in which SPF45 has to act at early stages of spliceosome formation, before the activation step.

We further note that the docking of DHX15 to the N-terminal HEAT repeats is mutually exclusive with the binding of the SF3B6/SF3B1^394-430^ module to this region (Fig. 6f). Thus, our data indicate that DHX15 may be involved in a quality check of the early, activated spliceosome in competition with the CDK11-kinase-mediated phosphorylation of SF3B1^N^. More specifically, impaired or delayed repositioning of the SF3B6/SF3B1^394-430^ module–potentially arising from aberrant spliceosome remodeling–may activate the DHX15 helicase-dependent proofreading system (see also Discussion below).

### Interaction of SR140 with outer surfaces of pre-B^act-OTS^ and SR140’s targeting of G-patch proteins to the spliceosome

Recent studies have revealed that the G-patch proteins CHERP and SPF45 form a stable, functional complex with the SR140 protein (also termed U2 SURP)^43–45^ in human cells. Moreover, RNAi-mediated knockdown of any of the three proteins led to extensive alterations of alternative splicing and cryptic splicing events^43,44^, suggesting, among other things, a proofreading role of this module in pre-mRNA splicing. Notably, SR140 is also abundant in our purified pre-B^act-OTS^ complex (Supplementary Data 1), raising the possibility that SR140 has targeted CHERP and SPF45 to the OTS-stalled spliceosome as a preformed complex. Consistent with this idea, our crosslinking results with the purified pre-B^act-OTS^ complex showed numerous crosslinks between CHERP and SPF45 and the middle region of SR140 (aa 360–680; Supplementary Fig. 9a, b and Supplementary Data 2). Notably, AF3 predicts a high-confidence model in which CHERP^631-740^ binds the SURP domain, and SPF45^80-140^ associates with the CID (CTD interacting domain) of SR140, forming an elongated structural module (Supplementary Fig. 9b–d). The predicted inter-protein interactions agree well with our crosslinks, and also with the interactions in vitro between recombinant proteins (Supplementary Fig. 9b, e). Interestingly, our integrated structural approaches indicate that SUGP1, the third abundant G-patch protein in pre-B^act-OTS^, binds via its SURP domain (SUGP1^SURP^) to the N-terminal side of SR140’s RRM domain (Supplementary Fig. 10a–c). As the binding sites for the three G-patch proteins at SR140 do not overlap (Supplementary Fig. 10d), a single SR140 protein molecule may provide a scaffold for several G-patch proteins in the pre-B^act-OTS^ complex.

**Fig. 7:**
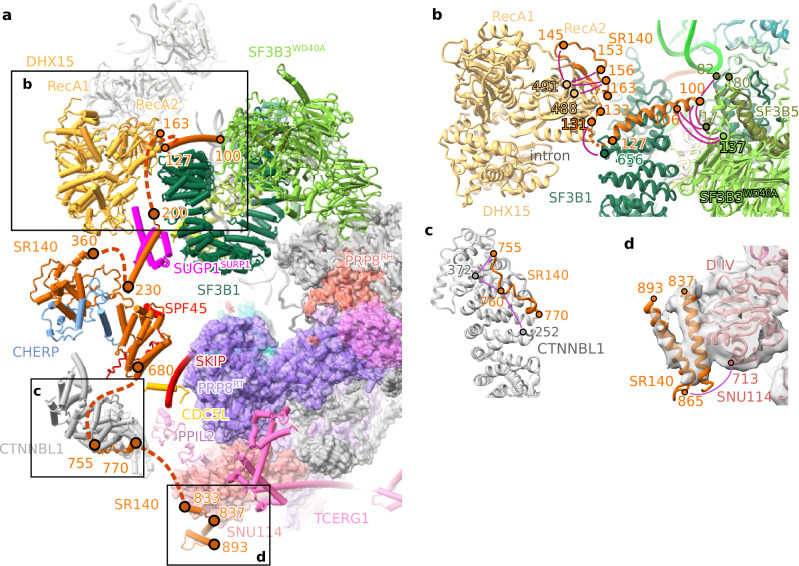
Path of SR140 at the pre-B^act-OTS^ complex and SR140’s targeting of G-patch proteins to the spliceosome. **a** An overview of the path of SR140 at the pre-B^act-OTS^ complex based on crosslinks and combined with AF3 structure predictions. The boxed regions are expanded in (**b**–**d**). The AF3-predicted models are validated by protein crosslinking coupled with mass spectrometry analysis. Crosslinked residues are all depicted as colored circles connected by pink lines. **b** The AF3-predicted complex of DHX15-SR140^100–160^-SF3b core (containing SF3B1^HEAT^, SF3B3^WD40A^, SF3B5 and PHF5A^70^) is consistent with crosslinks. **c** The AF3-predicted SR140-CTNNBL1 complex is consistent with crosslinks. **d** The AF3-predicted SR140 (aa 837–893)-SNU114 complex can be docked into the EM density, consistent with the crosslink.

Next, we investigated how SR140 might probe the structure of the stalled pre-B^act-OTS^ spliceosome. Indeed, crosslinking data, combined with AF3 predictions, delineate an extended path of SR140 at the periphery of pre-B^act-OTS^ (Fig. 7a, Supplementary Fig. 11a and Supplementary Data 2). Guided by our crosslinks, AF3 predicts a direct interaction between an SR140 α helix (aa 100–127) and HRs 4–5 of SF3B1^HEAT^, with its N terminus contacting SF3B3^WD40A^ and SF3B5 (Fig. 7b and Supplementary Fig. 11b). Adjacent residues (aa 133–163) form a β hairpin that extends the central β sheet of DHX15’s RecA2 domain (Fig. 7b and Supplementary Fig. 11a, c), probably stabilizing the docking of DHX15 to SF3B1^HEAT^.

While the central SR140 region (interacting with G-patch proteins, as shown above) showed no crosslinks to the spliceosome core, lowering the map threshold revealed a density extending from DHX15 to CTNNBL1. This density probably contains SUGP1^SURP^ bound to SR140, as well as the structural module formed by SR140^360-680^, CHERP^631-740^ and SPF45^80-140^ (Supplementary Fig. 11d). Thus, SR140 appears to position several G-patch proteins near SF3B1^HEAT^, enabling their G-patch motifs to engage DHX15 competitively.

The SR140 region, C-terminal to its CID domain, extends along SKIP and CTNNBL1, supported by crosslinks and a high-confidence AF3-predicted interaction of SR140 (aa 755–770) with CTNNBL1 (Fig. 7c and Supplementary Fig. 11e). Further to the C terminus, AF3 predicted a high-confidence interaction between a V-shaped α-helical module of SR140 (aa 830–900) and domain IV of SNU114, matching our crosslinks (Fig. 7d and Supplementary Fig. 11f). In strong support of this prediction, the α-helical module of SR140 fitted well into a corresponding density element associated with SNU114 in our map (Fig. 7d). Notably, this interaction is mutually exclusive with the binding of a very similar V-shaped α-helical element of SRRM2 to the same site in B^act^–even mirroring the atomic interactions (Supplementary Fig. 11f)^10^–indicating that SR140 can only anchor at SNU114 domain IV, before SRRM2 docks during the transition from the pre-B^act-2^ to the B^act^ complex^9^.

The recognition of specific exterior surface elements of pre-B^act-OTS^ by the SR140–G-patch protein complex is reminiscent of the probing of exterior structural elements of the ILS by the TFIP11/PAXBP1 heterodimer (Supplementary Fig. 12)^46^. However, the extended paths of the two protein complexes along their respective spliceosomes differ significantly (Supplementary Fig. 12). Moreover, the binding of TFIP11/PAXBP1 and that of SR140/G-patch proteins to their respective spliceosome complexes are mutually exclusive, which is consistent with the absence of TFIP11/PAXBP1 in our pre-B^act-OTS^ spliceosome preparation.

## Discussion

Phosphorylation of the N-terminal region of the human U2 snRNP protein SF3B1 (SF3B1^N^) by the kinase CDK11/cyclin L/SAP30BP is a crucial regulatory step in the transformation of the human B complex into the activated spliceosome, B^act^^18^. However, it has remained unclear whether SF3B1 phosphorylation is required for the BRR2 RNA helicase-mediated activation step or whether it regulates a later stage of the activation process. To address this, we determined the cryo-EM structure of a spliceosome stalled by the CDK11 kinase inhibitor OTS964, which specifically blocks phosphorylation of SF3B1^17,18^.

Our structure reveals a previously unobserved assembly intermediate, pre-B^act-OTS^, that forms after BRR2-mediated dissociation of U4 snRNP but before formation of the pre-B^act-1^ complex^9^. In pre-B^act-OTS^, U6 snRNA has formed an extended ISL (Fig. 3a, b). The energetically favorable folding of this extended U6 ISL probably drives the translocation of the U2 snRNP 5*’* domain from its peripheral position in the B complex toward the spliceosome core. This movement juxtaposes U2 stem-loop 1 (SL1) with the catalytic AGC triad (A53-G54-C55) at the base of the U6 ISL, poising these elements for catalytic U2/U6 helix Ib formation upon subsequent phosphorylation of SF3B1^N^ and transition of pre-B^act-OTS^ to pre-B^act1/2 9^ (Fig. 3e). Our structure also reveals novel SMU1/RED functions: orchestrating the coupled translocation of the U2 5*’* domain with BRR2 movement during the B-to-pre-B^act-OTS^ transition, while stabilizing U2 snRNP in the latter state (Fig. 4a, b, Supplementary Fig. 5b, c and Supplementary Movies 2, 3). Additionally, RED’s terminal regions transiently bind PRP8^HB^ and PRP8^RT^ and regulate stepwise NTC recruitment during the early spliceosome activation (Fig. 4c and Supplementary Figs. 5d, and 6a–c). That is: Our pre-B^act-OTS^ snapshot visualizes the handover of two outgoing proteins (SMU1/RED, which were recruited at the B complex stage), for new, incoming proteins–such as NTC and IBC proteins–that are positioned in pre-B^act-OTS^ at intermediate spliceosomal docking sites (Fig. 4, and Supplementary Figs. 5 and 6). The provisional docking of NTC/IBC proteins will presumably enhance the kinetics of their final integration into the activated spliceosome–a principle that may be expected to apply to the exchange of many other proteins during the entire activation phase.

A hallmark of the pre-B^act-OTS^ structure is its retention of the SF3B6/SF3B1^394-430^ module at SF3B1 HRs 12–14, contrasting with the situation in pre-B^act-1^ complexes, where SF3B6/SF3B1^394-430^ has translocated to the N-terminal HRs 1–2 (Fig. 5b, c). This retention sterically blocks further translocation of the U2 5*’* domain, explaining its transient position found in the pre-B^act-OTS^ complex. Our structure therefore indicates that the phosphorylation of SF3B1^N^ by CDK11 is then required to drive SF3B6/SF3B1^394-430^ translocation to HRs 1–2, allowing stable docking of U2 snRNP to PRP8 and the progression to pre-B^act-1^. Thus, the SF3B6/SF3B1^394-430^ module acts as a molecular switch, which is operated by CDK11. In this subsequent complex, the repositioned SF3B6/SF3B1^394-430^ module at HRs 1–2 is further stabilized by concomitant RES complex and SRRM1 recruitment (Fig. 5b and Supplementary Fig. 8b, and Supplementary Movie 1). Interestingly, in pre-B^act-OTS^, the three threonine residues T426, T434 and T436 of SF3B1 are located close to the HR 12, while in later pre-B^act^ and B^act^ complexes they are located at the SNIP1 binding interface near the relocated SF3B6/SF3B1^394-430^ module (Supplementary Fig. 8b). Therefore, phosphorylation of these threonine residues by CDK11 might fulfill a dual role: destabilizing the binding of SF3B6/SF3B1^394-430^ at HRs 12–14 in the pre-B^act-OTS^ state, and facilitating the SNIP1-mediated recruitment of the RES complex upon relocation of SF3B6/SF3B1^394-430^ to the N-terminal HRs of SF3B1 during pre-B^act-1^ formation. Consistent with our structural analysis, it has recently been shown that CDK11-mediated SF3B1 phosphorylation is functionally coupled to the SNIP1-dependent recruitment of the RES complex during B-to-B^act^ transition^47^.

SF3B6 was previously implicated as playing a role in facilitating the U2/BS helix formation during A complex assembly^38^. However, recent observations^27,48^ imply that SF3B6 is also part of a general network in which it is functionally connected to spliceosomal protein components such as RED, SMU1, RES proteins, and some NTC/NTR components that act downstream of the early E and A complexes. Consistent with this, our study shows that SF3B6 also exhibits structural connections with several of those proteins (Supplementary Fig 5d), underpinning the important role this protein plays at later assembly stages, i.e., during early spliceosome activation.

It has generally been assumed that the displacement of U4 snRNP from the spliceosome by BRR2 is the sole driving force for all the remodeling events during the B-to-B^act^ transformation^1,2^. Our work shows, however, that BRR2-mediated U4/U6 dissociation initiates only the early remodeling phase of human spliceosome activation, while the CDK11-mediated phosphorylation of SF3B1 acts as an essential molecular trigger that gates progression to later activation stages. This parallels the pre-B-to-B transition, in which PRP28 helicase initiates U6/5ʹss helix formation, but PRP4 kinase-mediated phosphorylation of PRP31/PRP6 allows extensive tri-snRNP remodeling, including BRR2 translocation^39^. Therefore, these kinase-driven protein molecular switches establish a second layer of molecular driving forces that propel the human spliceosomal cycle in addition to the conserved RNA helicases.

The observation of a CDK11-kinase-mediated SF3B6 protein switch in the human spliceosome contrasts sharply with the situation in *S. cerevisiae*, which lacks not only a CDK11 homolog, but also a homolog of human SF3B6 and a conserved N-terminal region of human SF3B1 in its yeast Hsh155 counterpart^11,49^. A bioinformatic analysis of diverse organisms revealed an interesting co-evolution of SF3B6, CDK11, and TP/SP dipeptide repeats in the N-terminal region of SF3B1 (SF3B1^N-TP/SP^) across metazoan and plants (including green algae), while these elements are missing in most yeast species, including *S. cerevisiae* (Supplementary Figs. 13 and 14). Interestingly, the intron-rich yeast *Cryptococcus neoformans* does contain an SF3B6 homolog, an SF3B1^N^ domain with a short TP-rich region, and putative homologs of CDK11 kinase, cyclin and SAP30BP^50^ (Supplementary Figs. 13 and 14). In summary, there appears to be a strong correlation between the conservation of SF3B6, a TP/SP dipeptide-rich N-terminal domain of SF3B1, and the CDK11 kinase across species during evolution; this further suggests that the CDK11-mediated SF3B6 protein switch that we describe in the human spliceosome represents a conserved regulatory mechanism in organisms with complex splicing modes. Only a few organisms (such as *S. pombe*; Supplementary Fig. 13) contain SF3B6, but do not contain a genuine homolog of CDK11^50,51^, while their SF3B1^N^ lacks TP/SP dipeptide clusters (Supplementary Fig. 14), suggesting that the relocation of SF3B6 at SF3B1^HEAT^ during early activation of the *S. pombe* spliceosome requires a distinct regulatory mechanism.

Our pre-B^act-OTS^ structure indicates that, during early activation of the human spliceosome, the SMU1/RED proteins guide the U2 5*’* domain to the intermediate checkpoint state for the phosphorylation of SF3B1^N^ by CDK11. This raised the question of whether SMU1/RED have evolved together with the CDK11-mediated SF3B6 protein switch. As shown in Supplementary Fig. 13, SMU1/RED proteins are indeed conserved in metazoans and plants, including the yeast *C. neoformans*, but are absent in all yeasts that lack the SF3B6-SF3B1^N-TP/SP^-CDK11 axis. These observations underpin the idea that SMU1/RED’s role in coordinating the remodeling (of U2 and U6 snRNPs and protein BRR2) is evolutionarily connected to the conservation of the SF3B6-SF3B1^N-TP/SP^-CDK11 axis. The absence of this axis, and of SMU1/RED, in *S. cerevisiae* suggests that the early activation phase of the yeast spliceosome follows a less complex remodeling pathway than that in humans. This accords with our earlier observation^52^ that sophisticated restructuring events in the catalytic phase of the human (but not the yeast) spliceosome involve numerous C complex proteins that are absent in S. cerevisiae.

In a recently published study, Hluchy et al^18^. blocked CDK11 kinase with OTS964 in vivo in HTC116 cells and investigated the effect of this on the global composition of thousands of distinct pre-mRNA spliceosomes that remain associated with chromatin. On the basis of retention of U4 snRNA in chromatin and reduced amounts of IBC proteins, they concluded that the chromatin-associated spliceosomes are stalled at a B-like stage, before BRR2-mediated activation. We note that chromatin-associated spliceosomes are assembled co-transcriptionally and are therefore very likely still to be tethered to the Pol II transcription machinery^53,54^. Thus, it is conceivable that one or more proteins in chromatin-associated spliceosomes might require CDK11-mediated phosphorylation before BRR2-mediated dissociation of the U4/U6 RNA helices occurs, and subsequent phosphorylation of SF3B1. Alternatively, or additionally, the release of destabilized factors such as U4 snRNP may also follow different kinetics in chromatin-associated–and thus Pol II-tethered–spliceosomes, compared with spliceosomes assembled in vitro^53,54^. Interestingly, in a more recent proteomic study, Blazek and coworkers^47^ show that certain NTR proteins, such as SKIP and RBM22, are also present in the chromatin-associated spliceosomes isolated from HTC116 cells that had been incubated with OTS964. As the binding of SKIP to spliceosomes is mutually exclusive with that of the U4 snRNA-associated PRP31 protein, this would indicate that a fraction of the chromatin-associated spliceosomes had undergone at least partial activation.

**Fig. 8:**
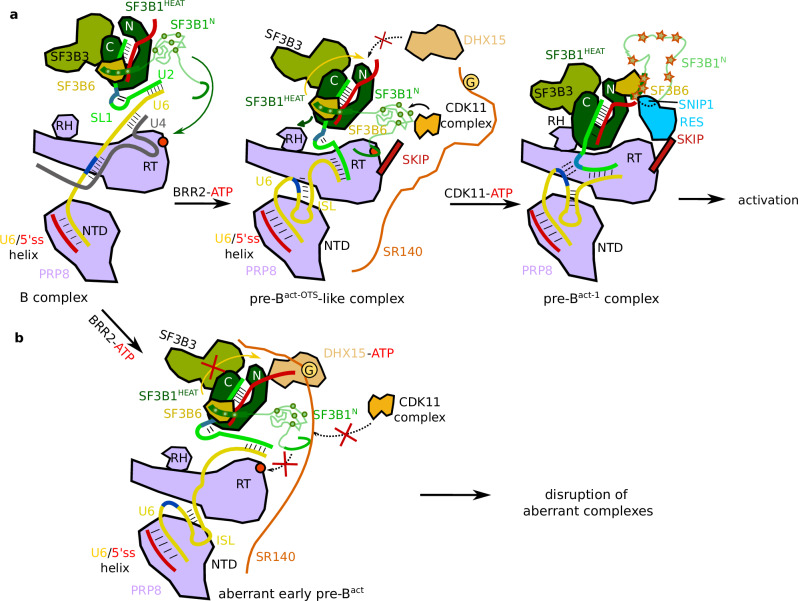
Model for the proofreading of early-activated spliceosomes by the CDK11 kinase-mediated phosphorylation of SF3B1. **a** Correctly remodeled early-activated spliceosomes pass through a pre-B^act-OTS^ –like assembly state, in which SF3B1^N^ is docked to PRP8^RT^ (and positioned between SF3B1^HEAT^ and PRP8^RT^), facilitating efficient phosphorylation of SF3B1^N^, and hence driving the relocation of SF3B6 to the N-terminal region of SF3B1^HEAT^. This will allow the SNIP1-mediated recruitment of the RES protein complex, thereby preventing the docking of DHX15 to SF3B1^HEAT^. At the same time, the SF3B1^HEAT^ domain can now pass along PRP8^RT^ and dock to PRP8^RH^, allowing the pre-B^act-OTS^-like intermediate state to progress to pre-B^act-1^ and thereafter to B^act^ formation. Red circle within the PRP8^RT^ domain: docking site for the N-terminal region of SF3B1^N^; green circles within SF3B1^N^ of pre-B^act-OTS^ –like complex and red asterisks within the pre-B^act-1^ complex indicate non-phosphorylated and phosphorylated serine or threonine residues, respectively. Red lines, pre-mRNA pieces. **b** Aberrant remodeling of the spliceosome during early activation will impair or block efficient phosphorylation of SF3B1^N^ and thus retain the SF3B6 module at the C-terminal position of SF3B1^HEAT^. This will allow time for docking of DHX15 to the N-terminal region of SF3B1^HEAT^, and, assisted by the SR140-G-patch protein complex (see text for details), lead to the dismantling of the aberrant spliceosome. In the example shown here, we envisage aberrant remodeling of U6 snRNA, which will hinder the movement of the U2 5*’* domain towards the core of the spliceosome, affecting the correct positioning of SF3B1^N^ and thus its phosphorylation by CDK11. Yellow circle, labeled G: a G-patch protein (such as SUGP1), associated with the SR140 protein. It is widely believed that quality-control mechanisms operate predominantly in spliceosomes formed on pre mRNA introns with aberrant splice or branch sites^22^. However, in view of the substantial structural remodeling events during the activation phase, such remodeling could well occur in an aberrant manner, even when canonical splice sites and branch sites are present. Aberrant (weak) 5*’*SS/U6 or BS/U2 RNA helices would likely enhance the probability of incorrect remodeling during early activation of the spliceosome.

The pre-B^act-OTS^ structure further reveals the DHX15 helicase close to SF3B1^HEAT^ HRs 4–7, implying that this early-activated spliceosome is subject to a quality check. Here, the CDK11-driven SF3B6/SF3B1^394-430^ module switch appears to play a central role, as the binding of this module and the accompanying recruitment of the RES complex to the N-terminal HEAT domain (see above) is mutually exclusive with that of DHX15 to the overlapping region (Fig. 6f). In our pre-B^act-OTS^ complex, SF3B1’s N-terminal HRs were available for DHX15 recruitment because blocked SF3B1^N^ phosphorylation retained the SF3B6/SF3B1^394-430^ module at HRs 12–14. Under physiological conditions, efficient SF3B1^N^ phosphorylation in correctly assembled complexes drives rapid SF3B6/SF3B1^394-430^ module translocation, outcompeting the recruitment of DHX15 and allowing catalytic activation. Conversely, impaired phosphorylation of SF3B1^N^ delays repositioning of the SF3B6/SF3B1^394-430^ module–potentially arising from aberrant spliceosome remodeling–permitting DHX15 recruitment to dismantle these complexes (Fig. 8).

Our structure also identifies SR140 as a novel DHX15 cofactor, which appears to probe the structure of the pre-B^act-OTS^ spliceosome by spanning ~22 nm of its exterior, and thus anchoring its N terminus to the U2 5*’* domain, its middle region to NTC proteins such as CTNNBL1, and its C terminus to SNU114 (Fig. 7 and Supplementary Fig. 11). Moreover, SR140 acts as an adaptable scaffold, capable of simultaneously recruiting several G-patch proteins (CHERP, SPF45 and SUGP1) to pre-catalytic spliceosomes, and thus allowing dynamic selection of the most suitable G-patch protein for proofreading to detect any aberrant early-activated spliceosome. The latter process will be influenced by the nature of the specific assembly defect of the aberrant spliceosome that is being proofread. Recent studies have revealed that SUGP1 co-operates with DHX15 in proofreading and dismantling defective spliceosomes, although without evidence for the participation of the SR140-CHERP-SPF45 complex^27–30^. While in some cases it has been suggested that such proofreading may take place during very early stages of spliceosome assembly, such as in the pre-A or A complex^30^, in our study, SUGP1 (in complex with SR140) interacts with the spliceosome much later, i.e., during spliceosome activation. It is therefore possible that the requirements for complex formation between SUGP1, the U2 5*’* domain, and DHX15 may differ at different stages of spliceosome assembly (see also below).

Our results are also consistent with a recent general network study that grouped the proteins CHERP, SPF45, SR140, and DHX15 into a single functional network^48^; SUGP1 was not included in that study. Moreover, our observations relate directly to results of studies showing that the proteins CHERP, SPF45, and SR140 act as a single functional module that is involved in regulating alternative splicing as well as in proofreading cryptic exon-junction events during splicing^43,44^. Notably, other G-patch proteins, such as RBM5 and RBM10, have also been implicated in supporting DHX15-mediated quality control of early spliceosome assembly^55,56^. For example, Damianov et al.^55^. purified U2 snRNP/branch site complexes from RNase-treated chromatin, together with DHX15 and the SR140-SPF45-CHERP protein complex, raising the possibility that the latter complex may also play a role in tethering the G-patch proteins RBM10 and/or RBM5 to SF3B1^HEAT^. As RBM5/10 proteins are absent in our pre-B^act-OTS^ complex, it appears likely that their U2 snRNP complex is derived from an earlier spliceosome assembly stage, suggesting that SR140-SPF45-CHERP may act repeatedly during different spliceosome remodeling steps en route to spliceosome activation.

Finally, our data suggest a new proofreading model that is mediated by CDK11 kinase. This differs from established proofreading models, in which RNA helicases directly detect aberrant RNPs either by rejecting them or by remodeling them into off-pathway spliceosomes and thus initiating subsequent dismantling. We hypothesize that kinase-mediated SF3B1^N^ phosphorylation serves as the critical checkpoint. We propose that CDK11 may “sense” correct SF3B1^N^ positioning, and that the tethering of SF3B1^N^ to PRP8^RT^ observed in pre-B^act-OTS^ (Fig. 8 and Supplementary Fig. 7b) could facilitate CDK11 recruitment and efficient phosphorylation. Indeed, in the B complex, where SF3B1^N^ is not yet phosphorylated^12^, SF3B1^N^ is quite remote from PRP8^RT^ ^35,57,58^. This tethering is only achievable after successful U2 5*’* domain translocation toward PRP8, which itself requires, among others, faithful U6 snRNA remodeling into the extended ISL conformation and stable docking of the remodeled RNA network at PRP8 (Figs. 3 and 8). Thus, errors in these important RNP rearrangements could prevent SF3B1^N^–PRP8^RT^ interactions and consequently might impair efficient CDK11 recruitment as well as phosphorylation of SF3B1^N^. While the mechanism of the recruitment of CDK11 to SF3B1 remains unclear, we propose that the tethering of the N-terminal domain of SF3B1 to PRP8^RT^ in pre-B^act-OTS^ may reorient its TP/SP-rich central region, thereby presenting a high-affinity interaction site for the CDK11 kinase. It remains to be seen whether, upon initial docking to SF3B1^N^, the CDK11 complex is further stabilized by additional interactions with one or more other spliceosomal proteins, thus facilitating efficient (and possibly processive) phosphorylation of the numerous TP/SP phosphorylation sites. For example, SAP30BP and SPF45 have been shown in principle to interact through their ULM and UHM modules, respectively (though in a spliceosomal context very different from ours)^41^. It is therefore tempting to suggest that the binding of CDK11 to the spliceosome could be stabilized by interactions between those two proteins. Alternatively, or additionally, cyclin L, which contains an RS-rich region, may also play a role in tethering CDK11 to the spliceosome. We anticipate that elucidating the structural basis of the targeting of CDK11 to SF3B1 will prove important for attaining a full understanding of the proposed kinase-dependent proofreading mechanism.

## Method

### MS2 affinity selection of OTS964-stalled PM5-10 spliceosome complexes

HeLa S3 cells were obtained from the Helmholtz Zentrum für Infektionsforschung, Braunschweig, Germany. They tested negative for mycoplasma. Cells were not authenticated. HeLa nuclear extracts were prepared essentially as previously described^12^. Uniformly [^32^P]-labeled, m^7^G(5′)ppp(5′)G-capped PM5-10 pre-mRNA^12^ was synthesized in vitro by SP6 runoff transcription and gel purified. For the purification of OTS964-stalled PM5-10 spliceosome complexes, m^7^G(5′)ppp(5′)G-capped PM5-10 pre-mRNA containing three MS2 aptamers at its 5′ end was pre-incubated with ten-fold molar excess of MS2–MBP fusion protein for 45 min on ice before addition to the splicing reaction; the final concentration of PM5-10 pre-mRNA in the splicing reaction was 5 nM. Splicing reactions were carried out in the presence of 1 µm OTS964 (MedChem Express), at 30 °C with 20% (v/v) nuclear extract in splicing buffer (3 mM MgCl_2_, 65 mM KCl, 20 mM HEPES-KOH pH 7.9, 2 mM ATP and 20 mM creatine phosphate), and incubated for 45 min. Splicing mixtures were then chilled on ice for 10 min, centrifuged for 15 min at 18,000 × *g* to remove aggregates and loaded onto an MBP Trap HP column (GE Healthcare). The column was washed with G-50 buffer (20 mM HEPES–KOH pH 7.9, 1.5 mM MgCl_2_ and 50 mM NaCl) and complexes were eluted with G-50 buffer containing 25 mM maltose. Eluted complexes were loaded onto a linear 5–20% (w/v) sucrose gradient prepared in G-50 buffer, centrifuged at 43,387 × *g* (RCFave) for 16 h at 3 °C (18,400 rpm, TST41.14 rotor, Thermo Fisher Scientific), and fractions were collected from the bottom of the gradient. RNA from complexes in peak gradient fractions was separated on a denaturing 4–12% NuPAGE gel (Life Technologies) and visualized by staining with SYBR Gold (Thermo Fisher Scientific). For cryo-EM analysis, eluted complexes were subjected to gradient fixation (GRAFIX)^59^ and processed further as described below. A mixture of affinity-purified PM5-10 A and B complexes (with some B^act^ complexes) was prepared as described above, except that the splicing reaction was for 8 min in the absence of OTS964 (to obtain primarily A and B complexes)^60^. Affinity-purified PM5-10 B^act^ complexes were prepared essentially as described previously^13^.

### Western blotting

For western blot analysis of OTS964-stalled PM5-10 complexes, PM5-10 A/B/B^act^ complexes and PM5-10 B^act^ complexes, 300 fmol of each complex preparation, (purified as described above), was separated on 5% PAGE gels and transferred to a Hybond P membrane. Membranes were first blocked with 5% milk in 1× TBS-T buffer (20 mM Tris-HCl, pH 7.5, 150 mM NaCl and 0.1% Tween 20) and then incubated with rabbit antibodies against human SF3B1 (1:750 dilution), and against human SNU116 (1:750 dilution)^15^. Subsequent to incubation with the primary antibodies, membranes were washed with TBS-T buffer and incubated with HRP-conjugated goat anti-rabbit IgG (1:30,000 dilution; 111-035-144, Jackson Immunoresearch). After washing, membranes were immunostained using an enhanced chemiluminescence detection kit (GE Healthcare) and the signal was visualized using an Amersham Imager 680.

### Protein–protein crosslinking

pre-B^act-OTS^ complexes were MS2 affinity-purified as described above with the following modifications: After affinity purification, eluted complexes were crosslinked with 450 μM BS3 for 35 min at 18 °C in a total volume of 1 ml. Crosslinked complexes were loaded onto a linear 5–20% (w/v) sucrose gradient and subjected to centrifugation at 43,387 × *g* (RCFave) for 16 h at 3 °C (18,400 rpm, TST41.14 rotor, Thermo Fisher Scientific). Four peak fractions containing OTS964-stalled PM5-10 complexes were pooled and ultracentrifuged at 233,364 × *g* (RCFave) for 24 h at 4 °C (75,000 rpm, S100-AT4 rotor, Thermo Fisher Scientific). The pelleted, crosslinked spliceosomes (~20 pmol) were dissolved in 50 mM ammonium bicarbonate buffer containing 4 M urea, reduced with dithiothreitol, alkylated with iodoacetamide and, after diluting the urea to 1 M, digested in-solution with trypsin (Promega). Peptides were reverse-phase extracted using Sep-Pak Vac tC18 1cc cartridges (Waters), lyophilized and subsequently dissolved in 40 µl 2% acetonitrile (ACN) and 20 mM ammonium hydroxide. Peptides were separated on an xBridge C18 3.5 µm 1 × 150 mm reverse-phase column (Waters) using a 4–36% gradient of ACN in 20 mM ammonium hydroxide over 45 min at a flow rate of 60 µl min^–1^. One-minute fractions of 60 µl were collected, vacuum dried and dissolved in 5% ACN and 0.1% trifluoroacetic acid (TFA) for subsequent uHPLC-ESI–MS/MS analysis using an Orbitrap Exploris 480 (Thermo Scientific). The mass spectrometer was coupled to a Dionex UltiMate 3000 uHPLC system (Thermo Scientific) with a PepMap C18 5 µm 300 µm x 5 mm trap (Thermo Scientific) and a custom main 34 cm C18 column (75 µm inner diameter packed with ReproSil-Pur 120 C18-AQ beads, 3 µm pore size (Dr. Maisch)). Data were collected in a data-dependent mode, using 60 min methods with a cycle time of 3 s, with five injections per sample. The MS1 and MS2 resolutions were set to either 120,000 or 60,000 and 30,000, respectively. MS2 injection time was 128 ms. Only precursors with a charge state of 3–8 were selected for MS2. Dynamic exclusion of 20 s was applied. MS data were acquired using Thermo Scientific Xcalibur (v.4.4.16.14) software.

The protein composition of the spliceosome assembled on PM5-10 pre-mRNA and stalled with OTS964 was determined using the same sample, which was not subjected to the offline C18 reverse-phase pre-fractionation. For this, the MS data were collected as above with the following changes: acquisition time, 120 min; dynamic exclusion, 22 s; MS1 resolution, 60,000; MS2 resolution 15,000; MS2 injection time, 54 ms; precursor charge state, 2–8. Thermo raw files were searched with MaxQuant (v.2.4.13.0) against a combined database of common contaminants observed in MS experiments and a UniProt human reference proteome (release 2024-01-24). Based on the MaxQuant results, a restricted protein database was compiled and used for protein–protein crosslink identification by searching the Thermo raw files with pLink (v.3.0.11)^61^. For model building, a maximum distance of 30 Å between the Cα atoms of the BS3-crosslinked lysine residues was allowed.

### Phosphopeptide analysis

Human B^act^ complexes were assembled on a PM5-10 pre-mRNA and purified as described previously^13^. Proteins were separated on a 4–12% NuPAGE gel, which was stained with Coomassie blue; bands at ~140–160 kDa were excised. Proteins were reduced with dithiothreitol (DTT), alkylated with iodoacetamide (IAA) and digested in-gel with trypsin. MS data were acquired as described above for the protein composition determination with the following modifications: acquisition time, 90 min; dynamic exclusion, 25 s; MS2 injection time, 60 ms. The search was performed with PEAKS Studio 11.0 against a UniProt human reference proteome (release 2024-10-02). Carbamidomethylation of cysteines was set as a fixed modification. The following variable modifications were considered: protein N-terminal acetylation, methionine oxidation, phosphorylation of serine/threonine/tyrosine. False discovery rate at the peptide-spectrum match level (PSM FDR) of 1% was applied. Phosphosites in peptides with a localization score, Ascore, of 20 (*p* value 0.01) or better are shown.

### EM sample preparation and imaging

For cryo-EM sample preparation, MS2-affinity purified spliceosomal complexes were subjected to the GraFix procedure^59^: the eluate was loaded onto a linear 5–20% (w/v) sucrose gradient containing G50 buffer with 0–0.15% glutaraldehyde and centrifuged at 43,387 × *g* (RCFave) for 16 h at 3 °C (18,400 rpm, TST41.14 rotor, Thermo Fisher Scientific). The gradient was then fractionated from the bottom of the gradient, and the crosslinker was quenched with 120 mM Tris-HCl pH 7.5 on ice. The fractions were analyzed by urea-PAGE, and the RNA was stained with SYBR Gold and detected by using the Fujifilm FLA-7000 system. The peak fractions containing the most spliceosomal complexes were combined, buffer-exchanged, and concentrated with an Amicon 50 kDa cut-off unit. The concentrated sample was adsorbed for 20 min onto R2/2 UltrAuFoil grids (Quantifoil) that were coated with a thin layer of home-made continuous carbon film. A volume of 3.8 μl of double-distilled water was then applied to the grids, and excess water was blotted away for 7.5 s, at 4 °C and 100% humidity, with pre-wetted filter paper, using a FEI Vitrobot system. The grids were subsequently vitrified by plunging into liquid ethane cooled to liquid-nitrogen temperature. Cryo-EM grids were imaged in a Titan Krios, operated at 300 kV, on a Falcon III detector in linear mode at a calibrated pixel size of 1.35 Å at the specimen level, with an exposure time of 1.02 s (40 movie frames) and a total dose of 39 e^–^ Å^–2^ (Supplementary Table 1). In total, 20,012 movies were acquired with Thermo Fisher EPU 2.1 in an automated manner, with defocus values from –1.0 to –2.5 μm (0.5 μm steps).

### EM data processing

The movie frames were aligned, dose-weighted, and summed by using MotionCor (v2.0)^62^, and the defocus values for the summed micrographs were estimated using Gctf^63^. Particle-picking was performed by using crYOLO^64^ with a custom-trained neural-network model. All subsequent processing was performed with RELION 3.1 (http://www2.mrc-lmb.cam.ac.uk/relion/index.php/Main_Page) unless otherwise specified. For all the estimations of resolution, cryo-EM data were split randomly into two halves for gold-standard FSC determination in RELION 3.1.

In total, about 1.5 million particles were picked and extracted in a 480 × 480 pixels box, and were binned to a 160 × 160 pixel box (3× binned, pixel size of 4.05 Å). After several rounds of reference-free two-dimensional (2D) classification, good particles showing reasonable features were retained, from which 100,000 particles were used for ab initio reconstruction in cryoSPARC^65^. The ab initio model showed a low-resolution 3D structure resembling that of a pre-B^act–1^ complex, and on the basis of this structural information the corresponding U2 region, which is less well defined, was erased using Chimera. The resulting 3D structure was low-pass-filtered to 50 Å resolution to prevent model bias, and it was then used for 3D classification in RELION 3.1. All the 1.5 million extracted particles were 3D-classified into 6 classes; the one class containing 326,431 particles that showed well-defined features was retained, and subsequently re-extracted and re-centered in a 240 × 240 pixels box (2× binned, pixel size of 2.7 Å). The re-extracted particles were subjected to a second round of 3D classification with a mask around the tri-snRNP region and classified into 4 classes.

133,573 particles in the one class that showed well-defined tri-snRNP density were then re-extracted in the original pixel size in a 400 × 400 pixels box and 3D-refined with a mask around the tri-snRNP region, yielding a structure with an average resolution of 3.5 Å. Two rounds of CTF refinement and Bayesian polishing were performed, and the average resolution was improved to 3.2 Å for the tri-snRNP core region, and 4.2 Å for the BRR2 region. To further improve the tri-snRNP core, cryoSieve^66^ was then performed with a soft mask around the tri-snRNP region (encompassing PRP8, SNU114, U6 snRNA (nts 1–84), the PM5 pre-mRNA (nts –9 to +20), U5 snRNA, RED (aa 524–557), SNU23, PRP38A, MFAP1 C-terminal region, UBL5, BUD31, TCERG1, RBM22, PPIL2, CDC5L, SKIP, and WBP11), to remove particles that did not contribute to high-resolution information. CryoSieve removed 20% of the particles for each iteration, and 3D refinement was performed for each iteration of CryoSieve to monitor its performance. The 6th iteration showed the best result, yielding a 3.12 Å structure for the tri-snRNP region with only 35,015 particles.

To improve the U2 snRNP region, the 133,573 particles were CTF-refined, Bayesian-polished, and re-scaled into a 240 × 240 pixels box (2× binned, pixel size of 2.7 Å). A masked classification without alignment was performed, focusing on the U2 snRNP region (8 classes, T = 20). The classes containing 55,345 particles that showed clear U2 snRNP features were combined. However, neither focused 3D refinement nor multi-body refinement improved the U2 snRNP structure. Therefore, the particles were imported into Relion5, and DynaMight was performed^67^. DynaMight estimates the molecular motions for each experimental particle image by a deep convolutional neural network, and then constructs an improved consensus map with better local resolution for less stable parts by back-projecting individual particle images while taking the local motions of each particle into account. For the DynaMight reconstruction, 29,000 Gaussians were used to describe the flexibility for the entire complex.

We repeated the determination of the cryo-EM structure twice, using two additional, independent pre-B^act-OTS^ preparations. This yielded two separate structures at average resolutions of 3.94 Å and 5.29 Å, respectively. These additional experiments showed structures that were essentially identical to our initial structure; however, these sets of EM data have not been included in the final cryo-EM map reconstruction.

### AlphaFold3 prediction

The AlphaFold3 server^36^ (https://deepmind.google/technologies/alphafold/alphafold-server/) was used for the prediction of 3D structures of protein complexes. For each prediction, only protein sequences were provided, without providing any external references. The structure of the SF3b core-SR140^N-terminal^-DHX15 complex was predicted by using the sequences of SF3B1 (450–1304), SF3B3 (1–1217), PHF5A (1–110), SF3B5 (1–86), SR140 (1–170) and DHX15 (110–795). The SR140–SUGP1 interaction was predicted by using the sequences of SR140 (81–280) and SUGP1 (171–340). The SR140–SPF45–CHERP complex was predicted by using the sequences of SR140 (361–680), SPF45 (51–220) and CHERP (631–740). The SR140–CTNNBL1 interaction was predicted by using the sequences of SR140 (701–800) and CTNNBL1 (80–563). The SR140–SNU114 interaction was predicted by using the sequences of SR140 (801–920) and SNU114 (101–972). The PRP8–SF3B1 interaction was predicted by using the sequences of PRP8 (full-length) and SF3B1 (1–120). The PRP8–RED interaction was predicted by using the sequences of PRP8 (full-length) and RED (491–557). Only the ‘confident’ interactions were retained (pIDDT value larger than 70).

### Model-building and refinement

Model-building was carried out by docking known cryo-EM, crystal or AlphaFold-predicted structures into the EM density. The docked models were then adjusted in COOT^68^. A complete list of modeled protein and RNA components is given in Supplementary Data 3, together with the corresponding templates used for modeling them. In brief, the model of the pre-B^act-1^ complex (PDB:7ABG)^9^ – excluding the models for the U2 snRNP, LSm, U2–U6 helix II, WBP11, the B-specific proteins (SNU23, MFAP1, PRP38, UBL5), CBC80/20, CDC5L, SNIP1 – was docked into the EM density. The models for the U2 snRNP, LSm, U2–U6 helix II, the B-specific proteins (SNU23, MFAP1, PRP38, UBL5), and the SMU1–RED heterotetramer were extracted from the B-complex structure (PDB: 8QO9)^35^. The model for PPIL2 (U-box) was extracted from the pre-B^act-2^ complex (PDB:7ABI)^9^. The model for U2 SL1 (nts 12–14; 19–21) was built as an A-form helix; the models for WBP11 (aa 50–139) and CTNNBL1 (80–563) were taken from AlphaFold prediction. The model for SF3B1 N-terminal part (aa 30–62; aa 90–104) was extracted from the AlphaFold3-predicted model of PRP8-SF3B1^N^ as described above. The model for DHX15 (aa 110–795) bound to SR140 (aa 133–163) was extracted from the AlphaFold3-predicted model of the SF3b core–SR140^N-terminal^–DHX15 complex as described above. The interaction between SR140 (aa 801–920) and SNU114 (aa 101–972) was predicted by AlphaFold3 as mentioned above. The model for SR140 (aa 833–893), for which EM density was clearly visible, was extracted from the predicted structure. All of these models were rigid-body docked into the EM density, and for the regions where the resolution did not allow side-chain modeling, the models were all truncated into poly-alanine chains. All of the individual components were then manually adjusted, corrected, and/or extended as needed, by using COOT to ensure accuracy and proper fit within the EM density. The model for the better-resolved region was then refined in real space by using PHENIX^69^. The lower-resolution parts where the models were truncated into poly-alanine chains were not refined by PHENIX.

### Plasmid construction, protein expression and purification

The PCR-amplified fragment encoding the human SR140 (aa 1–274, designated SR140^1-274^) was cloned into the pET15b vector, with N-terminal His(6)-tag followed by a MBP fusion protein and TEV cleavage site. The fragment encoding the human SUGP1 (aa 180–340, designated GST-SUGP1^180-340^) was cloned into the pGEX6P vector. For co-purification, three DNA fragments, encoding SPF45 (aa 80–125), SR140 (aa 400–680) and CHERP (aa 634–740), were sequentially cloned into an engineered pRSFduet vector, with each gene driven by an individual promoter and a ribosome binding site. In this construct, SPF45 was fused at the N terminus with an N-terminal His(6)-SUMO tag, while SR140 and CHERP were fused with N-terminal GST tags followed by a 3 C protease cleavage site.

All recombinant plasmids were transfected into *Escherichia coli* strain BL21 (DE3). The cells were grown in LB medium supplemented with 100 mg/mL ampicillin or 50 mg/mL kanamycin at 37 °C until the optical density at 600 nm (OD_600_) reached 0.6. After IPTG was added to a final concentration of 0.3 mM, the cells were cooled down and continued to grow at 18 °C overnight.

SR140^1-274^ was purified with Ni NTA Beads (Smart-Lifesciences) followed by TEV protease cleavage, then loaded onto a Hitrap Capto S column (Cytiva) with a linear gradient elute. SR140^1-274^ was further purified by gel filtration chromatography. GST-SUGP1^180-340^ was purified with glutathione beads 4FF (LABLEAD) and concentrated directly for GST pull-down assays. For co-purification of the SPF45^80-125^–SR140^400-680^-CHERP^635-740^ complex, *E. coli* cell lysate was incubated with glutathione beads 4FF (LABLEAD), followed by elution with 10 mM reduced glutathione (GSH). The eluate was then subjected to 3 C protease digestion, and was further purified with Ni-NTA resin (Smart-Lifesciences). Final purification step was performed by gel filtration, using a Superdex 200 increase column (Cytiva).

### GST pulldown assays

Purified SR140^1-274^ and GST-SUGP1^180-340^ protein fragments were used for the GST pull-down assay. Briefly, 20 μg of either GST (control) or GST-SUGP1^180-340^ and 75 μg of SR140^1-274^ were supplemented with binding buffer (20 mM Tris pH 8.0, 75 mM NaCl, 10% glycerol, 0.1% Tritonx-100) to a final volume of 0.5 mL. 15 μL of pre-equilibrated glutathione beads (Smart-Lifesciences) were then added to the mixture, followed by incubation at room temperature for 30 minutes. The beads were washed five times with 1 mL of binding buffer and subsequently boiled in 50 μL of 1 × SDS loading buffer. Bound proteins were analyzed by SDS-PAGE electrophoresis.

### Reporting summary

Further information on research design is available in the Nature Portfolio Reporting Summary linked to this article.

## Supplementary information

Supplementary Information

Description of Additional Supplementary Files

Supplementary Data 1

Supplementary Data 2

Supplementary Data 3

Supplementary Movie 1

Supplementary Movie 2

Supplementary Movie 3

Supplementary Movie 4

Supplementary Movie 5

Reporting Summary

Transparent Peer Review file

## Source data

Source data

## Data Availability

The cryo-EM maps and coordinates have been deposited into the Electron Microscopy Data Bank (EMDB) and the PDB as follows: complete structure of the pre-B^act-^*OTS* complex (EMD-53843, PDB 9R8V), core structure of the pre-B^act-OTS^ complex (EMD-53554). Source Data are provided with this paper. The B, pre-B^act-1^, pre-B^act-2^, B^act^, and *C. elegans* ILS” structures used in this study for structural comparisons are available under accession codes 8QO9, 7ABG, 7ABI, 6FF7, and 8RO1, respectively. The AlphaFold3-predicted models of SPF45-SR140-CHERP, SR140-SUGP1, SR140-CTNNBL1, and SF3b core-SR140-DHX15 complexes are available in ModelArchive (modelarchive.org) with the accession codes ma-9luyy, ma-ire8e, ma-sd0ae, and ma-m2b6q, respectively. Source data are provided with this paper.

## References

[1] Wahl, M. C., Will, C. L. & Lührmann, R. The spliceosome: design principles of a dynamic RNP machine. *Cell* **136**, 701–718 (2009). 19239890 10.1016/j.cell.2009.02.009

[2] Will, C. L. & Lührmann, R. Spliceosome structure and function. *Cold Spring Harb Perspect Biol.*10.1101/cshperspect.a003707 (2011). 10.1101/cshperspect.a003707 PMC3119917 21441581

[3] Brow, D. A. Allosteric cascade of spliceosome activation. *Annu Rev. Genet* **36**, 333–360 (2002). 12429696 10.1146/annurev.genet.36.043002.091635

[4] Madhani, H. D. & Guthrie, C. A novel base-pairing interaction between U2 and U6 snRNAs suggests a mechanism for the catalytic activation of the spliceosome. *Cell* **71**, 803–817 (1992). 1423631 10.1016/0092-8674(92)90556-r

[5] Fica, S. M. et al. RNA catalyses nuclear pre-mRNA splicing. *Nature* **503**, 229–234 (2013). 24196718 10.1038/nature12734 PMC4666680

[6] Wilkinson, M. E., Charenton, C. & Nagai, K. RNA splicing by the spliceosome. *Annu Rev. Biochem* **89**, 359–388 (2020). 31794245 10.1146/annurev-biochem-091719-064225

[7] Wan, R., Bai, R., Zhan, X. & Shi, Y. How is precursor messenger RNA spliced by the spliceosome? *Annu Rev. Biochem* **89**, 333–358 (2020). 31815536 10.1146/annurev-biochem-013118-111024

[8] Kastner, B., Will, C. L., Stark, H. & Lührmann, R. Structural insights into nuclear pre-mRNA splicing in higher eukaryotes. *Cold Spring Harb. Perspect. Biol.* **11**, a032417 (2019). 30765414 10.1101/cshperspect.a032417 PMC6824238

[9] Townsend, C. et al. Mechanism of protein-guided folding of the active site U2/U6 RNA during spliceosome activation. *Science* **370**, eabc3753 (2020). 33243851 10.1126/science.abc3753

[10] Zhan, X., Lu, Y. & Shi, Y. Molecular basis for the activation of human spliceosome. *Nat. Commun.* **15**, 6348 (2024). 39068178 10.1038/s41467-024-50785-0 PMC11283556

[11] Wang, C. et al. Phosphorylation of spliceosomal protein SAP 155 coupled with splicing catalysis. *Genes Dev.* **12**, 1409–1414 (1998). 9585501 10.1101/gad.12.10.1409 PMC316838

[12] Bessonov, S. et al. Characterization of purified human Bact spliceosomal complexes reveals compositional and morphological changes during spliceosome activation and first step catalysis. *RNA* **16**, 2384–2403 (2010). 20980672 10.1261/rna.2456210 PMC2995400

[13] Haselbach, D. et al. Structure and conformational dynamics of the human spliceosomal B(act) complex. *Cell* **172**, 454–464.e411 (2018). 29361316 10.1016/j.cell.2018.01.010

[14] Zhang, X. et al. Structure of the human activated spliceosome in three conformational states. *Cell Res*. **28**, 307–322 (2018). 29360106 10.1038/cr.2018.14 PMC5835773

[15] Girard, C. et al. Post-transcriptional spliceosomes are retained in nuclear speckles until splicing completion. *Nat. Commun.* **3**, 994 (2012). 22871813 10.1038/ncomms1998

[16] Rájecký, M. et al. CDK7-CDK11 axis in spliceosome regulation and pre-mRNA splicing. *Nucleic Acids Res.*10.1093/nar/gkaf1343 (2025). 10.1093/nar/gkaf1343 PMC12721332 41428738

[17] Wang, C. et al. CDK11 requires a critical activator SAP30BP to regulate pre-mRNA splicing. *EMBO J.* **42**, e114051 (2023). 38059508 10.15252/embj.2023114051 PMC10711644

[18] Hluchý, M. et al. CDK11 regulates pre-mRNA splicing by phosphorylation of SF3B1. *Nature* **609**, 829–834 (2022). 36104565 10.1038/s41586-022-05204-z

[19] Lin, A. et al. Off-target toxicity is a common mechanism of action of cancer drugs undergoing clinical trials. *Sci. Transl. Med*. **11**, 8412 (2019). 10.1126/scitranslmed.aaw8412 PMC7717492 31511426

[20] Mayas, R. M., Maita, H. & Staley, J. P. Exon ligation is proofread by the DExD/H-box ATPase Prp22p. *Nat. Struct. Mol. Biol.* **13**, 482–490 (2006). 16680161 10.1038/nsmb1093 PMC3729281

[21] Burgess, S. M. & Guthrie, C. A mechanism to enhance mRNA splicing fidelity: the RNA-dependent ATPase Prp16 governs usage of a discard pathway for aberrant lariat intermediates. *Cell* **73**, 1377–1391 (1993). 8324826 10.1016/0092-8674(93)90363-u

[22] Semlow, D. R. & Staley, J. P. Staying on message: ensuring fidelity in pre-mRNA splicing. *Trends Biochem Sci.* **37**, 263–273 (2012). 22564363 10.1016/j.tibs.2012.04.001 PMC3735133

[23] Koodathingal, P. & Staley, J. P. Splicing fidelity: DEAD/H-box ATPases as molecular clocks. *RNA Biol.* **10**, 1073–1079 (2013). 23770752 10.4161/rna.25245 PMC3849154

[24] Bohnsack, K. E., Ficner, R., Bohnsack, M. T. & Jonas, S. Regulation of DEAH-box RNA helicases by G-patch proteins. *Biol. Chem.* **402**, 561–579 (2021). 33857358 10.1515/hsz-2020-0338

[25] Yoshimoto, R., Kataoka, N., Okawa, K. & Ohno, M. Isolation and characterization of post-splicing lariat-intron complexes. *Nucleic Acids Res*. **37**, 891–902 (2009). 19103666 10.1093/nar/gkn1002 PMC2647322

[26] Liu, Y. C. & Cheng, S. C. Functional roles of DExD/H-box RNA helicases in Pre-mRNA splicing. *J. Biomed. Sci.* **22**, 54 (2015). 26173448 10.1186/s12929-015-0161-z PMC4503299

[27] Beusch, I. et al. Targeted high-throughput mutagenesis of the human spliceosome reveals its in vivo operating principles. *Mol. Cell* **83**, 2578–2594.e2579 (2023). 37402368 10.1016/j.molcel.2023.06.003 PMC10484158

[28] Feng, Q., Krick, K., Chu, J. & Burge, C. B. Splicing quality control mediated by DHX15 and its G-patch activator SUGP1. *Cell Rep.* **42**, 113223 (2023). 37805921 10.1016/j.celrep.2023.113223 PMC10842378

[29] Maul-Newby, H. M. et al. A model for DHX15 mediated disassembly of A-complex spliceosomes. *RNA* **28**, 583–595 (2022). 35046126 10.1261/rna.078977.121 PMC8925973

[30] Zhang, J. et al. DHX15 is involved in SUGP1-mediated RNA missplicing by mutant SF3B1 in cancer. *Proc. Natl. Acad. Sci. USA* **119**, e2216712119 (2022). 36459648 10.1073/pnas.2216712119 PMC9894173

[31] Zhang, J. et al. Characterization of the SF3B1-SUGP1 interface reveals how numerous cancer mutations cause mRNA missplicing. *Genes Dev.* **37**, 968–983 (2023). 37977822 10.1101/gad.351154.123 PMC10760632

[32] Montemayor, E. J. et al. Architecture of the U6 snRNP reveals specific recognition of 3’-end processed U6 snRNA. *Nat. Commun.* **9**, 1749 (2018). 29717126 10.1038/s41467-018-04145-4 PMC5931518

[33] Sashital, D. G., Cornilescu, G., McManus, C. J., Brow, D. A. & Butcher, S. E. U2-U6 RNA folding reveals a group II intron-like domain and a four-helix junction. *Nat. Struct. Mol. Biol.* **11**, 1237–1242 (2004). 15543154 10.1038/nsmb863

[34] Ulrich, A. K. C., Schulz, J. F., Kamprad, A., Schütze, T. & Wahl, M. C. Structural basis for the functional coupling of the alternative splicing factors Smu1 and RED. *Structure* **24**, 762–773 (2016). 27150041 10.1016/j.str.2016.03.016

[35] Zhang, Z. et al. Cryo-EM analyses of dimerized spliceosomes provide new insights into the functions of B complex proteins. *EMBO J.* **43**, 1065–1088 (2024). 38383864 10.1038/s44318-024-00052-1 PMC10943123

[36] Abramson, J. et al. Accurate structure prediction of biomolecular interactions with AlphaFold 3. *Nature* **630**, 493–500 (2024). 38718835 10.1038/s41586-024-07487-w PMC11168924

[37] Spadaccini, R. et al. Biochemical and NMR analyses of an SF3b155-p14-U2AF-RNA interaction network involved in branch point definition during pre-mRNA splicing. *RNA* **12**, 410–425 (2006). 16495236 10.1261/rna.2271406 PMC1383580

[38] Tholen, J., Razew, M., Weis, F. & Galej, W. P. Structural basis of branch site recognition by the human spliceosome. *Science* **375**, 50–57 (2022). 34822310 10.1126/science.abm4245 PMC7614990

[39] Zhang, Z. et al. Structural insights into the cross-exon to cross-intron spliceosome switch. *Nature* **630**, 1012–1019 (2024). 38778104 10.1038/s41586-024-07458-1 PMC11208138

[40] Fukumura, K. et al. SPF45/RBM17-dependent, but not U2AF-dependent, splicing in a distinct subset of human short introns. *Nat. Commun.* **12**, 4910 (2021). 34389706 10.1038/s41467-021-24879-y PMC8363638

[41] Fukumura, K. et al. SAP30BP interacts with RBM17/SPF45 to promote splicing in a subset of human short introns. *Cell Rep.* **42**, 113534 (2023). 38065098 10.1016/j.celrep.2023.113534

[42] Corsini, L. et al. U2AF-homology motif interactions are required for alternative splicing regulation by SPF45. *Nat. Struct. Mol. Biol.* **14**, 620–629 (2007). 17589525 10.1038/nsmb1260

[43] De Maio, A. et al. RBM17 Interacts with U2SURP and CHERP to Regulate Expression and Splicing of RNA-Processing Proteins. *Cell Rep.* **25**, 726–736.e727 (2018). 30332651 10.1016/j.celrep.2018.09.041 PMC6292215

[44] Martín, E., Vivori, C., Rogalska, M., Herrero-Vicente, J. & Valcárcel, J. Alternative splicing regulation of cell-cycle genes by SPF45/SR140/CHERP complex controls cell proliferation. *RNA* **27**, 1557–1576 (2021). 34544891 10.1261/rna.078935.121 PMC8594467

[45] Papasaikas, P., Tejedor, J. R., Vigevani, L. & Valcárcel, J. Functional splicing network reveals extensive regulatory potential of the core spliceosomal machinery. *Mol. Cell* **57**, 7–22 (2015). 25482510 10.1016/j.molcel.2014.10.030

[46] Vorländer, M. K. et al. Mechanism for the initiation of spliceosome disassembly. *Nature* **632**, 443–450 (2024). 38925148 10.1038/s41586-024-07741-1 PMC7616679

[47] Gajdušková, P. et al. Phosphorylation of SF3B1 by CDK11 orchestrates spliceosome activation via SNIP1-dependent RES complex recruitment. *Nat. Commun.*10.1038/s41467-026-71119-2 (2026). 10.1038/s41467-026-71119-2 PMC13194972 41904131

[48] Rogalska, M. E. et al. Transcriptome-wide splicing network reveals specialized regulatory functions of the core spliceosome. *Science* **386**, 551–560 (2024). 39480945 10.1126/science.adn8105

[49] Will, C. L. et al. A novel U2 and U11/U12 snRNP protein that associates with the pre-mRNA branch site. *EMBO J.* **20**, 4536–4546 (2001). 11500380 10.1093/emboj/20.16.4536 PMC125580

[50] Sales-Lee, J. et al. Coupling of spliceosome complexity to intron diversity. *Curr. Biol.* **31**, 4898–4910 (2021). 34555349 10.1016/j.cub.2021.09.004 PMC8967684

[51] Hluchý, M. & Blazek, D. CDK11, a splicing-associated kinase regulating gene expression. *Trends Cell Biol.* **35**, 702–716 (2025). 39245599 10.1016/j.tcb.2024.08.004

[52] Bertram, K. et al. Structural insights into the roles of metazoan-specific splicing factors in the human step 1 spliceosome. *Mol. Cell* **80**, 127–139 (2020). 33007253 10.1016/j.molcel.2020.09.012

[53] Bentley, D. L. Coupling mRNA processing with transcription in time and space. *Nat. Rev. Genet* **15**, 163–175 (2014). 24514444 10.1038/nrg3662 PMC4304646

[54] Herzel, L., Ottoz, D. S. M., Alpert, T. & Neugebauer, K. M. Splicing and transcription touch base: co-transcriptional spliceosome assembly and function. *Nat. Rev. Mol. Cell Biol.* **18**, 637–650 (2017). 28792005 10.1038/nrm.2017.63 PMC5928008

[55] Damianov, A. et al. The splicing regulators RBM5 and RBM10 are subunits of the U2 snRNP engaged with intron branch sites on chromatin. *Mol. Cell* **84**, 1496–1511.e1497 (2024). 38537639 10.1016/j.molcel.2024.02.039 PMC11057915

[56] Guerra-Moreno, Á & Valcárcel, J. AI-assisted proofreading of RNA splicing. *Genes Dev.* **37**, 945–947 (2023). 38092520 10.1101/gad.351373.123 PMC10760631

[57] Bertram, K. et al. Cryo-EM structure of a pre-catalytic human spliceosome primed for activation. *Cell* **170**, 701–713 (2017). 28781166 10.1016/j.cell.2017.07.011

[58] Zhan, X., Yan, C., Zhang, X., Lei, J. & Shi, Y. Structures of the human pre-catalytic spliceosome and its precursor spliceosome. *Cell Res* **28**, 1129–1140 (2018). 30315277 10.1038/s41422-018-0094-7 PMC6274647

[59] Kastner, B. et al. GraFix: sample preparation for single-particle electron cryomicroscopy. *Nat. Methods* **5**, 53–55 (2008). 18157137 10.1038/nmeth1139

[60] Deckert, J. et al. Protein composition and electron microscopy structure of affinity-purified human spliceosomal B complexes isolated under physiological conditions. *Mol. Cell Biol.* **26**, 5528–5543 (2006). 16809785 10.1128/MCB.00582-06 PMC1592722

[61] Chen, Z. L. et al. A high-speed search engine pLink 2 with systematic evaluation for proteome-scale identification of cross-linked peptides. *Nat. Commun.* **10**, 3404 (2019). 31363125 10.1038/s41467-019-11337-z PMC6667459

[62] Zheng, S. Q. et al. MotionCor2: anisotropic correction of beam-induced motion for improved cryo-electron microscopy. *Nat. Methods* **14**, 331–332 (2017). 28250466 10.1038/nmeth.4193 PMC5494038

[63] Zhang, K. Gctf: Real-time CTF determination and correction. *J. Struct. Biol.* **193**, 1–12 (2016). 26592709 10.1016/j.jsb.2015.11.003 PMC4711343

[64] Wagner, T. et al. SPHIRE-crYOLO is a fast and accurate fully automated particle picker for cryo-EM. *Commun. Biol.* **2**, 218 (2019). 31240256 10.1038/s42003-019-0437-z PMC6584505

[65] Punjani, A., Rubinstein, J. L., Fleet, D. J. & Brubaker, M. A. cryoSPARC: algorithms for rapid unsupervised cryo-EM structure determination. *Nat. Methods* **14**, 290–296 (2017). 28165473 10.1038/nmeth.4169

[66] Zhu, J. et al. A minority of final stacks yields superior amplitude in single-particle cryo-EM. *Nat. Commun.* **14**, 7822 (2023). 38072910 10.1038/s41467-023-43555-x PMC10711021

[67] Schwab, J., Kimanius, D., Burt, A., Dendooven, T. & Scheres, S. H. W. DynaMight: estimating molecular motions with improved reconstruction from cryo-EM images. *Nat. Methods* **21**, 1855–1862 (2024). 39123079 10.1038/s41592-024-02377-5 PMC11466895

[68] Emsley, P. & Cowtan, K. Coot: model-building tools for molecular graphics. *Acta Crystallogr D. Biol. Crystallogr* **60**, 2126–2132 (2004). 15572765 10.1107/S0907444904019158

[69] Afonine, P. V. et al. Real-space refinement in PHENIX for cryo-EM and crystallography. *Acta Crystallogr D. Struct. Biol.* **74**, 531–544 (2018). 29872004 10.1107/S2059798318006551 PMC6096492

[70] Cretu, C. et al. Molecular architecture of SF3b and structural consequences of its cancer-related mutations. *Mol. Cell* **64**, 307–319 (2016). 27720643 10.1016/j.molcel.2016.08.036

