# Supplementary material for: Structural basis of the regulation by CDK11 kinase of early spliceosome activation and evidence for its proofreading by DHX15 helicase
Source: Nat Commun. 2026 Jul 3;17:5845. doi: 10.1038/s41467-026-75109-2 (PMC13332222; doi:10.1038/s41467-026-75109-2)
Supplement: Supplementary file 1 — Supplementary Information [file 41467_2026_75109_MOESM1_ESM.pdf]

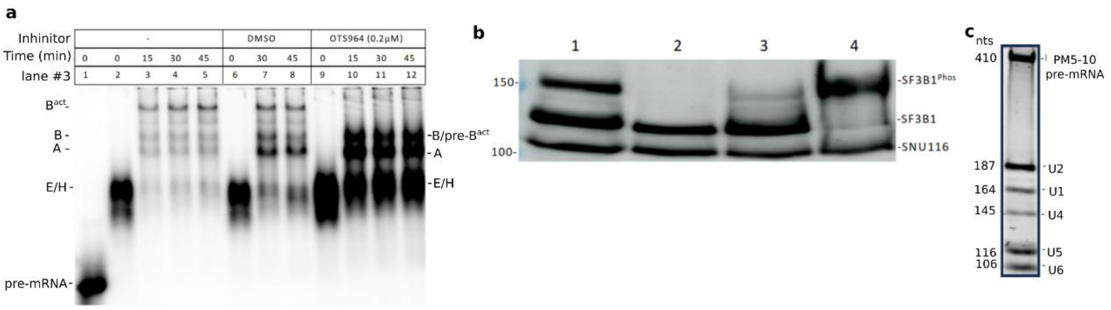

Supplementary Fig. 1 OTS964 blocks splicing during the transformation of the spliceosomal B complex into B^{act}

a, Inhibition of pre-mRNA splicing by OTS964. Splicing was performed with $[^{32}P]$-PM5-10 pre-mRNA in the presence of 0.2 µm OTS964 in HeLa nuclear extract for the times indicated. Spliceosomal complex formation was analyzed on a agarose gel. We note that pre-B^{act} complexes, comigrate in native gels together with B complexes[1]. Bands were visualized by autoradiography. DMSO, control reaction with the solvent for OTS964. Spliceosome assembly studies were repeated at least three times with similar results. Lanes 10–12 show that a significant fraction of stalled spliceosomes migrate in native gels in a manner similar to that of B/pre-B^{act} stage complexes. However, A-stage complexes also accumulate, consistently with the idea that inhibition of phosphorylation of SR proteins (such as SRSF1) by CDK11 kinase[2,3], may hinder efficient integration of U4/U6.U5 tri-snRNP into spliceosomes[4] **b**, Western blots reveal that OTS964 blocks phosphorylation of the U2 SFB1 protein. Proteins from various purified spliceosomes were analyzed by western blotting, using antibodies against the human U2 SF3B1 protein. Lane1, mixture of affinity-purified PM5-10 A, B and B^{act} complexes; lane 2, purified OTS964-stalled PM5-10 complexes; lane 3, same complex as used in lane 2, but incubated for 15 min with 2 mM ATP before protein extraction; lane 4, purified PM5-10 B^{act} complexes. $SF3B1^{Phos}$, phosphorylated SF3B1 protein. Antibodies against SNU114 were used to ensure equal loading. Western blotting was analyzed from three independent preparations of the various spliceosomal complexes with similar results. **c**, RNA composition of purified PM5-10 pre-$B^{act\text{-}OTS}$ complexes. OTS964-stalled spliceosomes, formed on PM5-10 pre-mRNA, were affinity-purified and subjected to gradient centrifugation; RNA from fractions of the fastest sedimenting

peak was isolated, separated on a denaturing NuPAGE gel, and visualized by staining with SyBr gold. The RNA composition was analyzed from three independent OTS964-stalled complex purifications with similar results. The remaining amounts of U4 snRNA indicate that some U4/U6.U5 tri-snRNP-containing populations of spliceosomes are also present in our pre-$B^{act\text{-}OTS}$ preparation, consistent with the presence of U4/U6.U5 tri-snRNP proteins such as SAD1, SNU66 and DIM1 in our MS analysis (Supplementary Data 1).

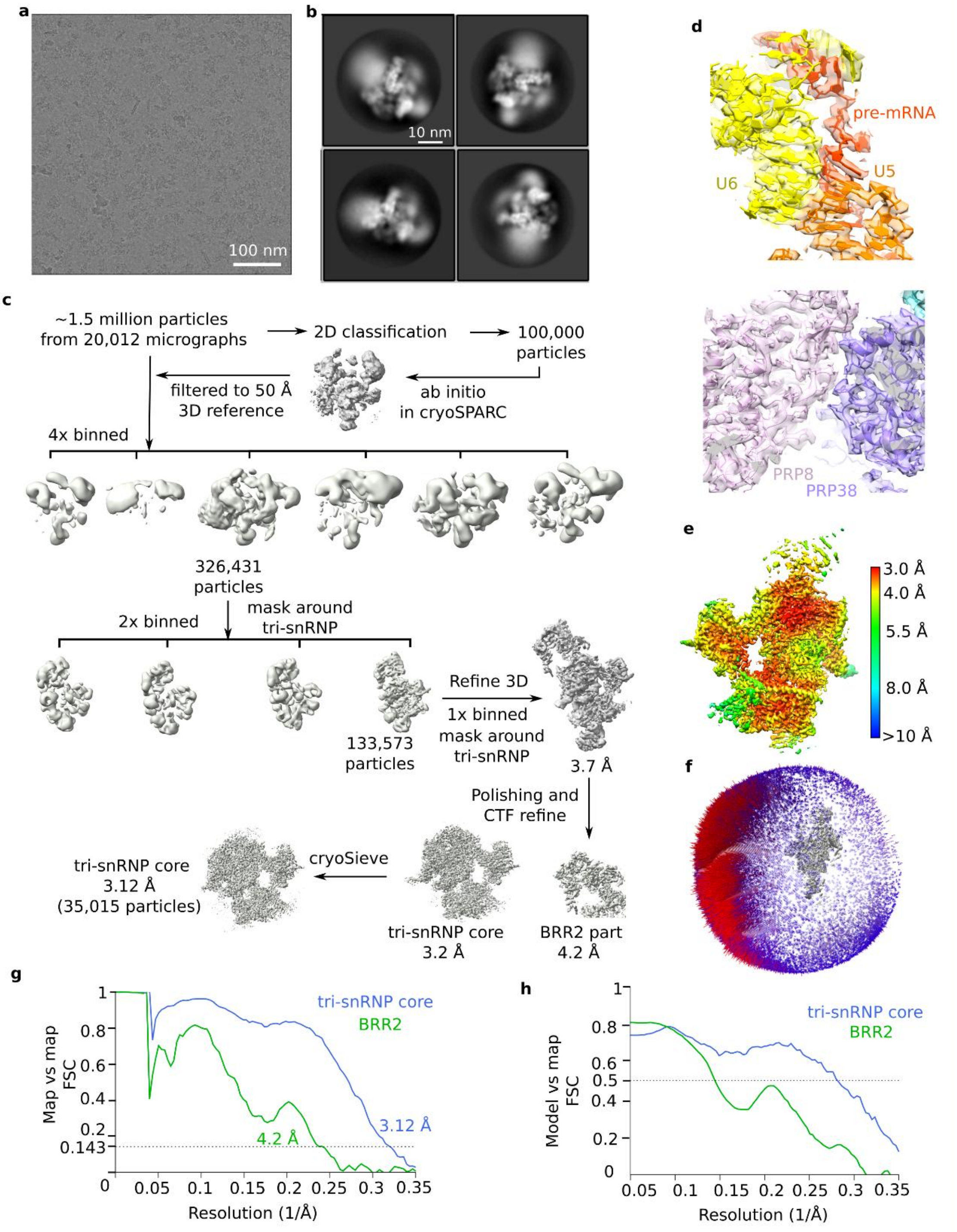

Supplementary Fig. 2 Cryo-EM and image-processing of the OTS964-stalled PM5-10 spliceosomes (PM5-10 pre-B$^{act\text{-}OTS}$).

a, A representative micrograph after motion correction. **b**, Representative cryo-EM 2D class averages of the pre-B$^{act\text{-}OTS}$ complex. Class averages typical of B complexes were absent. **c,** Cryo-EM computation sorting scheme. All major image-processing steps are depicted. For a more detailed explanation, see the EM data-processing section in

Methods. **d,** Representative regions of the EM density fit with a stick model. **e,** Local resolution estimation of the tri-snRNP region of the pre-$B^{act\text{-}OTS}$ complex. **f,** Orientation distribution plot for the particles contributing to the reconstruction of the tri-snRNP core region. Red depicts a higher relative number of particles at a certain angle. **g,** Fourier shell correlation (FSC) values for the pre-$B^{act\text{-}OTS}$ complex revealed a resolution of 3.12 Å for the tri-snRNP core and of 4.2 Å for the BRR2 region. **h,** Map versus model FSC curves for the tri-snRNP core and the BRR2 region.

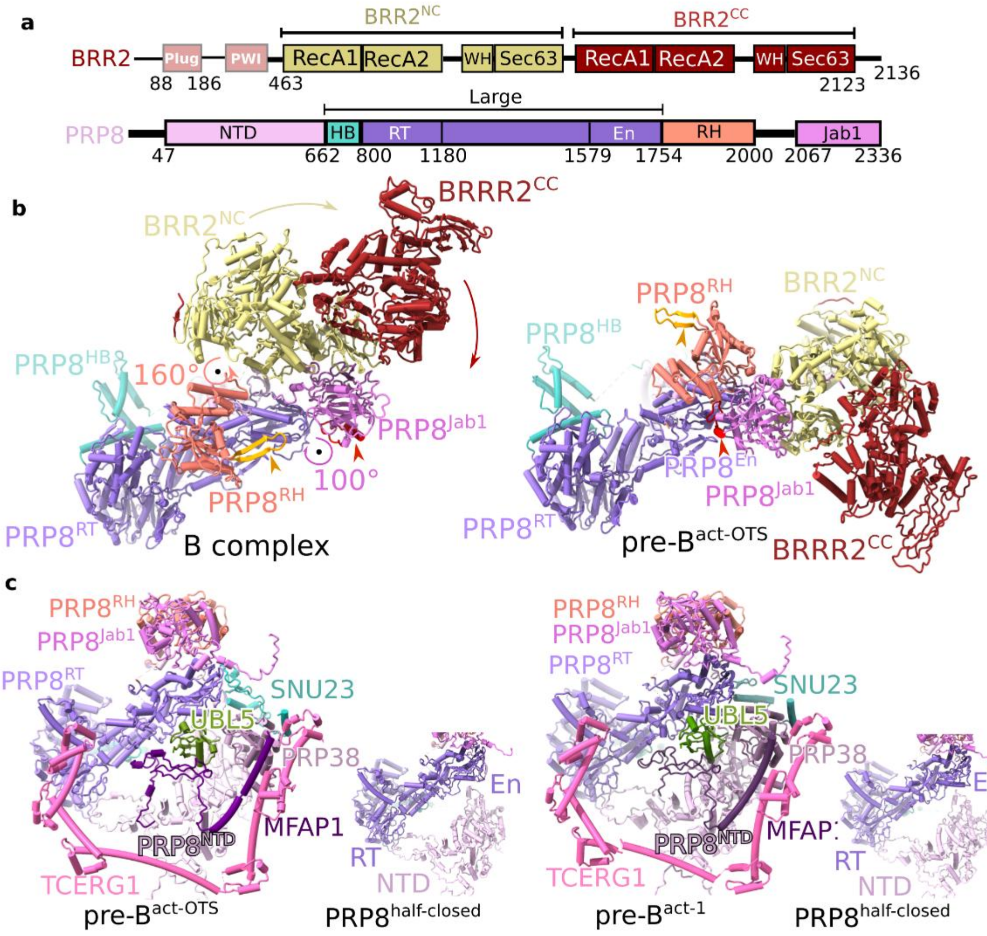

Supplementary Fig. 3 Conformational state of PRP8 and BRR2 in pre-B$^{act\text{-}OTS}$

a, Schematic of the domain organization of BRR2 and PRP8. PWI, domain with a PWI tripeptide located within its N-terminal region; WH, winged helix; NC, the N-terminal helicase cassette; CC, the C-terminal helicase cassette; RT, reverse-transcriptase-like domain; HB, helical bundle; RH, RNase H-like domain; En, endonuclease-like domain; NTD, N-terminal domain; Jab1, Jab1/MPN domain. **b,** Structural rearrangements of the PRP8 and BRR2 region during the transition from B to pre-B$^{act\text{-}OTS}$. The BRR2 helicase, together with its associated PRP8^{Jab1}, is rotated clockwise by 100°. Concomitantly, PRP8^{RH} is rotated counterclockwise by 160°, forming a new contact with PRP8^{Jab1} in the pre-B$^{act\text{-}OTS}$ complex. The PRP8-NTD domain is not shown. Orange arrow, β-hairpin of PRP8^{RH}; red arrow, Jab1$^{2230\text{-}2250}$ **c,** In the pre-B$^{act\text{-}OTS}$ complex, PRP8 adopts the half-closed conformation, in which the B-specific proteins (SNU23, MFAP1, PRP38 and UBL5) are bound to PRP8 in the same manner as in the pre-B$^{act\text{-}1}$ complex[1]. TCERG1 also adopts the same conformation as in the pre-B$^{act\text{-}1}$ complex.

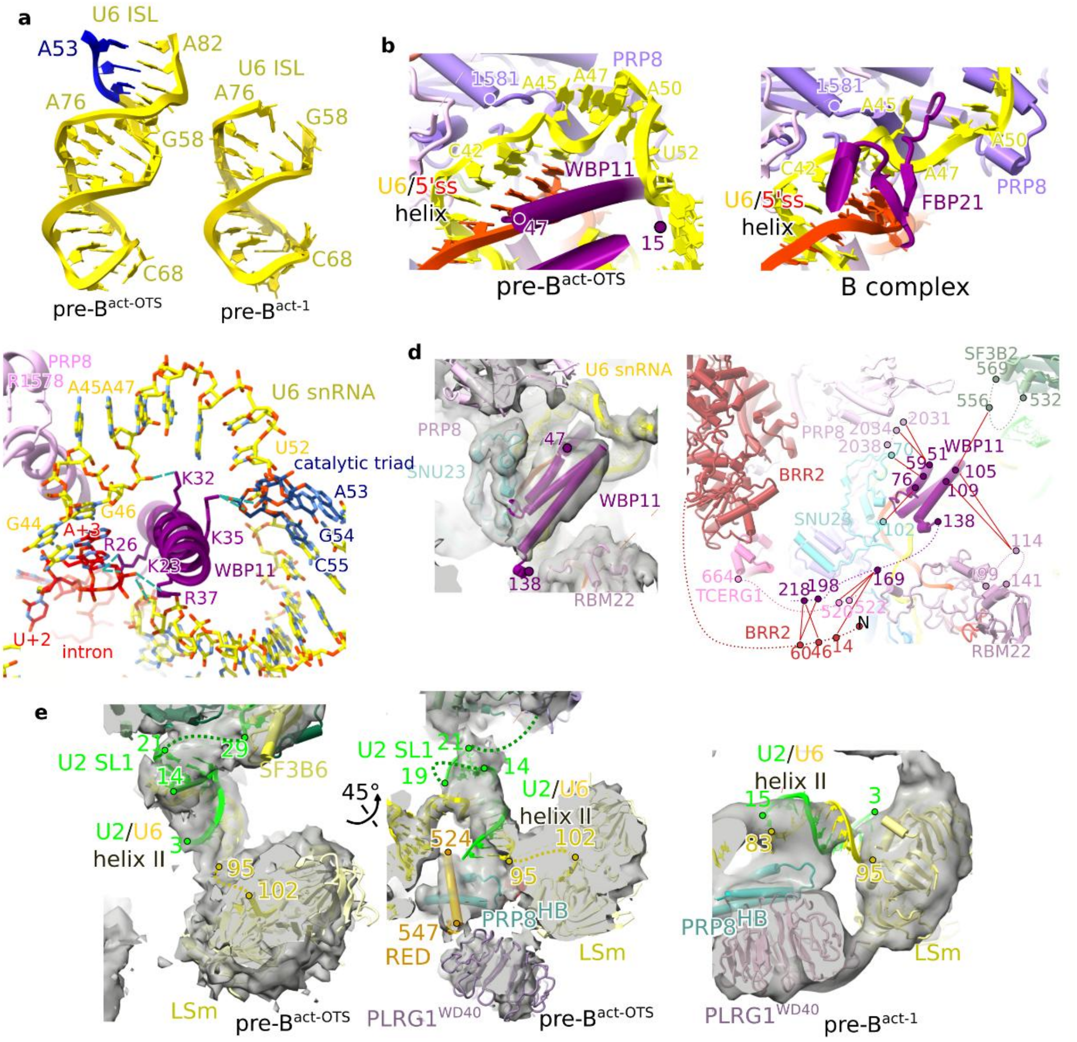

Supplementary Fig. 4 Structural organization of the U2 and U6 snRNAs in pre-B$^{\text{act-OTS}}$

a, Structural comparison of the U6 ISL of the pre-B$^{\text{act-OTS}}$ and the pre-B$^{\text{act-1}}$ complexes. The extended U6 ISL in the pre-B$^{\text{act-OTS}}$ complex contains six additional base-pairings at its base, which includes the U6 catalytic triad A53-G54-C55 (colored in blue). We note that the base-pairings in the upper stem also differ slightly from those in pre-B$^{\text{act1}}$. **b,** In pre-B$^{\text{act-OTS}}$, the N-terminal α helix of the B-specific protein WBP11 is inserted into the recess created by the extended U6/5′ss helix, the U6 ISL and the connecting single-stranded U6 nts A42–U52. Comparison of the pre-B$^{\text{act-OTS}}$ and the B complexes shows that the positions of WBP11 and FBP21 in the pre-B$^{\text{act-OTS}}$ and B complexes, respectively, are mutually exclusive. **c.** Atomic interactions between the long α helix of

WBP11 and U6 snRNA and pre-mRNA. WBP11's α helix runs along the U6/5′ss helix, contacting the phosphate backbone of the 5′-terminal region of the pre-mRNA intron and with the backbone of U6 A47 (K32) and G54-G55 (K35). Dashed blue lines, hydrogen bonds. **d,** AF3 predicts a helical bundle for WBP11 (aa 48–138) directly downstream of its N-terminal α helix, which can be fitted into the EM density near SNU23, guided by our crosslinks. The crosslinks of WBP11 and its neighboring proteins are shown alongside. This helical bundle possibly facilitates the docking of WB11's N-terminal α helix along the extended 5′ss/U6 RNA helix. Crosslinked residues are depicted as colored circles connected by red lines. Dashed, colored lines indicate the potential position of unstructured regions of the proteins. **e,** Fitting of U2/U6 helix II, U2 stem-loop1 (SL1), RED (aa 524–547) and U6 LSm into the EM density. In pre-$B^{act\text{-}OTS}$, the U2/U6 helix II is docked perpendicularly to $PRP8^{HB}$. However, in pre-$B^{act\text{-}1}$, it runs almost parallel to the HB domain. In addition, in pre-$B^{act\text{-}OTS}$, a short α helix of RED (aa 524–547) binds $PRP8^{HB}$ at a site, mutually exclusive with $PLRG1^{WD40}$ in pre-$B^{act\text{-}1}$. This may explain why in pre-$B^{act\text{-}OTS}$, the U6 LSm complex and $PLRG1^{WD40}$ occupy an intermediate position compared to pre-$B^{act\text{-}1}$ (see also Fig. 4c).

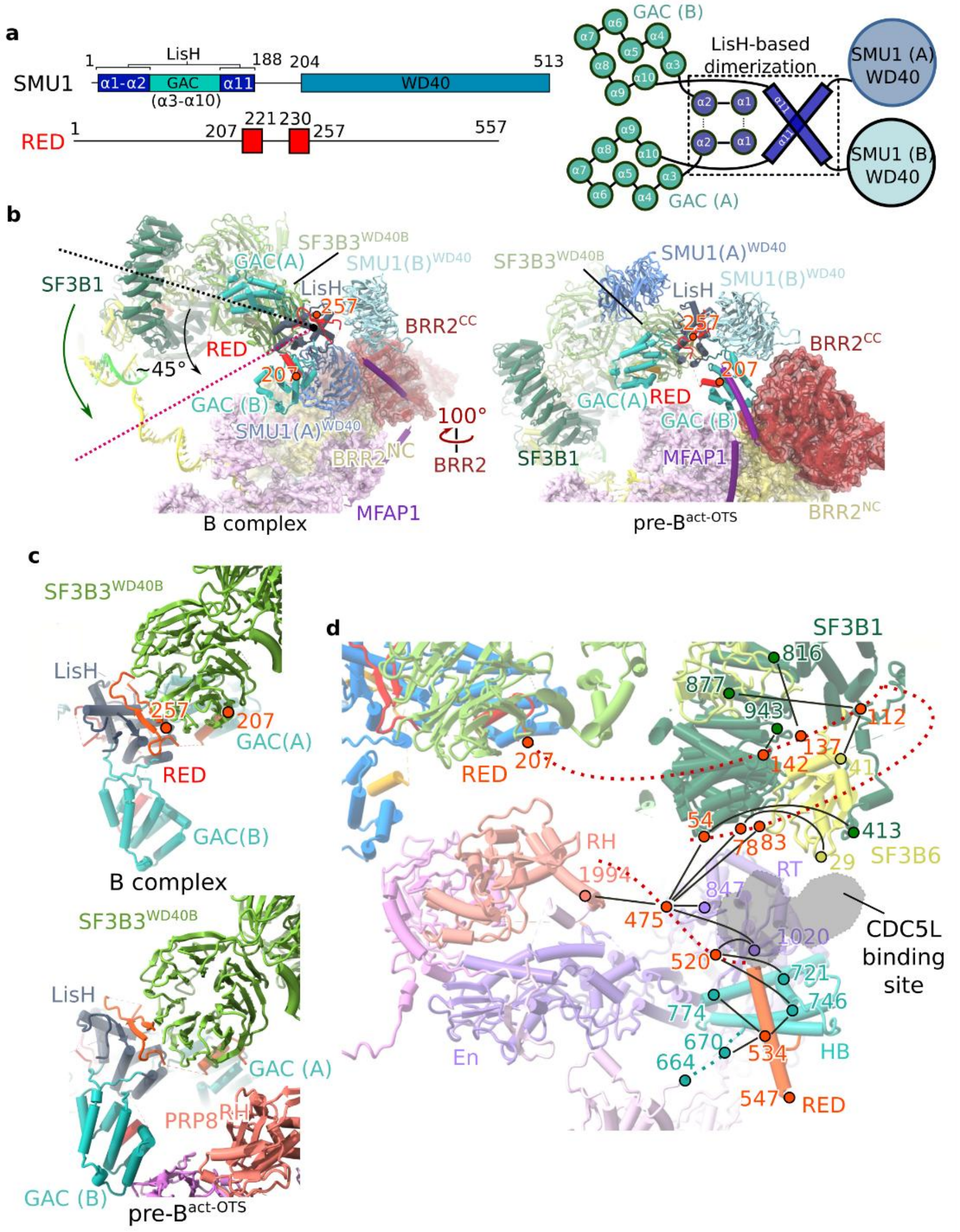

Supplementary Fig. 5 Structural comparison of SMU1/RED in B and pre-B$^{act\text{-}OTS}$ complexes.

a, Schematic of the domain organization of SMU1 and RED, and schematic depiction of the SMU1 dimerization domain; see also Figure 4a. **b,** Coordinated movements of the U2 snRNP 5′ domain, the SMU1-RED tetrameric complex and the BRR2 helicase during the B-to-pre-B$^{act\text{-}OTS}$ transition. In the B complex, the LisH (Lis homology) and GAC^{A} (globular α-helical core A) domains of the SMU1 dimer form a bridge to the U2 snRNP 5′ domain through interactions mediated by residues 207–257 of one RED copy (RED$^{207–257}$). The 5′ domain of the U2 snRNP, together with the SMU1-RED

tetramer, rotates clockwise by ca 45° towards PRP8. Concurrently, BRR2 rotates by ca. 100°. As the interface between U2 SF3B3^{WD40} and SMU1/RED's LisH and GAC(A) domains is maintained during the B to pre B$^{act-OTS}$ transition, the movement of U2 is coupled with the docking of SMU1's GAC(B) domain to BRR2^{CC}, where it replaces the WD40 domain of SMU1(A), leading to a new interaction between SMU1$^{GAC(B)}$ and BRR2. This contact between SMU1$^{GAC(B)}$ and BRR2 probably prevents any further movement of the U2 snRNP (prior to CDK11-mediated phosphorylation of SF3B1^{N}). Thus, among others, SMU1-RED appears to orchestrate the movement of U2 snRNP and BRR2 during the B to pre-B$^{act-OTS}$ transition and stabilize the structure of the latter complex. **c,** RED$^{207-257}$ mediates a stable interaction between the U2 SF3B3^{WD40B} domain and SMU1. **d,** Tentative paths of the C- and N-terminal regions of RED in pre-B$^{act-OTS}$. RED's paths are based on crosslinks and are shown as dashed red-orange lines. Crosslinked amino acid residues are depicted as colored circles, connected by black lines. RED's C-terminal α helix (aa 524–547) binds PRP8^{HB}, and its upstream region extends towards PRP8^{RH}. PRP8 residues K847 and K1020 that crosslink to RED, are at the interface where CDC5L binds. The N-terminal region of RED, upstream of the SMU1-binding region, extends towards the SF3b complex and shows strong crosslinks to both SF3B1 and SF3B6. Its region closer to the N terminus may circle back to RED's C-terminal region, as indicated by the intramolecular crosslinks.

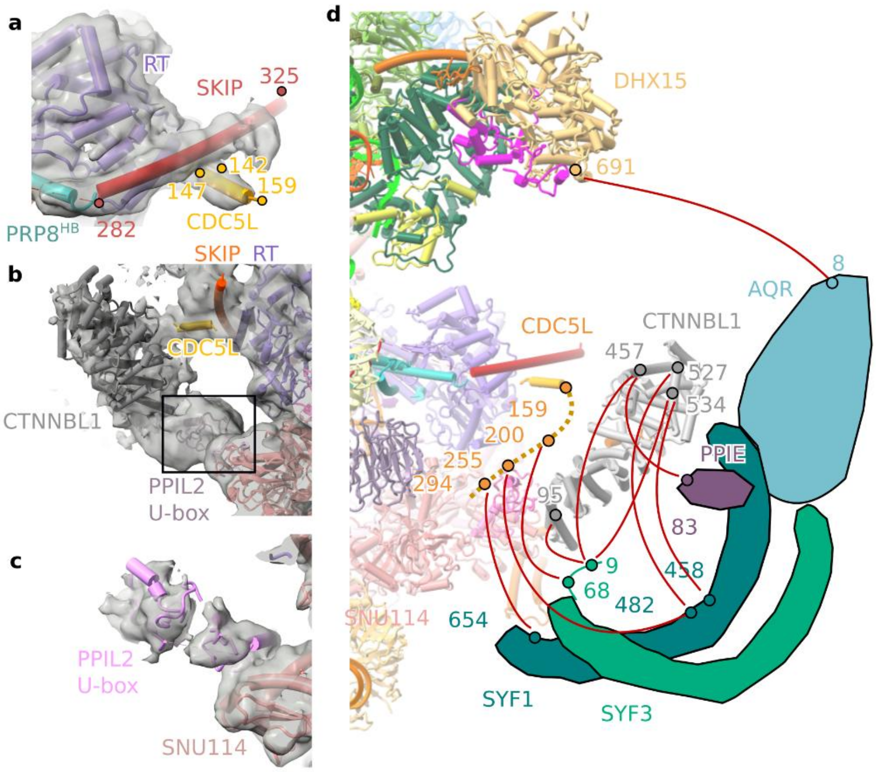

Supplementary Fig. 6 Structural organization of NTC and NTR proteins in pre-B$^{act\text{-}OTS}$ complexes

a, Based on the interactions observed in pre-B$^{act\text{-}2}$, a short α helix of CDC5L can be docked in the EM density near SKIP1. **b,** Based on its position in pre-B$^{act\text{-}21}$, CTNNBL1 can be docked within a flexible density at the corresponding position. **c,** The U-box domain of PPIL2 can be docked within the EM density, connecting SNU114 and CTNNBL1. **d,** Crosslinks between the IBC proteins, including SYF3, and the modeled parts of the pre-B$^{act\text{-}OTS}$ complex suggest that the IBC proteins are loosely associated at the periphery of the complex. Crosslinked residues are depicted as colored circles connected by red lines.

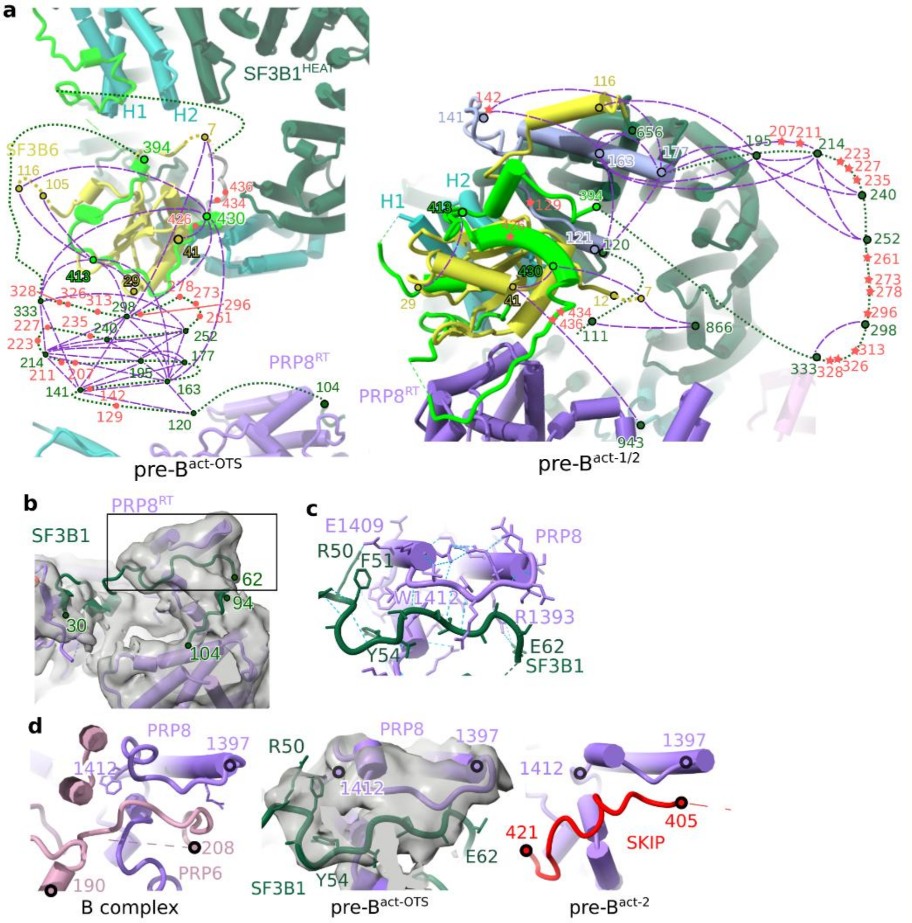

Supplementary Fig. 7 Structural organization of the U2 SF3B1 N-terminal region in pre-B$^{act-OTS}$

a, Intramolecular crosslinks within SF3B1^{N} and between SF3B1^{N} and SF3B6 in pre-B$^{act-OTS}$ and pre-B$^{act-1/2}$. In pre-B$^{act-OTS}$ the SF3B6/SF3B1$^{394-430}$ is associated with HRs 12–14 of SF3B1^{HEAT}. SF3B1^{N} (aa 394–430) that wraps around SF3B6 in the model is shown in light green. The central region of SF3B1^{N} (aa 104–394) is depicted as a virtual, meandering stippled green line located between the SF3B6/SF3B1$^{394-430}$ module and PRP8^{RT}. (The N-terminal region of SF3B1^{N} (aa 30–104) is bound to PRP8^{RT}, see below.) Crosslinked lysine residues within the modeled part of SF3B1 (aa 394–430) are depicted in light green numbers (with black outline). Dark green numbers indicate the virtual position of crosslinked lysine residues within the central region of SF3B1^{N}. Yellow numbers indicate the likely positions of crosslinked lysine residues in SF3B6. Red numbers indicate the serine and threonine residues within SF3B1^{N} that were found to be phosphorylated in purified PM5-10 B^{act} complexes (see also Supplementary Fig.

8a), and which thus become phosphorylated by CDK11 during the transition of pre-B$^{\text{act-OTS}}$ to B^{act}. In contrast, in pre-B$^{\text{act1/2}}$ complexes (pre-B$^{\text{act-2}}$ complex is shown), after CDK11-mediated phosphorylation of SF3B1^{N} (phosphorylated amino acids are shown by red asterisks), significantly fewer crosslinks were identified (taken from Townsend *et al.*,[1]). **b,** AF3-predicted high-confidence interaction between the N-terminal region of SF3B1 (aa 30–104) and PRP8^{RT} residues around 1400–1500, which can be fit into the corresponding density of the pre-B$^{\text{act-OTS}}$ map. **c,** Depiction of AF3-predicted molecular contacts between SF3B1$^{\text{50-62}}$ and PRP8$^{\text{1390-1430}}$. **d,** The PRP8^{RT} region comprising residues 1390 to 1430, sequentially interacts with PRP6$^{\text{190-208}}$ in the B complex[5], with SF3B1$^{\text{50–62}}$ in pre-B$^{\text{act-OTS}}$, and with SKIP$^{\text{405-421}}$ in pre-B$^{\text{act-2}}$[1].

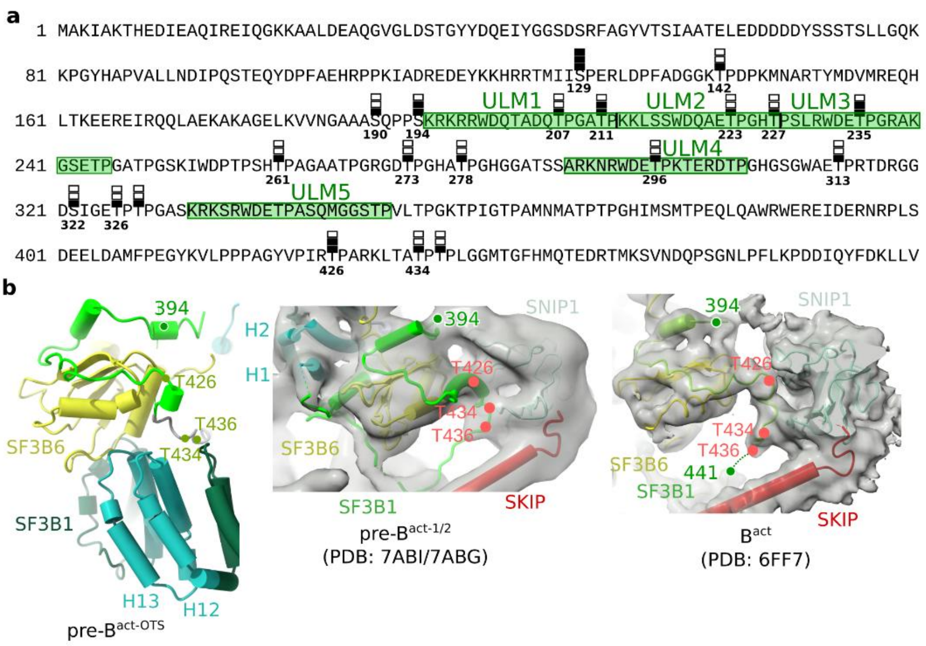

Supplementary Fig 8. Mapping of phosphorylated serine and threonine residues within SF3B1's N-terminal region in B^{act}

a, Phosphorylation pattern of $SF3B1^{N}$ in the PM5-10 B^{act} complex. Phosphor-sites were identified and semi-quantified by MS. The black boxes above the SF3B1 sequence depict the degree of phosphorylation as reported by PEAKS (% modified precursor area of total, schematically one filled box corresponds to 2–30%, two filled boxes to 30–60%, three filled boxes to >60%). ULMs 1–5 [6] are marked with light-green boxes. Except for ULM5, all other ULMs are either flanked or interspersed with phosphor-sites, raising the possibility that CDK11-mediated phosphorylation may also regulate the strength of interaction of UHM proteins, such as U2AF2, with their cognate ULMs. **b**, Potential role of the phosphorylation of threonine residues 426, 434 and 436, in the repositioning of the SF3B6/$SF3B1^{394-430}$ module at $SF3B1^{HEAT}$ during the pre-$B^{act-OTS}$ to B^{act} transition. In pre-$B^{act-OTS}$, the SF3B6/$SF3B1^{394-430}$ module is attached to the HRs 12–14 of $SF3B1^{HEAT}$ with T426, T434 and T436 (yellow-green color; unphosphorylated state) being located close to/at the interface of HR12. We showed previously that in pre-$B^{act-1/2}$, the RES complex protein SNIP1 is also recruited to the spliceosome and interacts with the repositioned SF3B1/$SF3B1^{394-430}$ module[1]. Notably, the T426, T434 and T436 residues are now located at, or near to, the interface with SNIP1; they maintain their position also in the mature B^{act} complex.[7]

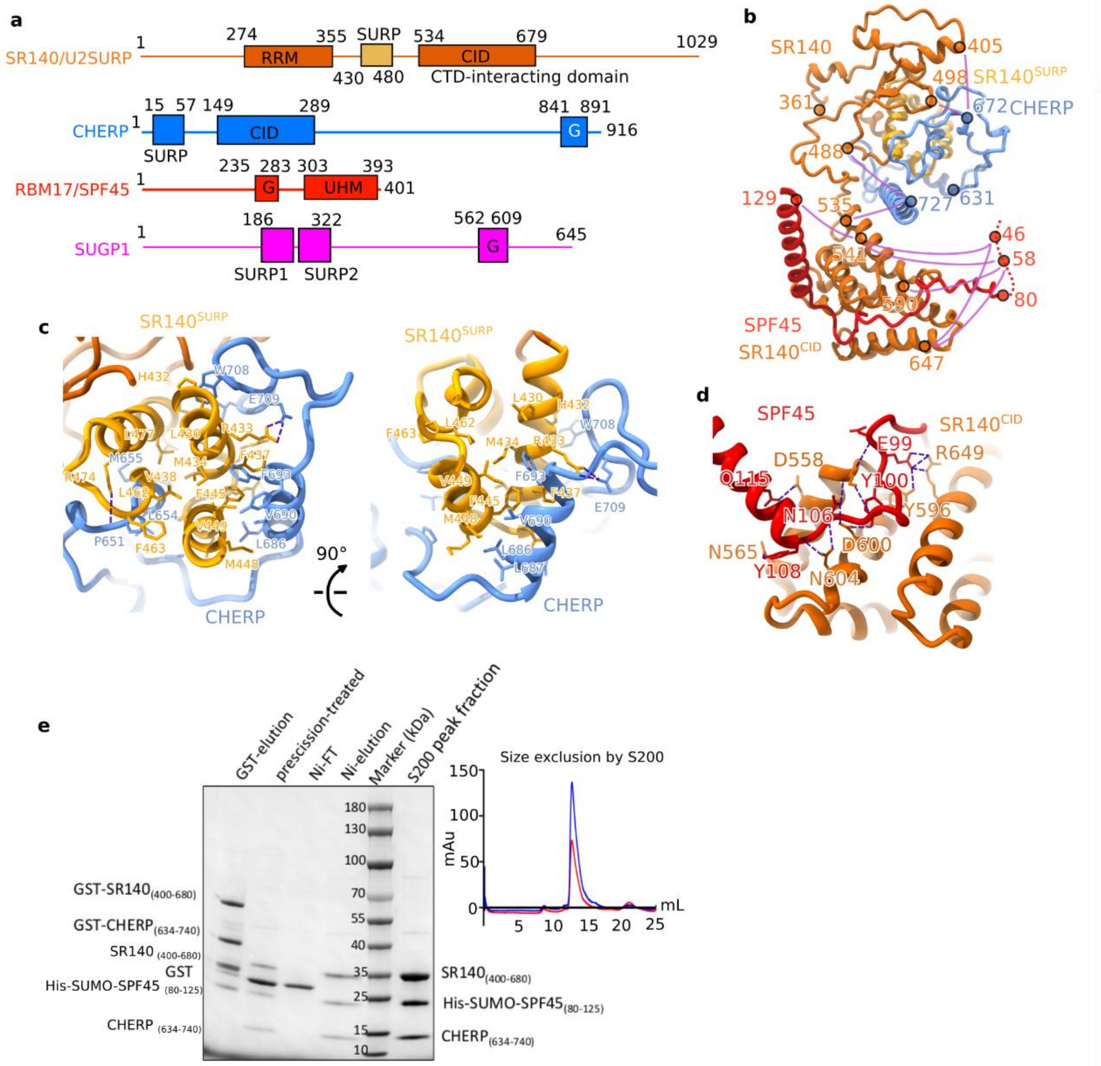

Supplementary Fig. 9 Characterization of interaction sites of the G-patch proteins CHERP and SPF45 at the SR140 protein within pre-B$^{\text{act-OTS}}$

a, Schematic of the domain organization of SR140/U2 SURP, CHERP, SPF45/RBM17, and SUGP1. RRM, RNA-binding motif; CTD, C-terminal domain; UHM, U2AF homology motif; G, G-patch motif, a ~45-residue glycine-rich motif that directly binds and activates DEAH helicases[8-10]. SURP, also known as SWAP (suppressor-of-white-apricot) domain. **b,** Crosslinks between SR140 and G-patch proteins CHERP and SPF45, respectively, as observed within the pre-B$^{\text{act-OTS}}$ complex; consistently with AF3-mediated structure predictions (see **c**). Crosslinked residues are depicted as colored circles connected by pink lines. **c,** AF3-predicted molecular interactions between SR140 and CHERP. CHERP wraps around the SURP domain of SR140, and

the interactions are mediated mainly by hydrophobic interactions. Dashed blue lines represent hydrogen bonds. **d,** AF3-predicted molecular interactions between part of SR140's CID domain and SPF45. **e,** Co-purification of GST-SR140 (aa 400–680), GST-CHERP (aa 634–740) and His-SUMO-SPF45 (aa 80–125). The plasmids encoding the three protein fragments were co-transformed into *Escherichia coli* strain BL2, and protein complexes, formed by the recombinantly expressed proteins, were purified and subsequently separated by SDS-PAGE as follows. Lane 1, protein complex as purified by GST pull-down; lane 2, protein fragments after removal of GST-tag by digestion with PreScission protease; lane 3, the PreScission-treated sample was then subjected to Ni-NTA resin (the flow-through contains only the GST-tag); lane 4, the eluate from the Ni-NTA resin contains the three protein fragments SR140 (aa 400–680), His-SUMO-SPF45 (aa 80–125), and CHERP (aa 634–740); lane 5, marker; lane 6, the eluate from Ni-NTA was subjected to Superdex 200 (S200) size exclusion column, and the peak fraction was visualized, showing that the three protein fragments form a stable complex and can be co-purified after two-step affinity purification followed by a size-exclusion purification. The purification profile by S200 is shown on the right. Blue and red lines indicate UV absorption at 280 nm and 260 nm, respectively. mAu, milli absorbance unit.

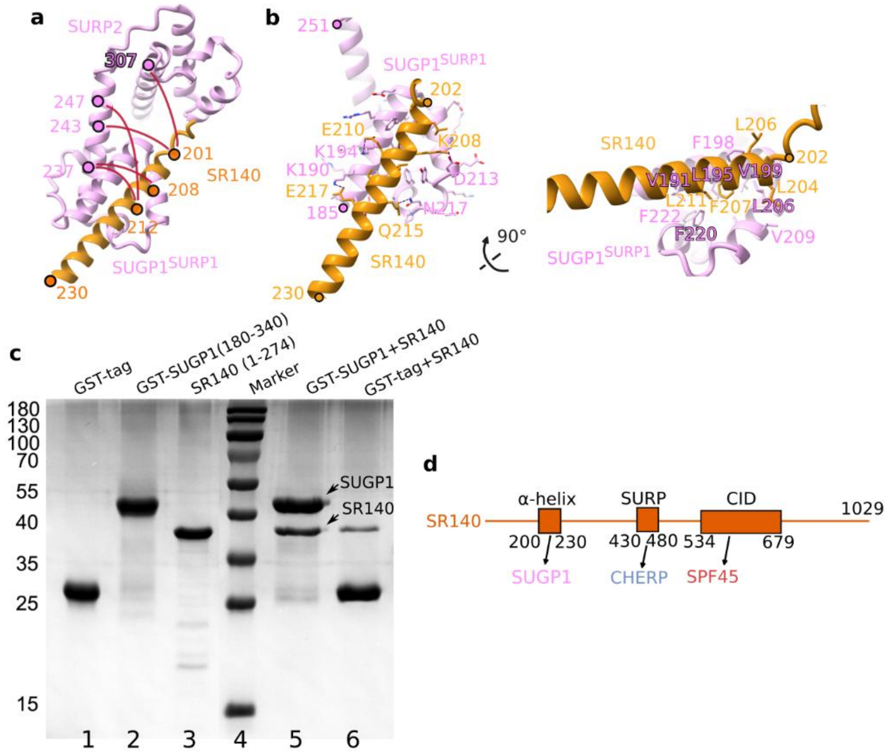

Supplementary Fig. 10 Characterization of the interaction site of the G-patch protein SUGP1 at the SR140 protein within pre-B$^{act\text{-}OTS}$

a, Crosslinks between SUGP1's SURP domains and an α helix of SR140 (aa 201–230). Crosslinked residues are depicted as colored circles connected by red lines. **b,** AF3 further predicts a high-confidence interaction between an α helix of SR140 (aa 200–230), just upstream of the N-terminal side of SR140's RRM domain, and the SURP domains of SUGP1 (SUGP1^{SURP}), which agrees well with our crosslinks, and with the interactions *in vitro* between recombinant proteins (see panel c, below). The two proteins interact mainly through salt bridges and hydrophobic interactions. We note that the predicted molecular interactions between the latter proteins are very similar to those observed by X-ray crystallography of the complex between an α helix of SF1 and the SURP-1 domain of SF3A1[11]. **c,** Purified recombinant GST-tagged SUGP1 fragment (aa 180–340) and an N-terminal SR140 fragment (aa 1–274) were used for pull-down assays. The purified protein fragments are separated by SDS-PAGE. Lanes 1–3 show GST-tag, GST-SUGP1 (aa 180–340) and SR140 (aa 1–274), respectively; lane 4, marker proteins; lane 5, 20 µg of purified GST-SUGP1 (aa 180–340) and 70 µg of

SR140 (aa 1–274) fragments were mixed and subjected to GST-affinity purification, indicating a direct interaction between the two protein fragments; lane 6, as a control, 20 μg of GST protein and 7 μg of SR140 (aa 1–274) fragments were mixed and subjected to GST-affinity purification, indicating that GST alone exhibits only background binding to SR140 (aa 1–274). **d,** SR140 interacts with SUGP1, CHERP and SPF45 through distinct, non-overlapping regions.

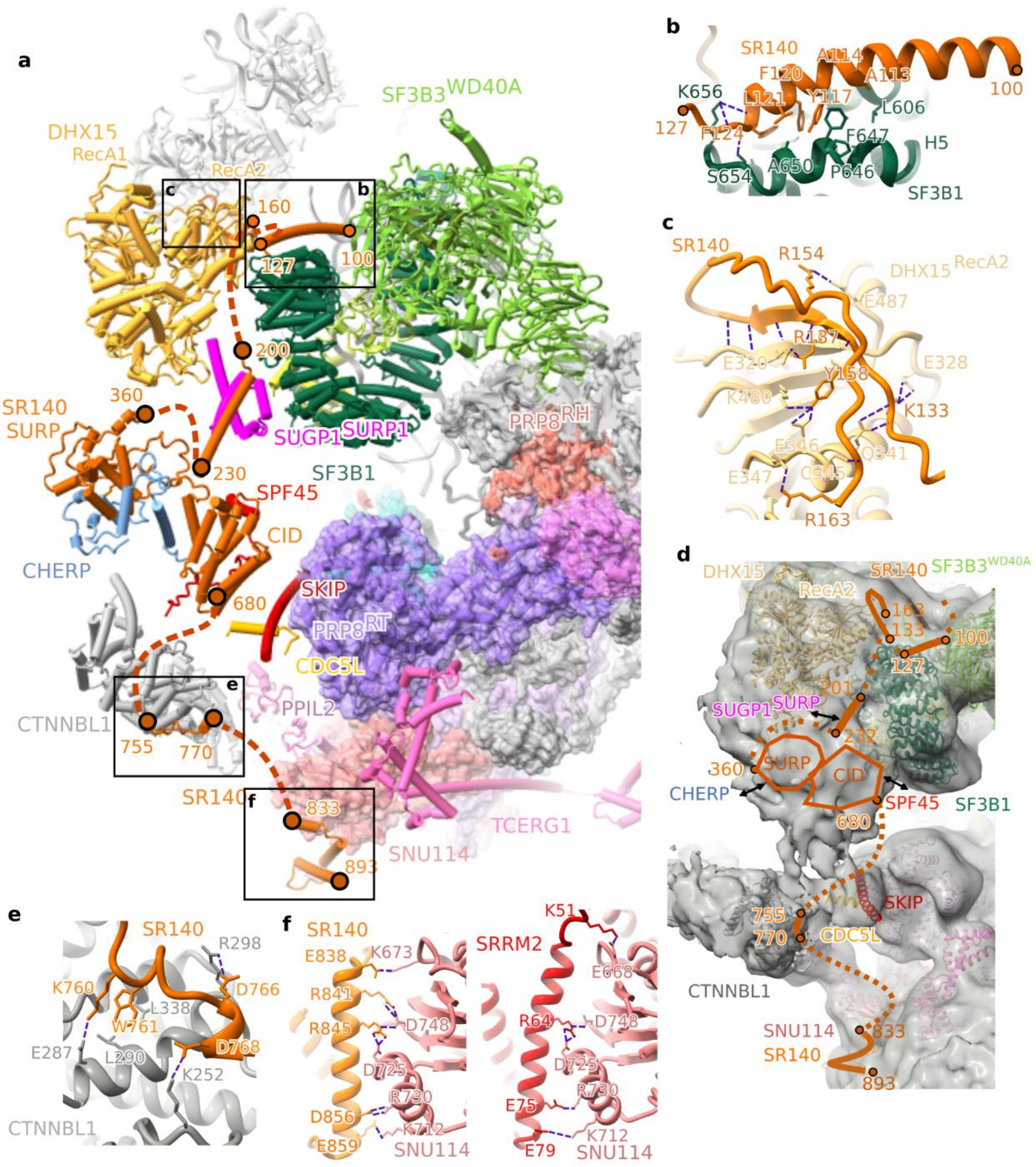

Supplementary Fig. 11 Recognition of peripheral surfaces of pre-B$^{act\text{-}OTS}$ by SR140 and SR140's targeting of G-patch proteins to the spliceosome

a, Overview of the path of SR140 along the surface of the pre-B$^{act\text{-}OTS}$ spliceosome. SR140 tethers DHX15 and SF3B1 through its N-terminal region. Its middle region interacts with the G-patch proteins SUGP1, CHERP and SPF45 (see also Supplementary Fig. 9, 10). The C-terminal region extends toward CTNNBL1 and finally reaches SNU114. Because the intron part of PM5-10 downstream of the BS/U2 helix is too short to extend beyond the inner circle of the HEAT domain, DHX15 cannot bind to intron RNA in PM5-10 pre-B$^{act\text{-}OTS}$. Frames, labelled b-f, indicate regions of

PM5-10 pre-$B^{act\text{-}OTS}$, the structures of which are shown in more detail in b–f. **b,** AF3-predicted molecular interactions between an N-terminal α helix of SR140 (aa 100–127) and HR5 of $SF3B1^{HEAT}$. Dashed blue lines, hydrogen bonds. **c,** AF3-predicted molecular interactions between an N-terminal region of SR140 (aa 133–163) and DHX15. **d,** A flexible density bridge, potentially containing the globular domains of the complex between SR140 and the G-patch proteins CHERP, SPF45 and SUGP1 (as mapped in Supplementary Fig. 9, 10), connects the SF3B1/DHX15 complex and the region comprising CTNNBL1, SKIP and CDC5L. The map is low-pass filtered to 30 Å. **e,** AF3-predicted molecular interactions between the HEAT domain of CTNNBL1 and the region of SR140 comprising aa 755–770; consistent with our crosslinks (see Fig 7c). **f,** AF3-predicted molecular interactions between a C-terminal region of SR140 (aa 838–859) and domain IV of SNU114 in pre-$B^{act\text{-}OTS}$. In B^{act} complexes, a region of SRRM2 (aa 51–100) binds at the domain IV of SNU114 through very similar interactions as SR140 in pre-$B^{act\text{-}OTS}$.

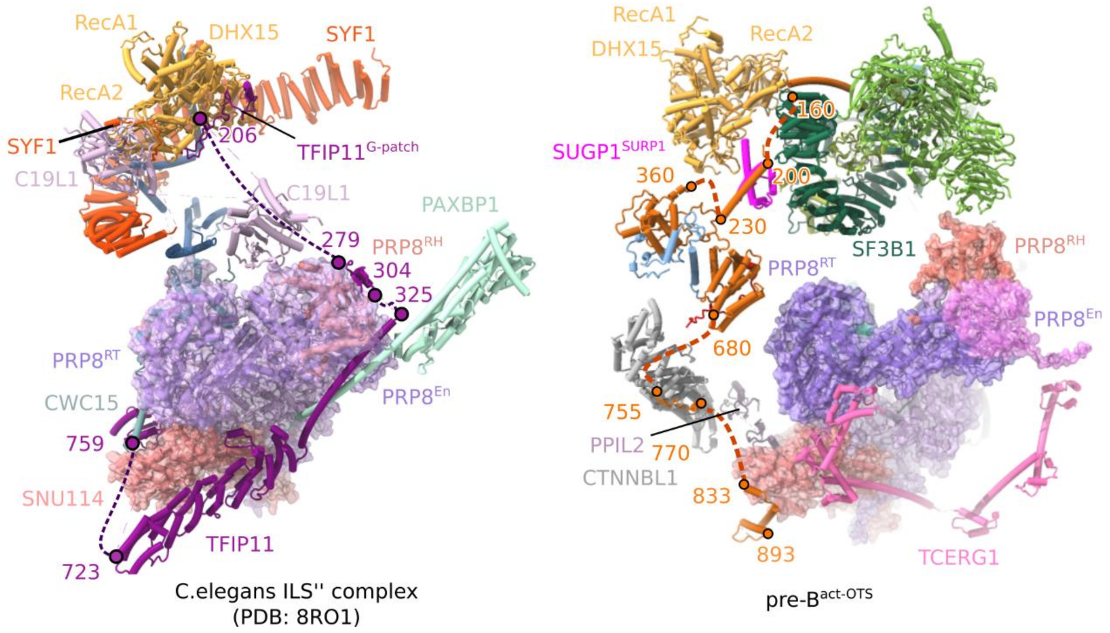

Supplementary Fig. 12 Comparison of the extended, but significantly different, paths of SR140 at the human pre-B$^{act\text{-}OTS}$ complex with that of TFIP11/PAXBP1 at the C. elegans ILS″ complex.

In the C. elegans ILS″ complex, TFIP11 and PAXBP1 bind to the peripheral of the spliceosome, and the G-patch region of TFIP11 activates DHX15 for spliceosome disassembly[12]. The RecA2 domain of DHX15 packs against a C-terminal region of SYF1, which together with TFIP11, orients DHX15 to engage its U6 snRNA target, priming it to dissociate the U6/U2 catalytic core RNP[12]. In the pre-B$^{act\text{-}OTS}$ complex, SR140 also binds to the outer surface of the spliceosome, but takes a significantly different path. Unlike TFIP11, SR140 does not contain a G-patch region, but it is associated with several G-patch-containing proteins, which are probably recruited as a pre-formed complex[13,14], consistent with our crosslinks between those G-patch proteins and SR140 in pre-B$^{act\text{-}OTS}$ (see Supplementary Figs. 9, 10). Similarly to SYF1 in C. elegans ILS″ [12], the N-terminal region of SR140 packs against DHX15's RecA2 domain, likely ensuring its correct orientation. However, in pre-B$^{act\text{-}OTS}$, DHX15 is positioned adjacent to the intron exit site near SF3B1 HRs 5–7 (see also Fig. 6f). From this location, it is probably poised to bind the intron near the 3′ splice site/exon junction, dismantling a remodeled aberrant spliceosome. In contrast, in the ILS complex, DHX15 has accommodated the 3′ end of U6 snRNA in its RNA-binding channel[12], poised to

dissociate the U6/U2 catalytic core RNP. For clarity, only the structural elements of the C. elegans ILS'' and pre-$B^{act-OTS}$ complexes are shown, which are relevant to the discussion here.

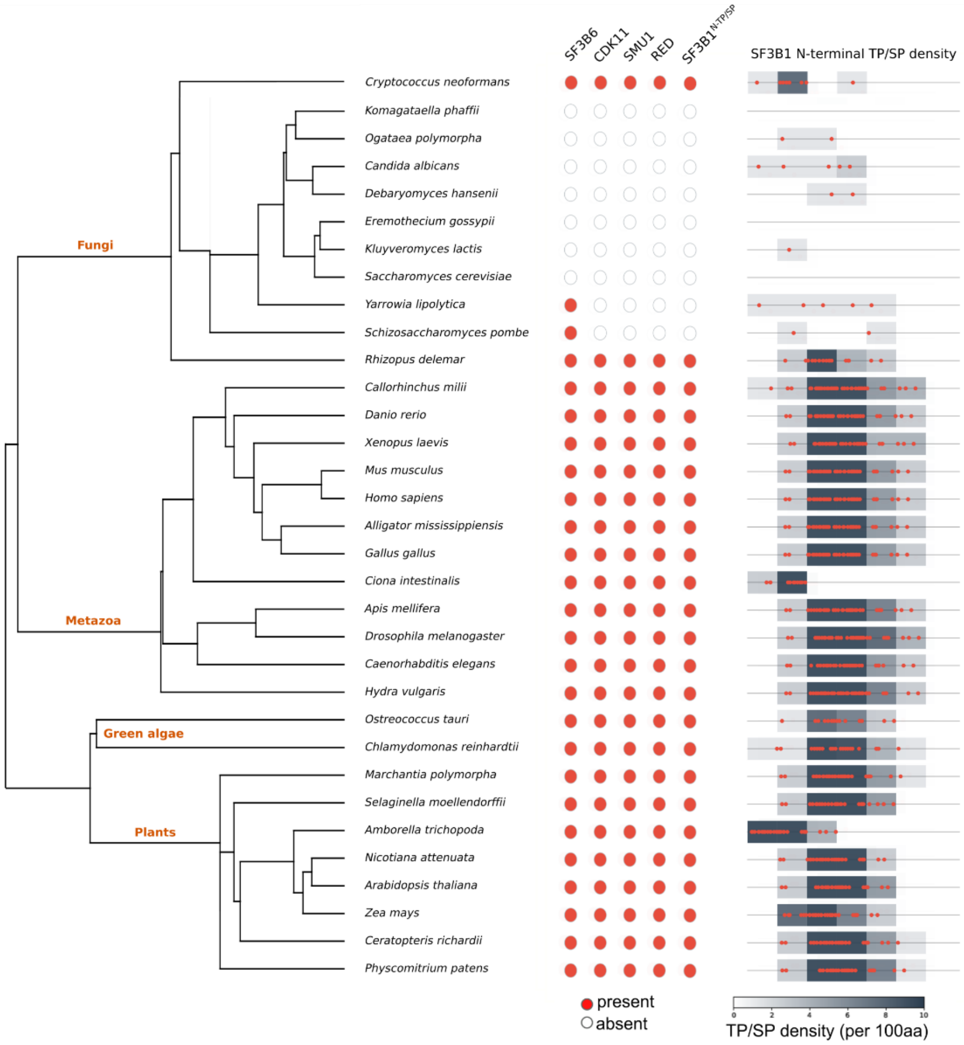

Supplementary Fig. 13. Co-evolution of the SF3B6-CDK11-SF3B1$^{N\text{-}TP/SP}$ axis with SMU1/RED

Phylogenetic tree showing the evolutionary divergence of selected species across the major eukaryotic kingdoms. Distribution of SF3B6, CDK11, a TP/SP dipeptide-rich SF3B1 N-terminal region (SF3B1$^{N\text{-}TP/SP}$), and the B complex proteins SMU1/RED are shown in the central column. Red circles indicate detected homologs, and empty circles indicate their absence. The data reveal a tight evolutionary coupling of these factors across metazoan and plant lineages. Although a putative CDK11 homolog can be identified in *S. pombe*, it lacks the critical N-terminal region that is conserved in metazoans and plants, and CDK11's function is reportedly unrelated to splicing[15]. Notably, *C. neoformans*, which is intron-rich and, compared with *S. cerevisiae*, contains more degenerate splicing signatures [16], stands out as a unique fungal exception

possessing SF3B6, a putative CDK11 homolog, an SF3B1 N-terminal region with numerous TP/SP dipeptides and SMU1/RED proteins. TP/SP dipeptide repeat density in the SF3B1 N-terminus is quantified, and the density of TP/SP motifs per 100 amino acids (aa) is indicated by the gray-scale gradient. Red data points represent individual TP/SP motifs that occurs within the SF3B1 N-terminal region. Note that species lacking the SF3B6-CDK11-SMU1-RED axis generally exhibit significantly reduced or absent TP/SP clusters within the SF3B1 N-terminus.

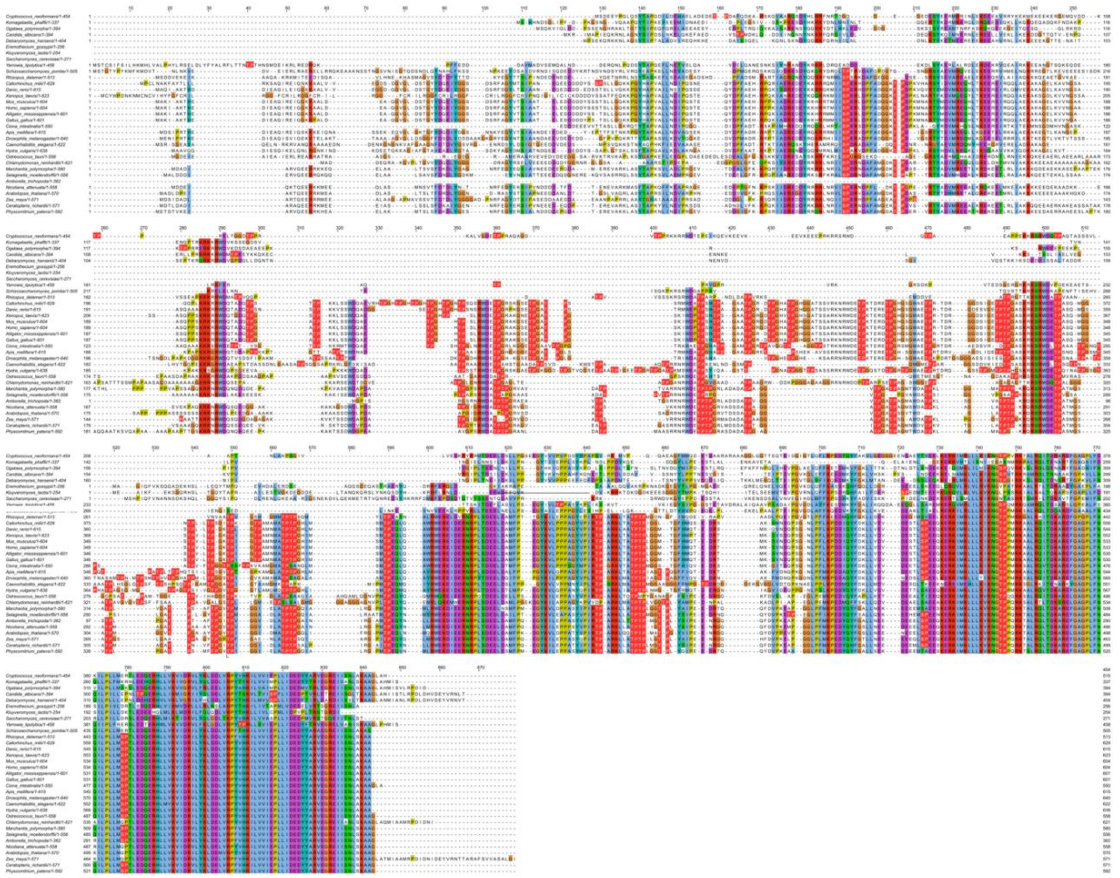

Supplementary Fig. 14. Evolutionary conservation of the SF3B1 N-terminal region.

Multiple sequence alignment of the N-terminal region of the SF3B1 protein across diverse species. Residues are colored according to chemical properties. TP/SP sites are highlighted by light-red background.

Cryo-EM data collection, refinement and validation statistics

	pre-B$^{act-OTS}$ (EMDB-53843) (PDB 9R8V)	pre-B$^{act-OTS}$ core (EMDB-53554) (PDB 9R3D)
**Data collection and processing**		
Magnification	103,700	103, 700
Voltage (kV)	300	300
Electron exposure (e–/Å^2)	39	39
Defocus range (μm)	1-2.5	1-2.5
Pixel size (Å)	1.35	1.35
Symmetry imposed	C1	C1
Initial particle images (no.)	1.5 million	1.5 million
Final particle images (no.)	133,575	35,015
Map resolution (Å)	5.9	3.12
FSC threshold	0.143	0.143
Map resolution range (Å)	5.4-30	2.9-4.5
**Refinement**		
Initial model used (PDB code)	7ABI	7ABI
Model resolution (Å)	--	3.4
FSC threshold		0.5
Model resolution range (Å)	--	3.2-4
Map sharpening *B* factor (Å^2)	--	-60
Model composition	--	
Non-hydrogen atoms		41,826
Protein residues		5,145
Ligands		0
B factors (Å^2)	--	
Protein		85.11
Ligand		184.48
R.m.s. deviations	--	
Bond lengths (Å)		0.002
Bond angles (°)		0.486
Validation	--	
MolProbity score		1.61
Clashscore		9.30
Poor rotamers (%)		0
Ramachandran plot	--	
Favored (%)		97.43
Allowed (%)		2.57
Disallowed (%)		0

Supplementary Table 1. Cryo-EM data collection, refinement and validation statistics

References:

1 Townsend, C. *et al.* Mechanism of protein-guided folding of the active site U2/U6 RNA during spliceosome activation. *Science* **370** (2020). https://doi.org/10.1126/science.abc3753

2 Loyer, P. *et al.* Characterization of cyclin L1 and L2 interactions with CDK11 and splicing factors: influence of cyclin L isoforms on splice site selection. *J Biol Chem* **283**, 7721-7732 (2008). https://doi.org/10.1074/jbc.M708188200

3 Roscigno, R. F. & Garcia-Blanco, M. A. SR proteins escort the U4/U6.U5 tri-snRNP to the spliceosome. *RNA* **1**, 692-706 (1995).

4 Wang, C. *et al.* CDK11 requires a critical activator SAP30BP to regulate pre-mRNA splicing. *EMBO J* **42**, e114051 (2023). https://doi.org/10.15252/embj.2023114051

5 Zhang, Z. *et al.* Cryo-EM analyses of dimerized spliceosomes provide new insights into the functions of B complex proteins. *EMBO J* **43**, 1065-1088 (2024). https://doi.org/10.1038/s44318-024-00052-1

6 Loerch, S. *et al.* The pre-mRNA splicing and transcription factor Tat-SF1 is a functional partner of the spliceosome SF3b1 subunit via a U2AF homology motif interface. *J Biol Chem* **294**, 2892-2902 (2019). https://doi.org/10.1074/jbc.RA118.006764

7 Haselbach, D. *et al.* Structure and Conformational Dynamics of the Human Spliceosomal B(act) Complex. *Cell* **172**, 454-464.e411 (2018). https://doi.org/10.1016/j.cell.2018.01.010

8 Aravind, L. & Koonin, E. V. G-patch: a new conserved domain in eukaryotic RNA-processing proteins and type D retroviral polyproteins. *Trends Biochem Sci* **24**, 342-344 (1999). https://doi.org/10.1016/s0968-0004(99)01437-1

9 Bohnsack, K. E., Ficner, R., Bohnsack, M. T. & Jonas, S. Regulation of DEAH-box RNA helicases by G-patch proteins. *Biol Chem* **402**, 561-579 (2021). https://doi.org/10.1515/hsz-2020-0338

10 Studer, M. K., Ivanović, L., Weber, M. E., Marti, S. & Jonas, S. Structural basis for DEAH-helicase activation by G-patch proteins. *Proc Natl Acad Sci U S A* **117**, 7159-7170 (2020). https://doi.org/10.1073/pnas.1913880117

11 Nameki, N. *et al.* Structural basis for the interaction between the first SURP domain of the SF3A1 subunit in U2 snRNP and the human splicing factor SF1. *Protein Sci* **31**, e4437 (2022). https://doi.org/10.1002/pro.4437

12 Vorländer, M. K. *et al.* Mechanism for the initiation of spliceosome disassembly. *Nature* **632**, 443-450 (2024). https://doi.org/10.1038/s41586-024-07741-1

13 Martín, E., Vivori, C., Rogalska, M., Herrero-Vicente, J. & Valcárcel, J. Alternative splicing regulation of cell-cycle genes by SPF45/SR140/CHERP complex controls cell proliferation. *RNA* **27**, 1557-1576 (2021). https://doi.org/10.1261/rna.078935.121

14 De Maio, A. *et al.* RBM17 Interacts with U2SURP and CHERP to Regulate Expression and Splicing of RNA-Processing Proteins. *Cell Rep* **25**, 726-736.e727 (2018). https://doi.org/10.1016/j.celrep.2018.09.041

15 Drogat, J. *et al.* Cdk11-cyclinL controls the assembly of the RNA polymerase II mediator complex. *Cell Rep* **2**, 1068-1076 (2012). https://doi.org/10.1016/j.celrep.2012.09.027

16 Sales-Lee, J. *et al.* Coupling of spliceosome complexity to intron diversity. *Curr Biol* **31**, 4898-4910.e4894 (2021). https://doi.org/10.1016/j.cub.2021.09.004
