# Supplementary material for: Structural basis of the regulation by CDK11 kinase of early spliceosome activation and evidence for its proofreading by DHX15 helicase
Source: Nat Commun. 2026 Jul 3;17:5845. doi: 10.1038/s41467-026-75109-2 (PMC13332222; doi:10.1038/s41467-026-75109-2)
Supplement: Supplementary file 2 — Description of Additional Supplementary Files [file 41467_2026_75109_MOESM2_ESM.pdf]

Description of Additional supplementary files

Supplementary Data 1: Protein composition of the pre-Bact-OTS complex.

Supplementary Data 2: Protein–protein crosslinks identified in the pre-Bact-OTS complex.

Supplementary Data 3: Summary of modelled proteins and RNAs in the pre-Bact-OTS complex.

Supplementary Movie 1: Structural remodeling from B to pre-Bact-OTS complex After BRR2-mediated removal of U4 snRNA and the associated proteins, the now single-stranded U6 snRNA folds into the extended ISL conformation, docking to PRP8NTD. The folding of U6 snRNA drives the large-scale translocation of the U2 snRNP. Concomitantly, the proteins hNTR and hNTC are recruited.

Supplementary Movie 2: Remodeling of the interface between U2 snRNP and BRR2 during the B-topre-Bact-OTS transition The interface between U2 SF3B3WD40 and SMU1/RED is largely maintained during the B-to-pre-B act-OTS transition. The movement of U2 is coordinated with the rotation of BRR2 by the SMU1/RED complex, and coupled with the docking of SMU1GAC(B) domain to BRR2CC , where it replaces SMU1WD40(A), forming a new interaction between SMU1GAC(B) and BRR2.

Supplementary Movie 3: Coordinated movements of U2 snRNP and BRR2 The 5' domain of the U2 snRNP, together with the SMU1-RED tetramer, rotates clockwise by ca. 45° towards PRP8. Concurrently, BRR2 rotates by ca. 100°.

Supplementary Movie 4: Structural remodeling from the pre-Bact-OTS to the pre-Bact-1 complex During the pre-B act-OTS - to-pre-B act-1 transition, the 5' domain of U2 snRNP undergoes a translocation and docks to PRP8RH. The translocation of SF3B6 from HRs 12–14 to HRs 1–2 is coordinated with the docking of the U2 SF3b complex to PRP8RH. In pre-B act-1 , RES proteins (including SNIP1) are recruited, making contact with the translocated SF3B6, and they further stabilize the new position of the latter.

Supplementary Movie 5: Translocation of SF3B6 as a prerequisite for the docking of SF3b to PRP8RH The translocation of SF3B6 is required for docking of the U2 SF3b complex to PRP8RH. If SF3B6 were to remain bound to HRs 12–14, it would clash with PRP8RT and block the translocation of the U2 snRNP.
