# Supplementary figures and images for: Structural basis of the regulation by CDK11 kinase of early spliceosome activation and evidence for its proofreading by DHX15 helicase
Source: Nat Commun. 2026 Jul 3;17:5845. doi: 10.1038/s41467-026-75109-2 (PMC13332222; doi:10.1038/s41467-026-75109-2)

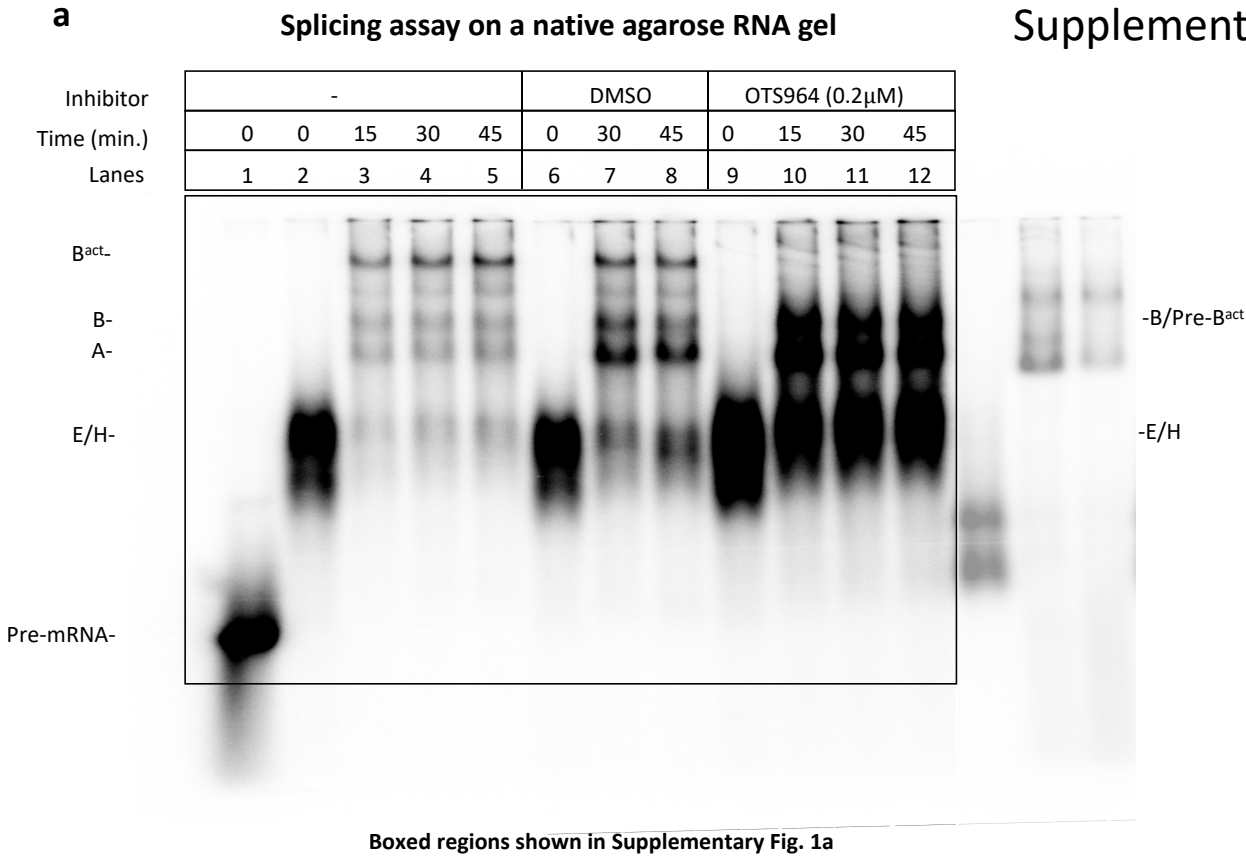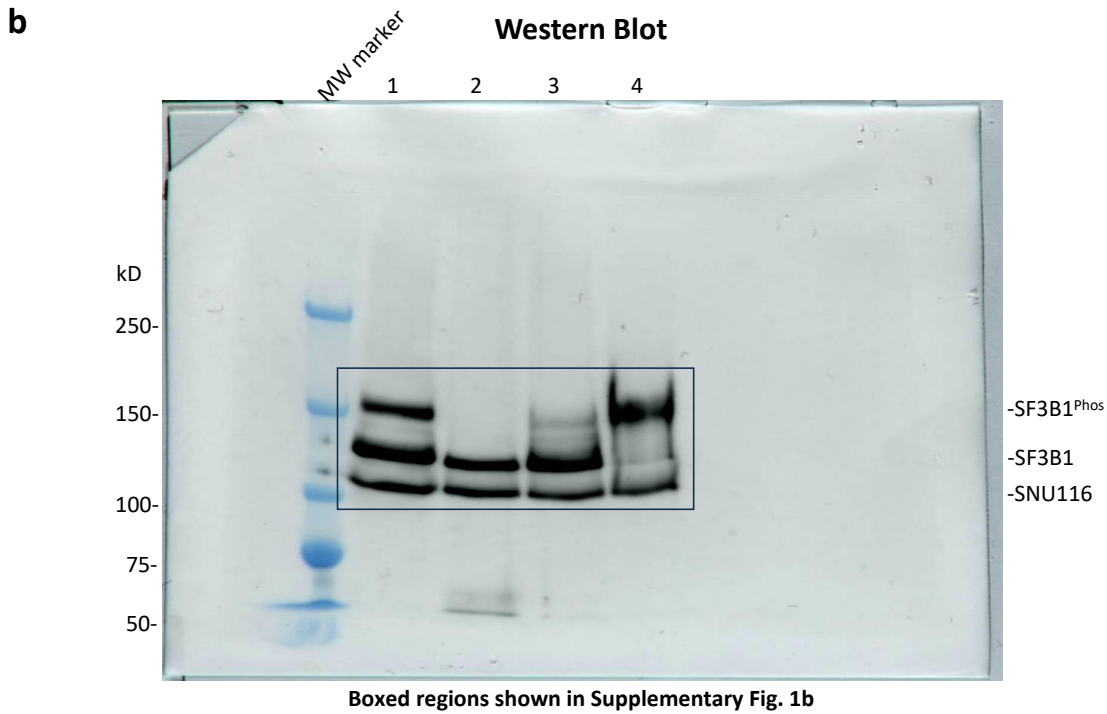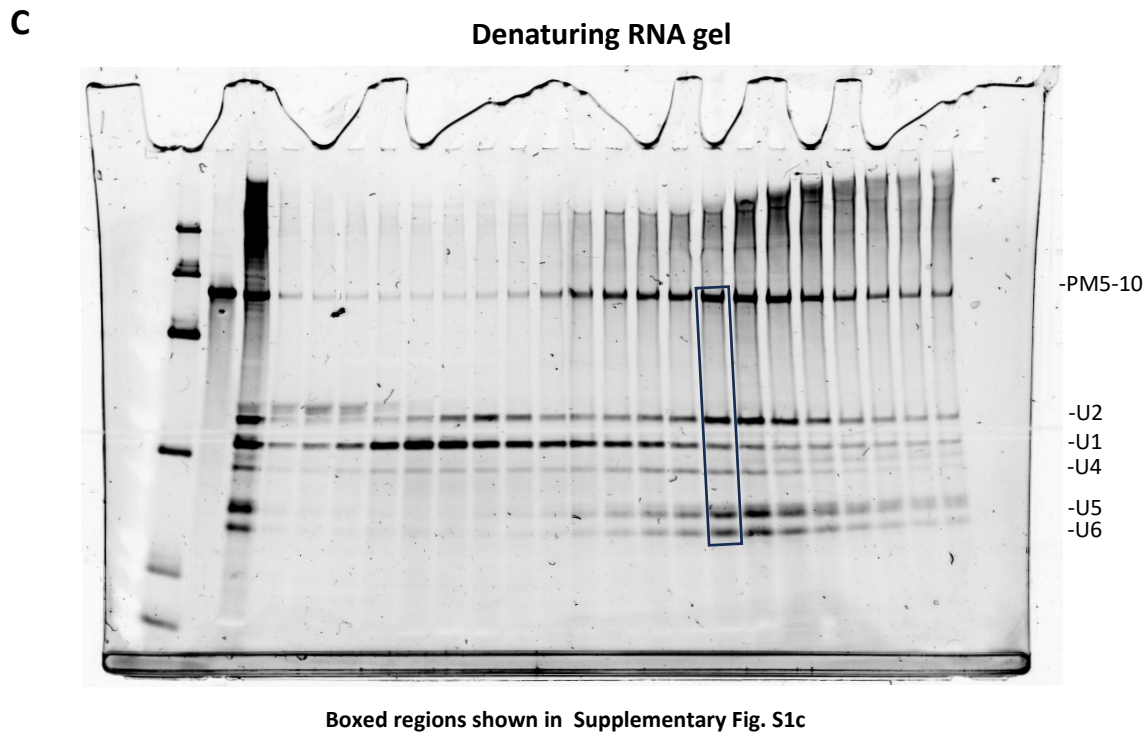

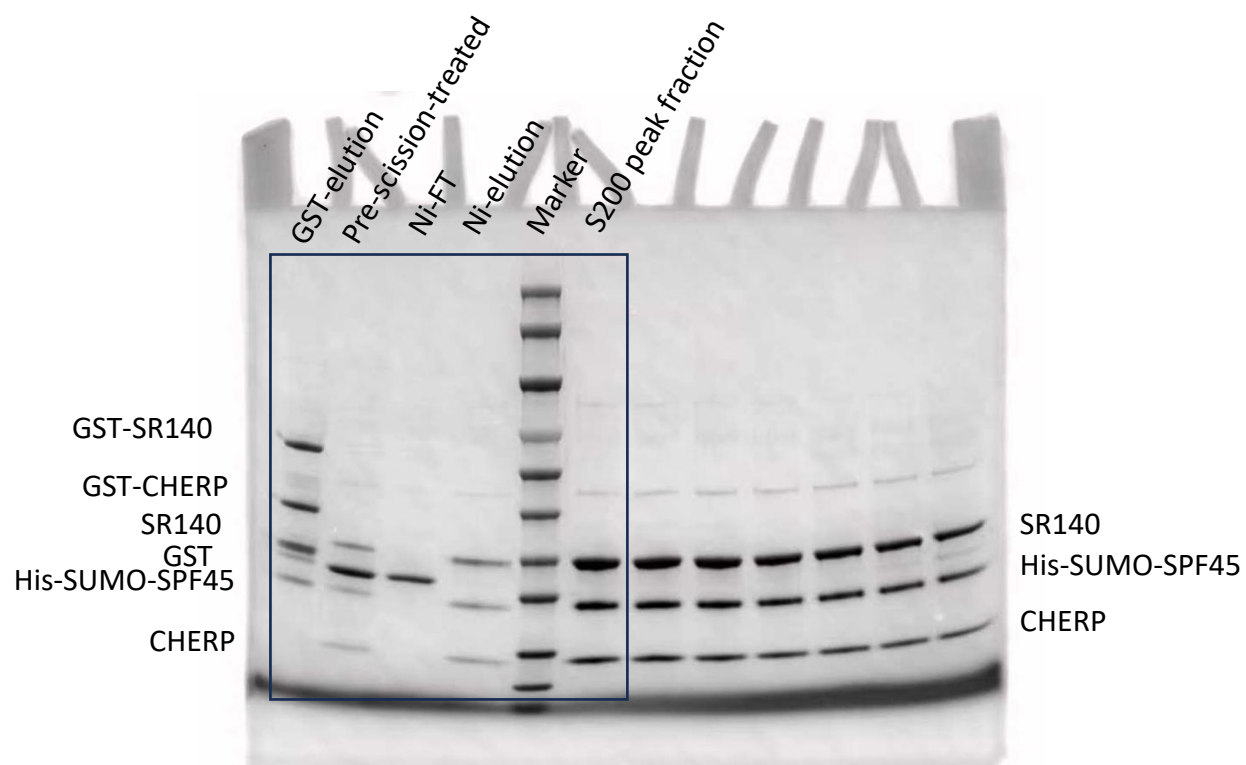

Boxed regions shown in Supplementary Fig. 9e

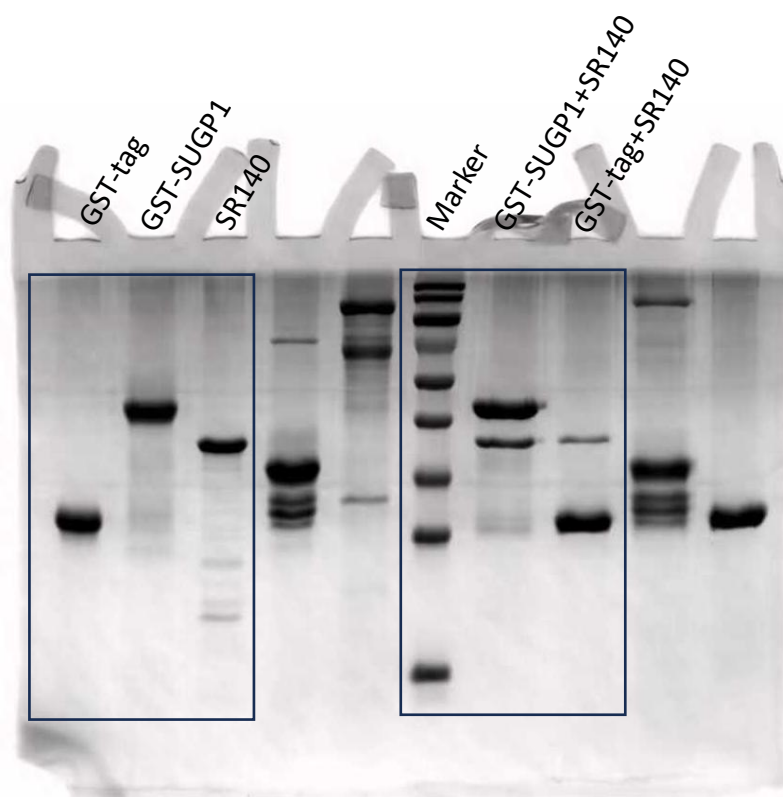

Boxed regions shown in Supplementary Fig. 10c

Supplement: Supplementary file 13 — Source data [file 41467_2026_75109_MOESM13_ESM.pdf]
