# Supplementary material for: Structural basis of the regulation by CDK11 kinase of early spliceosome activation and evidence for its proofreading by DHX15 helicase
Source: Nat Commun. 2026 Jul 3;17:5845. doi: 10.1038/s41467-026-75109-2 (PMC13332222; doi:10.1038/s41467-026-75109-2)
Supplement: Supplementary file 12 — Transparent Peer Review file [file 41467_2026_75109_MOESM12_ESM.pdf]

nature portfolio

Peer Review File

Structural basis of the regulation by CDK11 kinase of early spliceosome activation and evidence for its proofreading by DHX15 helicase

Corresponding Author: Professor Reinhard Lührmann

This file contains all reviewer reports in order by version, followed by all author rebuttals in order by version.

Version 0:

Reviewer comments:

Reviewer #1

(Remarks to the Author)
Zhang, Kumar et al combine structural cryo-EM and biochemical crosslinking data to provide a detailed description of the molecular arrangements of human spliceosomes assembled on a model pre-RNA substrate upon inhibition of kinase CDK11 by the small molecule OTS964. Combined with previous pioneering structural work by the Lührmann & Stark teams and other groups, the results provide important new insights into one key step in the activation of the spliceosome, and into the molecular mechanisms that proofread the proper conformational changes that ensue the stepwise assembly of splicing complexes on splice site regions. The paper not only provides a snapshot of an important transition step but also novel models of general value to understand conformational triggers and mechanisms of proofreading.

Specifically, the structure of the OTS964-inhibited spliceosomal complex provides unexpected insights on how phosphorylation of a splicing factor (SF3B1 N-terminal domain) can 1) serve as a signal to trigger complex conformational transitions (including a striking relocation of SF3B6 required for progression to complex Bact formation, concomitant with major rearrangements in RNA:RNA, RNA:protein and protein:protein contacts) revealing novel driving forces in the process, and 2) compete with the association of proofreading helicase DHX15 and several co-factors, including G-patch-containing proteins and the scaffolding factor SR140, providing a novel model for proofreading activities by helicase complexes.

Collectively, the new structure illustrates the surprising complexity of what previously considered to be a single step for the activation of the fully assembled spliceosome, revealing key roles for post-translational modifications and their interplay with proofreading complexes that had not been implicated at this stage in the process. On this basis I am happy to support publication of the manuscript in Nature Communications. The study will certainly fuel new thinking and work on this and other steps of the complex process of spliceosome assembly and activation, as well as more generally on proofreading of ribonucleoprotein processes.

A potential issue with the manuscript is the high density of the data and information provided, as well as the abundance of sophisticated structural arguments made, which may be overwhelming for non-specialists. While the Discussion is an example of clarity, highlighting the key points of the paper in a clear and enticing manner, the Results section is indeed very dense and at points difficult to follow. In my opinion the following revisions would help to improve the text:

1) Summary: understanding some of the paper highlights requires background information that is not introduced earlier on in the summary, which may make it difficult to follow, specially to gather their general significance, for non-specialists. Examples include the sentences “This explains the stalled position…were not first to relocate” and “We also located the binding of the RNA helicase DHX15…and G-patch proteins”. Non-specialist readers may wonder about the significance of “passing along the PRP8RT domain”, or the significance of G-patch proteins for the recruitment of an RNA helicase.
2) Introduction: along the same lines, it may be helpful for the general reader to explain concepts such as “catalytic U6 snRNA triplex” (line 63) and clarify the difference in activities between upstream helicases that “remodel off-pathway”” (line 98) and DHX15, which “subsequently dismantles” these complexes (line 99).
3) Line 127: it would be helpful for the general reader to know the rationale for the choice of the PM5-10 pre-mRNA (the word “construct” may not be necessary here).
4) Line 128: it may be useful to state in the main text the concentration used of OTS964 and its IC50.

5) Line 132: it will be helpful to indicate the identity of the tag used (MS2?) for affinity chromatography of the complexes.
6) Line 166: "5' domain of U2 snRNP" may require clarification for non-specialists the first time the term is used, e.g. proteins associated with the 5' x nucleotides of U2 snRNA.
7) Line 207: it may not be obvious why the extended U6 ISL "elicits" (rather than being a consequence of other conformational changes) a large-scale downward movement of the 5' domain of U2 snRNP, i.e. why it is causative of the movement.
8) While the extensive Extended Data Figures, Supplementary Tables and specially the supporting Movies are very helpful to get the message across, entire sections (e.g. those covering the "Coordination by SMU1/RED…" and "Location of NTC/NTR…") are illustrated through Supplementary Materials which the average reader may not be inclined to use extensively. I would recommend the authors to consider including a key summary Figure of each of these in the main set of Figures.
9) Line 294: RRM protein SF3B6: it may be helpful for the general reader to introduce the concept of the RRM fold.
10) Lines 312-313, lines 342-344, section starting in line 346 and Discussion: while the collective evidence supports a key role of CDK11-dependent phosphorylation of SF3B1N as the major driving force for triggering the repositioning of SF3B6, is there a clear model for how this occurs, i.e. what key molecular contacts between SF3B6 and HRs 12-14 would be broken (and/or contacts with RES protein SNIP1 facilitated) by the access of CDK11 complex / phosphorylation of SF3B1 T426/434/436?
11) Lines 442-450: the crosslinking data suggest a direct interaction between SR140 and SUGP1. However the groups of Manley, Rabadan and Liu have reported a direct interaction of SUGP1 with SF3B1 as an alternative way to recruit SUGP1 (and consequently DHX15), with the appealing added value of potentially explaining the mechanism by which cancer-associated mutations in SF3B1, by disrupting these interactions, cause cryptic splice site utilization: PMID: 31474574, PMID: 32332164, PMID: 40027711, PMID: 40714635, PMID: 36459648, PMID: 37977822. The authors could consider to extend the discussion of these findings.
12) Somewhat related to the previous point, recent work by Damianov and Black (both reference 48 as well as PMID: 40138349 -again potentially explaining the effects of cancer mutations in SF3B1-) indicates that association of SR140 (along with CHERP and RBM17/SPF45) occurs at early stages of branch point recognition on chromatin-associated nascent transcripts. Is this mode of DHX15 recruitment additional / overlapping / complementary to the model presented by the authors in the context of SF3B1N phosphorylation / SF3B6 translocation?
13) The authors could consider moving the final paragraph of Results (lines 480 – 491) to the Discussion section.
14) Lines 552 – 554: the absence of SF3B6 and of a conserved Nt region in the yeast SF3B1 counterpart are indeed striking and probably merit an extended discussion: how do yeast spliceosomes transition between B and Bact complexes, then? E.g. does CDK11-mediated phosphorylation of Hsh155 occur in S. cerevisiae? Does OTS964 inhibit spliceosome assembly in budding yeast?

Reviewer #2

(Remarks to the Author)
The manuscript by Zhang et al. uses OTS964 and a PM5-10 pre-mRNA construct to determine, by cryo-EM and mass spectrometry, the structure and composition of spliceosomes blocked by inhibition of CDK11, a kinase that phosphorylates the SF3B1 N-terminus and is essential for spliceosome activation. The authors identify a spliceosome intermediate termed PM5-10 pre-Bact-OTS, which proceeds a previously characterized intermediate, pre-Bact-1, identified by the same group (PMID: 33243851). The pre-Bact-OTS complex is characterized by dissociation of the U4 snRNP, the presence of NTC, NTR, and IBC proteins, and the formation of a previously unobserved step in the establishment of the U2/U6 snRNA catalytic core. The authors further identify roles for the SMU1/RED complex in early stages of spliceosome remodeling and suggest a crucial role for CDK11-mediated phosphorylation of the SF3B1 N-terminus in the transition of what they term the SF3B6/SF3B1(394–430) module from SF3B1 HEAT repeats 12–14 to repeats 1–2. To characterize the roles of phosphorylated SF3B1, they use crosslinking approaches and identify three threonine residues involved in SF3B6/SF3B1(394–430) module transition and SNIP1-mediated recruitment of the RES complex to the SF3B1 HEAT repeats 1–2. Finally, using their PM5-10 pre-Bact-OTS structures, crosslinking data, and AlphaFold3 modeling, the authors determine the location and propose functions of the DHX15 helicase and three G-patch-associated proteins (SUGP1, CHERP, and SPF45) in quality control during early stages of CDK11-dependent spliceosome activation.
Overall, the manuscript provides new mechanistic insights into spliceosome activation, is well written and timely, and will be of interest to the readership of Nature Communications. However, before publication, I have the following questions, comments, and suggestions that need to be addressed:
1. Page 3, lines 70–77: This recent paper describing several structures of activated spliceosomes (PMID: 39068178) should also be cited, and these structures should be compared in the Discussion with those observed following CDK11 inhibition.
2. Page 3, line 84: The authors should additionally cite a recent paper characterizing SF3B1 phosphorylation (PMID: 41428738) and at page 4, line 88, this paper (PMID: 31511426) should be also cited.
3. Figure 1a and Extended Data Figure 1a, c: The PM5-10 pre-mRNA construct was used, but this substrate contains a very short intron (and a short distance between the 5′ splice site and the branch site). Do the authors observe identical results when other splicing substrates with longer introns, such as MINX or AdML, are used? In addition, controls performed in the absence or at low concentrations of ATP and under low Mg^{2+} conditions should be included to allow a more rigorous comparison between B and Bact-OTS complexes.
4. Page 5, lines 132–147: The composition of OTS964-stalled complexes assembled on PM5-10 pre-mRNA differs from chromatin-associated spliceosome complexes in cells after OTS964 treatment (PMID: 36104565), specifically with respect to the absence of U4 snRNA and the presence of the IBC complex. There may be several reasons for these differences, and the authors should at least comment on them (see also points 3, 5, and 6).
5. SAD1 and SNU66 inhibit BRR2 function but are found as part of the Bact-OTS spliceosome (see Supplementary Table 1,

where SAD1 and SNU66 are abundant in Bact-OTS). Do the authors expect BRR2 to be active and capable of unwinding U4 snRNA when SAD1 and SNU66 are present? In addition, neither of these proteins is shown in any of the structures depicting the Bact-OTS complex.
6. TXNL4A (DIM1) and FBP21 (WBP4) are identified in the crosslinking dataset. The a-finger of PRPF8 interacts with PRP31 and DIM1 to stabilize U4/U6 stem I. Therefore, the presence of DIM1 is also inconsistent with U4 snRNA having been unwound in the Bact-OTS complex.
7. SMU1/RED were previously shown to be required for efficient splicing of short introns (PMID: 31409787). Are the newly identified roles of this complex described on page 9 restricted to spliceosomes assembled on short introns? See also point 3.
8. Page 19, line 520: "HR 1–2" should be used instead of "HR 1–3".
9. In the Discussion (page 19, line 529), the statement should omit the term "phosphorylated," as SF3B1 is not phosphorylated in the pre-Bact-OTS complex, but rather in the pre-Bact and Bact complexes. Similarly, in the Extended Data Figure 6e T426, T434 and T436 are labeled by the same color irrespectively of their phosphorylation status at different steps of the spliceosome activation (Bact-OTS, Bact-1/2 and Bact) which is misleading. This needs to be corrected and clarified. It is also not clear why amino acids 67 (SF3B6) and 394 (SF3B1) are highlighted in the Extended Data Figure 6e, please explain.
10. Page 5, line 118 and elsewhere: The statement "Aberrant spliceosomes would impair CDK11 recruitment (and thus SF3B1 phosphorylation)…" contradicts the data presented in Data S1, where CDK11-CCNL-SAP30BP are clearly crosslinked to the spliceosome in the pre-Bact-OTS complex. This indicates that aberrant spliceosomes do not impair CDK11 recruitment; rather, the recruited kinase is inhibited by OTS964 in the pre-Bact-OTS complex. In a similar vein, while SAP30BP may play a role in recruiting the CDK11 complex to certain spliceosome intermediates (as discussed on page 22, line 616), the mechanism proposed in reference 52 is difficult to reconcile with the current findings, since SAP30BP recruitment in that study depends on SF3B1 phosphorylation, which itself depends on CDK11 activity. In this context, it would also be important to discuss the role of SPF45, which has been shown in several studies to be recruited to unphosphorylated SF3B1 in earlier spliceosome intermediates and to function predominantly in the splicing of short introns or in alternative splicing regulation (PMID: 17589525, PMID: 34389706, PMID: 38065098). Once again, this raises the question of the extent to which the spliceosome structures described in this manuscript are specific to short introns due to the use of PM5-10 pre-mRNA, and to what extent the conclusions can be generalized, given that OTS964 blocks constitutive splicing in cells (PMID: 36104565, PMID: 38059508). These potential limitations should be discussed.

Reviewer #3

(Remarks to the Author)
Zhang et al. present a structural and functional analysis of a novel early-activated spliceosome intermediate, termed pre-Bact-OTS complex, stalled by the CDK11 inhibitor OTS964. By integrating single-particle cryo-EM, crosslinking mass spectrometry, biochemical assays, and AlphaFold3 modeling, the authors elucidate the mechanism through which CDK11-mediated phosphorylation of SF3B1 regulates spliceosome activation and reveal how the helicase DHX15 may proofread this process. While post-translational modifications (PTMs) such as SF3B1 phosphorylation have been recognized as important regulatory events in splicing, the precise structural and functional consequences of this phosphorylation have remained unclear. This study demonstrates that phosphorylation of this core splicing factor triggers a critical structural rearrangement, thereby establishing a checkpoint for the proofreading of spliceosome assembly. Importantly, the work identifies the recruitment of DHX15 and its associated G-patch proteins to this intermediate, with SR140 serving as a key binding hub. Although DHX15 is known for its role in dismantling the ILS complex at the end of the splicing cycle and has recently been implicated in proofreading early assembly stages, this study significantly expands its functional repertoire, positioning DHX15 as a general proofreading factor active at multiple points in the splicing cycle.

Overall, this work provides crucial insights into how PTMs regulate the splicing cycle and how the spliceosome in higher eukaryotes may take advantage of such modifications as checkpoints to ensure fidelity. The implications of this research could extend beyond the splicing field, offering valuable perspectives also for other RNP biogenesis processes. Thus, I am happy to support publication of the manuscript in Nature Communications.

A few comments:

1. The Results section is generally dense and may be challenging for readers not deeply familiar with spliceosome structural biology. Given the broad readership of this journal, providing additional context and simplifying some of the detailed descriptions would greatly improve accessibility and broaden the impact of the work.

2. The discussion on the absence of this kinase-switch system in S. cerevisiae is interesting and highlights evolutionary complexity in humans/higher eukaryotes. It raises the question of whether this mechanism is conserved in other organisms such as S. pombe or C. elegans, which possess an SF3B6 counterpart. A brief bioinformatic analysis commenting on the conservation of SF3B6, the N-terminal region of SF3B1, and CDK11 across species would be a valuable addition.

3. The mechanism of CDK11 complex recruitment to the spliceosome remains unclear. Specifically, why is CDK11 recruited specifically to this intermediate, and not to others where the SF3B1 N-terminus might also be accessible? Expanding the discussion on this point would be beneficial.

4. The Results section includes a segment on the NTC/NTR complex and notes that IBC proteins are loosely associated. The implications of these observations are not discussed later. A brief comment on the potential significance of these

associations in the Discussion would be helpful.

Version 1:

Reviewer comments:

Reviewer #1

(Remarks to the Author)
The authors have adequately addressed the issues raised in my previous report and I am happy to recommend publication of the paper, which I expect to have an important impact in the field of spliceosome function and, more generally, the function of complex ribonucleoprotein machines.

Reviewer #2

(Remarks to the Author)
The authors responded to my questions and made the appropriate adjustments to the text. I recommend publication in Nature Communications

Reviewer #3

(Remarks to the Author)
We appreciate the authors' detailed responses to our comments. Based on the revisions and rebuttal, we now agree that the manuscript is suitable for publication.

Open Access This Peer Review File is licensed under a Creative Commons Attribution 4.0 International License, which permits use, sharing, adaptation, distribution and reproduction in any medium or format, as long as you give appropriate credit to the original author(s) and the source, provide a link to the Creative Commons license, and indicate if changes were made.
In cases where reviewers are anonymous, credit should be given to 'Anonymous Referee' and the source.
The images or other third party material in this Peer Review File are included in the article's Creative Commons license, unless indicated otherwise in a credit line to the material. If material is not included in the article's Creative Commons license and your intended use is not permitted by statutory regulation or exceeds the permitted use, you will need to obtain permission directly from the copyright holder.
To view a copy of this license, visit https://creativecommons.org/licenses/by/4.0/

Point-by-Point Response to Reviewer Comments

Reviewer 1

Zhang, Kumar et al combine structural cryo-EM and biochemical crosslinking data to provide a detailed description of the molecular arrangements of human spliceosomes assembled on a model pre-RNA substrate upon inhibition of kinase CDK11 by the small molecule OTS964. Combined with previous pioneering structural work by the Lührmann & Stark teams and other groups, the results provide important new insights into one key step in the activation of the spliceosome, and into the molecular mechanisms that proofread the proper conformational changes that ensue the stepwise assembly of splicing complexes on splice site regions. The paper not only provides a snapshot of an important transition step but also novel models of general value to understand conformational triggers and mechanisms of proofreading.
Specifically, the structure of the OTS964-inhibited spliceosomal complex provides unexpected insights on how phosphorylation of a splicing factor (SF3B1 N-terminal domain) can 1) serve as a signal to trigger complex conformational transitions (including a striking relocation of SF3B6 required for progression to complex Bact formation, concomitant with major rearrangements in RNA:RNA, RNA:protein and protein:protein contacts) revealing novel driving forces in the process, and 2) compete with the association of proofreading helicase DHX15 and several co-factors, including G-patch-containing proteins and the scaffolding factor SR140, providing a novel model for proofreading activities by helicase complexes.
Collectively, the new structure illustrates the surprising complexity of what previously considered to be a single step for the activation of the fully assembled spliceosome, revealing key roles for post-translational modifications and their interplay with proofreading complexes that had not been implicated at this stage in the process. On this basis I am happy to support publication of the manuscript in Nature Communications. The study will certainly fuel new thinking and work on this and other steps of the complex process of spliceosome assembly and activation, as well as more generally on proofreading of ribonucleoprotein processes.
A potential issue with the manuscript is the high density of the data and information provided, as well as the abundance of sophisticated structural arguments made, which may be overwhelming for non-specialists. While the Discussion is an example of clarity, highlighting the key points of the paper in a clear and enticing manner, the Results section is indeed very dense and at points difficult to follow. In my opinion the following revisions would help to improve the text:

1) Summary: understanding some of the paper highlights requires background information that is not introduced earlier on in the summary, which may make it difficult to follow, specially to gather their general significance, for non-specialists. Examples include the sentences "This explains the stalled position…were not first to relocate" and "We also located the binding of the RNA helicase DHX15…and G-patch proteins". Non-specialist readers may wonder about the significance of "passing along the PRP8RT domain", or the significance of G-patch proteins for the recruitment of an RNA helicase.

We have provided more background information in the rewritten Summary

2) Introduction: along the same lines, it may be helpful for the general reader to explain concepts such as "catalytic U6 snRNA triplex" (line 63) and clarify the difference in activities between upstream helicases that "remodel off-pathway"" (line 98) and DHX15, which "subsequently dismantles" these complexes (line 99).

As suggested, we have rewritten those sentences as follows

"*catalytic U6 snRNA triplex*"***:*** Tertiary interactions between a bulged-out nucleotide of the U6 ISL (U74 in humans) and the U6 catalytic triad AGC nucleotides, which are part of U2/U6 helix Ib, as well as the U6 GA dinucleotide directly downstream of its ACAGA box, lead to formation of the catalytic U6 snRNA triplex and enables U6 to position two Mg^{2+} ions that participate directly in splicing catalysis [2-4]. (lines 62-66 rev. ms)

"*remodel off-pathway*": Several RNA helicases that drive the rearrangements required during spliceosome assembly have also been proposed as establishing fidelity in splicing by a kinetic proofreading mechanism [24-27] in which the DEAD/H-box ATPase promotes an optimum substrate by acting after a productive step and by rejecting a suboptimal substrate (or incorrectly structured complex) and consequently driving the substrate into an "off-pathway" state[8,27,28]. Such aberrant, off-pathway spliceosomes may then be dismantled by the RNA helicase DHX15 (also termed PRP43). DHX15 is well known for its role in dismantling the terminal ILS spliceosome, in cooperation with its ATPase-activating G-patch protein TFIP11[29-31] (lines 96-104)

3) Line 127: it would be helpful for the general reader to know the rationale for the choice of the PM5-10 pre-mRNA (the word "construct" may not be necessary here).

We have expanded the sentence as follows:

To elucidate the molecular architecture of an OTS964-stalled spliceosome, we used PM5-10 pre-mRNA, which lacks the second exon (Fig. 1a). It allows efficient B-to-B^{act} complex transformation in HeLa splicing extract [18] (Extended Data Fig. 1a), but it stalls spliceosome formation at the activated (B^{act}) stage. In the presence of 0.2 µm OTS964; however, PM5-10 B^{act} complex formation is completely inhibited, and a significant fraction of stalled spliceosomes migrates in native gels in a manner similar to that of B/pre-B^{act} stage complexes (Extended Data Fig. 1a). The IC50 of OTS964 for CDK11 is less than 100 nm [7,23]. (lines 127-134)

4) Line 128: it may be useful to state in the main text the concentration used of OTS964 and its IC50.

Done; see point 3)

5) Line 132: it will be helpful to indicate the identity of the tag used (MS2?) for affinity chromatography of the complexes.

Done

6) Line 166: "5' domain of U2 snRNP" may require clarification for non-specialists the first time the term is used, e.g. proteins associated with the 5' x nucleotides of U2 snRNA.

In the revised version of the manuscript, we have clarified the term "5' domain of U2 snRNP" in the new Summary. (lines 33-40)

7) Line 207: it may not be obvious why the extended U6 ISL "elicits" (rather than being a consequence of other conformational changes) a large-scale downward movement of the 5' domain of U2 snRNP, i.e. why it is causative of the movement.

In the revised version we provide additional reasons why we believe that the formation of the U6 ISL is the likely driving force for moving the U2 5' domain closer to the spliceosome core:

The ISL structure in our pre-$B^{act\text{-}OTS}$ complex is similar to yeast U6 snRNA within the U6 snRNP complex, which also exhibits an extended ISL structure that includes the invariant AGC triad at the top of the ISL stem [37,38]. Thus, the extended ISL appears to be a default structure into which this part of U6 RNA folds immediately after BRR2-helicase-mediated unwinding of the U4/U6 stems I and II within the B complex. (lines 188-193; see also lines 210-212)

8) While the extensive Extended Data Figures, Supplementary Tables and specially the supporting Movies are very helpful to get the message across, entire sections (e.g. those covering the "Coordination by SMU1/RED…" and "Location of NTC/NTR…") are illustrated through Supplementary Materials which the average reader may not be inclined to use extensively. I would recommend the authors to consider including a key summary Figure of each of these in the main set of Figures.

We agree with the referee. We have now added a new Figure 4 in the revised version, entitled "Structural organization of SMU1/RED and NTC proteins in pre-$B^{act\text{-}OTS}$ complexes"; it shows a new schematic, depicting the coordinated movements of the U2 snRNP 5' domain, the SMU1/RED tetrameric complex and the BRR2 helicase during the B-to-pre-$B^{act\text{-}OTS}$ transition (panels a and b). In the new Figure 4c we show the intermediate docking sites of the WD40 domain of the key NTC protein PLRG1 and of the U6 LSm complex at the PRP8-HB domain in the pre-$B^{act\text{-}OTS}$ complex. In the Extended Data Figures 4 and 5 of the revised manuscript version we have omitted panels e and a, respectively, from the old Figures. The old Figures 4–6 now become Figures 5–7, respectively.

9) Line 294: RRM protein SF3B6: it may be helpful for the general reader to introduce the concept of the RRM fold.

In the revised version we explain the term RRM: …we noticed that the position of the SF3B6 (p14) protein, which contains an RNA recognition motif (RRM), at SF3B1^{HEAT} is strikingly different…. (lines 296-298)

10) Lines 312-313, lines 342-344, section starting in line 346 and Discussion: while the collective evidence supports a key role of CDK11-dependent phosphorylation of SF3B1N as the major driving force for triggering the repositioning of SF3B6, is there a clear model for how this occurs, i.e. what key molecular contacts between SF3B6 and HRs 12-14 would be broken (and/or contacts with RES protein SNIP1 facilitated) by the access of CDK11 complex / phosphorylation of SF3B1 T426/434/436?

Unfortunately, the resolution of the U2 5' domains in the cryo-EM structures of our pre-B$^{act\text{-}OTS}$ and later pre-B^{act} complexes are not good enough to allow us to answer this interesting question at the atomic level. However, we consider that the positioning of the three threonines T426/434/436 at or close to the interfaces between the SF3B6 module and SF3B1^{HEAT} in pre-B$^{act\text{-}OTS}$ (in their unphosphorylated states) and in pre-B$^{act\text{-}1}$ and later activated stages (in their phosphorylated states) is intriguing and raises the interesting possibility that phosphorylation of those threonines may play a dual role in the relocation of the SF3B6 model, as discussed in our manuscript (see lines 370-377). Clearly, higher resolution structures will be needed to clarify this point in the future.

11) Lines 442-450: the crosslinking data suggest a direct interaction between SR140 and SUGP1. However the groups of Manley, Rabadan and Liu have reported a direct interaction of SUGP1 with SF3B1 as an alternative way to recruit SUGP1 (and consequently DHX15), with the appealing added value of potentially explaining the mechanism by which cancer-associated mutations in SF3B1, by disrupting these interactions, cause cryptic splice site utilization: PMID: 31474574, PMID: 32332164, PMID: 40027711, PMID: 40714635, PMID: 36459648, PMID: 37977822. The authors could consider to extend the discussion of these findings.

We agree with reviewer 1 that the direct interaction of SUGP1 with HEAT repeats 2–7 of SF3B1 plays a major role in the stable complex formation between SUGP1, SF3B1^{HEAT} and DHX15, as indicated by the combinatorial structural biology approach of the groups of Manley, Galej and Liu. However, our observation that SUGP1 interacts with SR140 does not contradict the studies by Zhang *et al*. (PMID: 37977822; ref. 36). First of all, SUGP1 binds to SR140 through its N-terminally located SURP domains, far upstream of the SUGP1 regions that directly interact with SF3B1^{HEAT}; that is, the binding of SUGP1 to SR140 and its binding to SF3B1^{HEAT} are not mutually exclusive. In our view, SR140 functions as a hub for several G-patch proteins, including SUGP1, facilitating kinetically the interaction of SUGP1 with SF3B1^{HEAT}, and consequently also the recruitment of DHX15 to the spliceosome. In addition, we would like to point out that Zhang *et al*. (PMID: 37977822, ref. 36) propose in their study that SUGP1 plays a major role in the recruitment of U2 snRNP to the branch site of the pre-mRNA intron, and would thus interact with the spliceosome already very early, directly after E-complex formation, while in our study, SUGP1 (in complex with SR140) interacts with the spliceosome much later, during spliceosome activation. It is therefore possible that the requirements for complex formation between SUGP1, the U2 5′ domain and DHX15 may differ somewhat at different stages of spliceosome assembly. In the revised version of our manuscript, we have referred to this point as follows:

Recent studies have revealed that SUGP1 co-operates with DHX15 in proofreading and dismantling defective spliceosomes, although without evidence for the participation of the SR140-CHERP-SPF45 complex[32-35]. While in some cases it has been suggested that such proofreading may take place during very early stages of spliceosome assembly, such as in the pre-A or A complex [35], in our study, SUGP1 (in complex with SR140) interacts with the spliceosome much later, i.e., during spliceosome activation. It is therefore possible that the requirements for complex formation between SUGP1, the U2 5' domain and DHX15 may differ at different stages of spliceosome assembly (see also below). (lines 652-660)

12) Somewhat related to the previous point, recent work by Damianov and Black (both reference 48 as well as PMID: 40138349 -again potentially explaining the effects of cancer mutations in SF3B1-) indicates that association of SR140 (along with CHERP and RBM17/SPF45) occurs at early stages of branch point recognition on chromatin-associated nascent transcripts. Is this mode of DHX15 recruitment additional / overlapping / complementary to the model presented by the authors in the context of SF3B1N phosphorylation / SF3B6 translocation?

In the work of Damianov, Black and coworkers (reference 61), U2 snRNP was purified from chromatin following digestion of the isolated chromatin with DNase and RNase. As the authors discuss in their paper, it is therefore difficult to decide whether their isolated U2 snRNP-branch site RNA-DHX15-SR140/CHERP/SPF45 complex is derived from an early, A-stage spliceosome or from a later assembly stage such as pre-B or B complexes. However, as Damianov *et al*. have purified their U2 snRNP complex with a tagged RBM5 (or RBM10) G-patch protein, and both proteins are essentially absent from our pre-B$^{act\text{-}OTS}$ complex preparation, this would indicate that their U2 snRNP complex is not derived from an early, activated spliceosome. Therefore, it will be interesting to see whether the SR140/CHERP/SPF45 complex may also play a role in tethering the G-patch proteins RBM10 and/or RBM15 to SF3B1^{HEAT}. If so, this would indeed raise the possibility that SR140/CHERP/SPF45 may act repeatedly during different spliceosome remodeling steps, *en route* to spliceosome activation. We have included the following sentences in the revised version of the manuscript:

Notably, other G-patch proteins, such as RBM5 and RBM10, have also been implicated in supporting DHX15-mediated quality control of early spliceosome assembly[61,62]. For example, Damianov et al.[61] purified U2 snRNP/branch site complexes from RNAase-treated chromatin, together with DHX15 and the SR140-SPF45-CHERP protein complex, raising the possibility that the latter complex may also play a role in tethering the G-patch proteins RBM10 and/or RBM5 to SF3B1^{HEAT}. As RBM5/10 proteins are absent in our pre-B$^{act\text{-}OTS}$ complex, it appears likely that their U2 snRNP complex is derived from an earlier spliceosome assembly stage, suggesting that SR140-SPF45-CHERP may act repeatedly during different spliceosome remodeling steps en route to spliceosome activation. (lines 666-677)

13) The authors could consider moving the final paragraph of Results (lines 480 – 491) to the Discussion section.

As we have expanded significantly the Discussion in the revised version, we would prefer to keep this paragraph at the end of the Results section, though in a slightly shortened form. (lines 483-486)

14) Lines 552 – 554: the absence of SF3B6 and of a conserved Nt region in the yeast SF3B1 counterpart are indeed striking and probably merit an extended discussion: how do yeast spliceosomes transition between B and Bact complexes, then? E.g. does CDK11-mediated phosphorylation of Hsh155 occur in S. cerevisiae? Does OTS964 inhibit spliceosome assembly in budding yeast?

We would like to thank reviewer 1 and reviewer 3 (point 2) for addressing this interesting point. As suggested by reviewer 3, we have carried out a brief bioinformatic analysis of the evolutionary conservation of SF3B6, CDK11 and numerous TP/SP dipeptide repeats in the N-terminal region of SF3B1 (SF3B1$^{N\text{-}TP/SP}$). As indicated in the new Extended Data Figures 9 and 10, SF3B1$^{N\text{-}TP/SP}$, SF3B6 and CDK11 kinase are highly conserved across metazoans and plants. In contrast, in *S. cerevisiae* and numerous other fungi, the SF3B6-SF3B1$^{N\text{-}TP/SP}$-CDK11 axis is absent. Only few organisms such as *S. pombe* (new Extended Data Fig. 9) contain SF3B6, but lack a TP/SP rich N-terminus in the *S. pombe* SF3B1 and a genuine homolog of CDK11(PMID: 39245599; ref. 57), suggesting that the relocation of SF3B6 at SF3B1^{HEAT} during early activation of the *S. pombe* spliceosome requires a distinct regulatory mechanism. Alternatively, the SF3B6 module may be less stably attached to the C-terminal region of SF3B1^{HEAT} in *S. pombe*, so that it may already be destabilized and relocated at SF3B1^{HEAT} during the large-scale translocation of the U2 5′ domain during early activation of the spliceosome. Interestingly, the intron-rich yeast *Cryptococcus neoformans* does contain an SF3B6 homolog, an SF3B1^{N} domain with a short TP-rich region, and putative homologs of CDK11 kinase, cyclin and SAP30BP[56] (Extended Data Fig. 9 and 10). In summary, there appears to be a strong correlation between the conservation of a TP/SP dipeptide-rich N-terminal domain of SF3B1, the CDK11 kinase and SF3B6 across species during evolution; this further suggests that the CDK11-mediated SF3B6 "protein switch" we describe in the human spliceosome is a conserved regulatory mechanism in organisms with complex splicing architectures.

In the revised manuscript, we describe the evolutionary conservation of the SF3B6-SF3B1$^{N\text{-}TP/SP}$-CDK11 axis in the Discussion (lines 560-579). The results of our bioinformatic analysis are shown in the new Extended Data Figures 9 and 10. The old Extended Data Figure 9 becomes the new Extended Data Figure 11. We also include this point in the Summary of the new version of the manuscript.

Interestingly, our bioinformatic analysis also revealed that the **SMU1/RED proteins**, which guide the U2 snRNP 5′ domain to the intermediate checkpoint state in pre-B$^{act\text{-}OTS}$, are absent in all organisms that lack the CDK11-mediated SF3B6 protein switch, including *S. cerevisiae* (included in the new Extended Data Figure 9). Consistently, the yeast *C. neoformans*, however, also expresses conserved SMU1 and RED homologs (Extended

Data Fig. 9). These observations underpin the idea that the role of SMU1/RED in coordinating the sophisticated remodeling events of U2 and U6 snRNPs as well as BRR2, is evolutionarily connected to the conserved SF3B6-SF3B1$^{N\text{-}TP/SP}$-CDK11 axis. The absence of this regulatory module in *S. cerevisiae* suggests that the early activation phase of the yeast spliceosome might follow a less complex remodeling pathway than that of humans. We have described this point in the Discussion of the revised manuscript (lines 580-594).

Reviewer 2

The manuscript by Zhang et al. uses OTS964 and a PM5-10 pre-mRNA construct to determine, by cryo-EM and mass spectrometry, the structure and composition of spliceosomes blocked by inhibition of CDK11, a kinase that phosphorylates the SF3B1 N-terminus and is essential for spliceosome activation. The authors identify a spliceosome intermediate termed PM5-10 pre-Bact-OTS, which proceeds a previously characterized intermediate, pre-Bact-1, identified by the same group (PMID: 33243851). The pre-Bact-OTS complex is characterized by dissociation of the U4 snRNP, the presence of NTC, NTR, and IBC proteins, and the formation of a previously unobserved step in the establishment of the U2/U6 snRNA catalytic core. The authors further identify roles for the SMU1/RED complex in early stages of spliceosome remodeling and suggest a crucial role for CDK11-mediated phosphorylation of the SF3B1 N-terminus in the transition of what they term the SF3B6/SF3B1(394–430) module from SF3B1 HEAT repeats 12–14 to repeats 1–2. To characterize the roles of phosphorylated SF3B1, they use crosslinking approaches and identify three threonine residues involved in SF3B6/SF3B1(394–430) module transition and SNIP1-mediated recruitment of the RES complex to the SF3B1 HEAT repeats 1–2. Finally, using their PM5-10 pre-Bact-OTS structures, crosslinking data, and AlphaFold3 modeling, the authors determine the location and propose functions of the DHX15 helicase and three G-patch-associated proteins (SUGP1, CHERP, and SPF45) in quality control during early stages of CDK11-dependent spliceosome activation.
Overall, the manuscript provides new mechanistic insights into spliceosome activation, is well written and timely, and will be of interest to the readership of Nature Communications. However, before publication, I have the following questions, comments, and suggestions that need to be addressed:

1.Page 3, lines 70–77: This recent paper describing several structures of activated spliceosomes (PMID: 39068178) should also be cited, and these structures should be compared in the Discussion with those observed following CDK11 inhibition.

We have included the requested references in the revised version of the manuscript. We have also referred to the cryo-EM structures described in PMID: 39068178 in the following way:

Recently, two studies, using small-molecule splicing inhibitors, revealed cryo-EM structures of three human activated-spliceosome precursors, termed pre-B^{act-1} and pre-B^{act-2} [5], or just pre-B^{act} [16], the latter representing an intermediate assembly stage that arises after pre-B^{act-2} but before the formation of mature B^{act} complexes**.** (lines 69-73)

As the only pre-B^{act} structure captured in this paper is positioned further downstream of pre-B^{act-2}, while our pre-B$^{act-OTS}$ structure is positioned upstream of pre-B^{act-1}, we do not think that it is necessary to compare the two structures directly.

2. Page 3, line 84: The authors should additionally cite a recent paper characterizing SF3B1 phosphorylation (PMID: 41428738) and at page 4, line 88, this paper (PMID: 31511426) should be also cited.

Done

3a. Figure 1a and Extended Data Figure 1a, c: The PM5-10 pre-mRNA construct was used, but this substrate contains a very short intron (and a short distance between the 5' splice site and the branch site). Do the authors observe identical results when other splicing substrates with longer introns, such as MINX or AdML, are used?

The PM5-10 pre-mRNA contains a 151-nt-long intron (distance between the 5′SS and BS) and is thus significantly longer than the 96-nt-long intron of MINX pre-mRNA. Moreover, the length of the polypyrimidine tract downstream of the intron's BS-A is also comparable between PM5-10 and MINX pre-mRNAs (16 nt vs. 21nt; not counting the 3′SS of the MINX intron, as this is missing in PM5-10). Finally, both poly-Py tracts share a run of 7 or 8 consecutive uridine nts, respectively, and should thus exhibit a similar affinity towards U2AF^{65}. Thus, we did not push for the structural analysis of spliceosomes assembled in the presence of OTS964 on other pre-mRNAs with similar sizes.

3b. In addition, controls performed in the absence or at low concentrations of ATP and under low Mg^{2+} conditions should be included to allow a more rigorous comparison between B and Bact-OTS complexes.

The rationale for these experiments is not obvious to us. We have recently published the currently most comprehensive and concise 3D cryo-EM structure of a human B complex that was stalled in the presence of ATPγS (PMID: 38383864, ref. 41). We showed previously (PMID: 27411562) that low Mg^{2+} and/or ATP concentrations also stall spliceosome formation at the B stage level, however, these complexes are biochemically more heterogenous than those stalled in the presence of ATPγS. Thus, the gold standard for a rigorous comparison of our pre-$B^{act\text{-}OTS}$ structure with a B complex cryo-EM structure is that of the $B^{ATP\gamma S}$ complex. To rule out possible structural ambiguities, we have repeated the reconstruction of the PM5-10 pre-$B^{act\text{-}OTS}$ cryo-EM structure three times, using three independent spliceosome purifications, which revealed essentially the same structures.

4. Page 5, lines 132–147: The composition of OTS964-stalled complexes assembled on PM5-10 pre-mRNA differs from chromatin-associated spliceosome complexes in cells after OTS964 treatment (PMID: 36104565), specifically with respect to the absence of U4 snRNA and the presence of the IBC complex. There may be several reasons for these differences, and the authors should at least comment on them (see also points 3, 5, and 6).

These apparent inconsistencies may be at least partially explained by the significant differences in the experimental design of the two studies. That is, while we discuss the near-atomic structure of an OTS964 stalled, defined spliceosome population, assembled *in vitro*, Hluchy *et al*. (PMID: 36104565, ref. 7) investigate the global composition of thousands of distinct pre-mRNA spliceosomes that remain associated with chromatin upon treatment of HTC116 cells with OTS964 *in vivo*. On the basis of retention of U4 snRNA in chromatin and reduced amounts of IBC proteins, they concluded that the chromatin-associated spliceosomes are stalled at a B-like stage, before BRR2-mediated activation. We note that chromatin-associated spliceosomes are assembled co-transcriptionally and are therefore very likely still to be tethered to the Pol II transcription machinery. Thus, it is conceivable that one or more proteins in chromatin-associated spliceosomes might require CDK11-mediated phosphorylation before BRR2-mediated dissociation of the U4/U6 RNA helices occurs, and subsequent phosphorylation of SF3B1. Alternatively, or additionally, the release of destabilized factors such as U4 snRNP may also follow different kinetics in chromatin-associated – and thus Pol II-tethered – spliceosomes, compared with spliceosomes assembled *in vitro*.

The apparent inconsistency regarding the different abundance of AQR in the spliceosomes of the two studies can also be explained. In the OTS964 stalled endogenous spliceosomes AQR is not detected by iCLIP. However, in the immunoblot analysis AQR is still present in reduced amounts (Fig. 4b; Hluchy et al. (PMID: 36104565). In our purified PM5-10 pre-$B^{act\text{-}OTS}$ preparation, AQR is clearly present, as determined by MS analysis; however, it is only flexibly tethered by protein–protein interactions to the peripheral part of the spliceosome complex and not positioned in the intron-bound position that AQR adopts at later activation stages. Thus, AQR would not be detectable in our preparation via iCLIP.

Interestingly, in a more recent proteomic study, Blazek and coworkers (PMID: 41904131, ref. 53) show that certain NTR proteins, such as SKIP and RBM22, are also present in the chromatin-associated spliceosomes isolated from HTC116 cells that had been incubated with OTS964. As the binding of SKIP to spliceosomes is mutually exclusive with that of the U4 snRNA-associated PRP31 protein, this would indicate that a fraction of the chromatin-associated spliceosomes had undergone at least partial activation. In view of the evolutionary conservation of the SF3B6-SF3B1$^{N\text{-}TP/SP}$-CDK11 axis, which we reveal in our study, we are confident that the CDK11-mediated molecular SF3B6 protein switch is one of the major structural rearrangements that is driven by CDK11 kinase during activation of human spliceosomes. We summarize the above points in the Discussion of our revised manuscript as follows:

In a recently published study, Hluchy et al.[7] blocked CDK11 kinase with OTS964 in vivo in HTC116 cells and investigated the effect of this on the global composition of thousands of distinct pre-mRNA spliceosomes that remain associated with chromatin. On the basis of retention of U4 snRNA in chromatin and reduced amounts of IBC proteins, they concluded that the chromatin-associated spliceosomes are stalled at a B-like stage, before BRR2-mediated activation. We note that chromatin-associated spliceosomes are assembled co-transcriptionally and are therefore very likely still to be tethered to the Pol II transcription machinery [59,60]. Thus, it is conceivable that one or more proteins in chromatin-associated spliceosomes might require CDK11-mediated phosphorylation before BRR2-mediated dissociation of the U4/U6 RNA helices occurs, and subsequent phosphorylation of SF3B1. Alternatively, or additionally, the release of destabilized factors such as U4 snRNP

may also follow different kinetics in chromatin-associated – and thus Pol II-tethered – spliceosomes, compared with spliceosomes assembled in vitro [59,60]. Interestingly, in a more recent proteomic study, Blazek and coworkers[53] show that certain NTR proteins, such as SKIP and RBM22, are also present in the chromatin-associated spliceosomes isolated from HTC116 cells that had been incubated with OTS964. As the binding of SKIP to spliceosomes is mutually exclusive with that of the U4 snRNA-associated PRP31 protein, this would indicate that a fraction of the chromatin-associated spliceosomes had undergone at least partial activation. (lines 607-625)

5. SAD1 and SNU66 inhibit BRR2 function but are found as part of the Bact-OTS spliceosome (see Supplementary Table 1, where SAD1 and SNU66 are abundant in Bact-OTS). Do the authors expect BRR2 to be active and capable of unwinding U4 snRNA when SAD1 and SNU66 are present? In addition, neither of these proteins is shown in any of the structures depicting the Bact-OTS complex.

Indeed, in the first version we only briefly described the biochemical composition of our purified spliceosomes that were stalled in the presence of OTS964, as we focused mainly on describing the cryo-EM structure of pre-$B^{act\text{-}OTS}$ assembly intermediate. To address the referee's point: First of all, it is important to note that our pre-$B^{act\text{-}OTS}$ spliceosome preparation still contains reduced amounts of U4 snRNA (as stated in line 139 of our original manuscript and shown in Ext. Fig. 1c.). Thus our pre-$B^{act\text{-}OTS}$ preparation still contains spliceosome populations that likely contain U4/U6.U5 tri-snRNPs. This explains why our MS analysis also reveals the presence of U4/U6.U5 tri-snRNP proteins such as SAD1, SNU66 and DIM1. This is also consistent with the observed crosslinks of WBP4. Whether the tri-snRNP is part of a B-stage or pre-B stage complex, or both, is difficult to decide in the absence of cryo-EM structures of this tri-snRNP-containing spliceosome population(s). In our original manuscript we note that the presence of OTS964 in splicing extracts also leads to an accumulation of A complexes (Ext. Fig 1a and as stated in lines 1213-1216 of our orig. manuscript). This may indicate that the stable incorporation of U4/U6.U5 tri-snRNP into a B complex during the pre-B to B complex transformation may be hindered/slowed down, because one or more early splicing factors such as SR proteins may require phosphorylation by CDK11[78,79]. In the revised version of our manuscript, we have added the following sentence to the legend of Extended Data Fig. 1c:

The reduced amounts of U4 snRNA indicates that some U4/U6.U5 tri-snRNP-containing populations of spliceosomes are also present in our pre-$B^{act\text{-}OTS}$ preparation, consistent with the presence of U4/U6.U5 tri-snRNP proteins such as SAD1, SNU66 and DIM1 in our MS analysis (Supplementary Table 1). (lines 1377-1381)

6. TXNL4A (DIM1) and FBP21 (WBP4) are identified in the crosslinking dataset. The a-finger of PRPF8 interacts with PRP31 and DIM1 to stabilize U4/U6 stem I. Therefore, the presence of DIM1 is also inconsistent with U4 snRNA having been unwound in the Bact-OTS complex.

The explanations given under point 5 also apply here.

7. SMU1/RED were previously shown to be required for efficient splicing of short introns (PMID: 31409787). Are the newly identified roles of this complex described on page 9 restricted to spliceosomes assembled on short introns? See also point 3.

In our previous paper (PMID: 31409787), we showed that SMU1/RED play a particularly important role for the activation of spliceosomes that are assembled on introns with a short distance (about 50 nt) between the 5′SS and BS, while depletion of SMU1/RED did not abolish the activation of PM5 or MINX pre-mRNAs, but significantly affected the efficiency and kinetics of splicing. While at that time we could only speculate about the possible role(s) of SMU1/RED in the transformation of B to B^{act} complexes, our new pre-$B^{act\text{-}OTS}$ structure now reveals that SMU1/RED coordinate the contra-rotating movements of U2 snRNP and BRR2 during early activation. As these RNP modules belong to the core of the spliceosome, their choreography should also be similar in the presence of large introns, and hence SMU1/RED likely facilitate the remodeling of any human pre-mRNA spliceosome. This idea is further supported by our observation that the role of SMU1/RED in spliceosome activation appears to be evolutionarily connected to the SF3B6-SF3B1$^{N\text{-}TP/SP}$-CDK11 axis, as in the absence of SF3B6 and CDK11 in various organisms such as *S. cerevisiae*, SMU1/RED is also missing (see our more detailed discussion of this point above).

8. Page 19, line 520: “HR 1–2” should be used instead of “HR 1–3”.

Done

9. In the Discussion (page 19, line 529), the statement should omit the term "phosphorylated," as SF3B1 is not phosphorylated in the pre-Bact-OTS complex, but rather in the pre-Bact and Bact complexes.

Done

Similarly, in the Extended Data Figure 6e T426, T434 and T436 are labeled by the same color irrespectively of their phosphorylation status at different steps of the spliceosome activation (Bact-OTS, Bact-1/2 and Bact) which is misleading. This needs to be corrected and clarified. It is also not clear why amino acids 67 (SF3B6) and 394 (SF3B1) are highlighted in the Extended Data Figure 6e, please explain.

We thank the reviewer for pointing out these errors. In the revised version we have changed the colors of T426, T434, and T436 in the pre-$B^{act\text{-}OTS}$ state. We have also deleted the labels for SF3B6 aa 67 from the figure to avoid any possible confusion. Amino acid 394 of SF3B1 is still labelled as we mention the SF3B6/$SF3B1^{394\text{-}430}$ module in the text, and therefore it is necessary to indicate where this amino acid is located.

We note that in this context we have also referred to a recent paper by Blazek and co-workers (reference 53) the revised version of the manuscript:

Consistent with our structural analysis, it has recently been shown that CDK11-mediated SF3B1 phosphorylation is functionally coupled to the SNIP1-dependent recruitment of the RES complex during B-to-B^{act} transition [53].

10a. Page 5, line 118 and elsewhere: The statement "Aberrant spliceosomes would impair CDK11 recruitment (and thus SF3B1 phosphorylation)..." contradicts the data presented in Data S1, where CDK11-CCNL-SAP30BP are clearly crosslinked to the spliceosome in the pre-Bact-OTS complex. This indicates that aberrant spliceosomes do not impair CDK11 recruitment; rather, the recruited kinase is inhibited by OTS964 in the pre-Bact-OTS complex.

"Aberrant spliceosomes would impair....": We would like to point out that the term "Aberrant spliceosomes" refers in our model to spliceosomes that have undergone aberrant remodeling events during early activation, which would prevent the docking of the N-terminal domain of SF3B1 at the PRP8-RT domain. In this sense, our pre-$B^{act\text{-}OTS}$ complex does not really represent an aberrantly remodeled intermediate assembly stage (our cryo-EM structure testifies too the native state of the remodeled early activated spliceosome) – and we show docking of $SF3B1^{N}$ to $PRP8^{RT}$ (Extended Data Fig. 6b,c); only owing to the inhibition of CDK11, pre-$B^{act\text{-}OTS}$ cannot progress further towards pre-$B^{act\text{-}1}$, and therefore it mimics an aberrant assembly state, allowing the binding of DHX15 to $SF3B1^{HEAT}$.

"..where CDK11-CCNL-SAP30BP are clearly crosslinked to the spliceosome ...":

While Supplementary Table 2 indeed shows some crosslinks of the CDK11-CCNL1/2-SAP30BP complex to pre-$B^{act\text{-}OTS}$, we would like to emphasize that they are minor. First of all, the amounts of the CDK11 kinase proteins that co-purify with our pre-$B^{act\text{-}OTS}$ complexes are very low (they do not appear among the 100 most abundant proteins). The crosslinks in Supplementary Table 2 were detected by carrying out the p-Link searches against the top 303 proteins. Yet, most of the crosslinks at FDR 1% are observed between the three protein subunits of the CDK11 complex. A few, very weak, crosslinks are observed at FDR 5% to spliceosomal proteins. However, several of the later proteins, such as hnRNP D, SYF1, TRA2B, PABP1 or ROA1, are not parts of our pre-$B^{act\text{-}OTS}$ cryo-EM structure. Thus, in our view, these crosslinks do not really allow conclusions about the binding site of the kinase complex at the pre-$B^{act\text{-}OTS}$ snapshot.

10b. In a similar vein, while SAP30BP may play a role in recruiting the CDK11 complex to certain spliceosome intermediates (as discussed on page 22, line 616), the mechanism proposed in reference 52 is difficult to reconcile with the current findings, since SAP30BP recruitment in that study depends on SF3B1 phosphorylation, which itself depends on CDK11 activity.

We have referred to the paper by Mayeda and coworkers (PMID: 38065098, ref. 47), primarily because they showed for the first time that SAP30BP can in principle interact with SPF45 via their ULM and UHM modules, respectively. Otherwise, the roles discussed for SPF45 and SAP30BP in the study by Mayeda and coworkers are significantly different from those in our study. For example, they propose that SPF45 interacts via its G patch with the poly-Py tract of very short introns at an early stage of spliceosome formation, thereby likely facilitating the deposition of an unknown factor X at the 3′SS of the pre-mRNA (or *vice versa*). In their study, SAP30BP

appears to play a chaperone role for SPF45, controlling the timely interaction of SPF45 with SF3B1. In our view, the role the authors discuss for the phosphorylation of SF3B1's N-terminus in the recruitment of SPF45 to SF3B1 remains enigmatic, as it would require phosphorylation of SF3B1N at an early spliceosome assembly state, for which there is no evidence.

In contrast, in our study, the proposed SPF45-SAP30BP interaction, would happen at a later stage of spliceosome assembly, shortly after activation, but prior to SF3B1 phosphorylation. Moreover, we would like to point out that in the pre-$B^{act\text{-}OTS}$ state, the poly-pyrimidine tract downstream of PM5-10 would not be available for interaction with SPF45 at this state, as it is occluded by the inner ring of the SF3B1-HEAT domain. Finally, it is very likely that in the study by Mayeda and coworkers, SPF45 and SAP30BP function as individual proteins, while in our study, they are part of the SPF45-SR140-CHERP complex and the CDK11 kinase heterotrimer, respectively; thus, no interaction of SPF45 with SF3B1 needs to be envisaged for the recruitment of SPF45.

Therefore, it was (and still is) tempting to suggest that upon initial docking of CDK11 kinase to $SF3B1^N$, SAP30BP may be tethered to SPF45 to facilitate efficient phosphorylation of the numerous TP and SP phosphorylation motifs. However, we agree that in the absence of any experimental evidence this specific interaction model is premature. Therefore, we would prefer to present this tethering model in the revised manuscript in more general terms:

While the mechanism of the recruitment of CDK11 to SF3B1 remains unclear, we propose that the tethering of the N-terminal domain of SF3B1 to $PRP8^{RT}$ in pre-$B^{act\text{-}OTS}$ may re-orient its TP/SP-rich central region, thereby presenting a high-affinity interaction site for the CDK11 kinase. It remains to be seen whether, upon initial docking to $SF3B1^N$, the CDK11 complex is further stabilized by additional interactions with one or more other spliceosomal proteins, thus facilitating efficient (and possibly processive) phosphorylation of the numerous TP/SP phosphorylation sites. For example, SAP30BP and SPF45 have been shown in principle to interact through their ULM and UHM modules, respectively (though in a spliceosomal context very different from ours)[47]. It is therefore tempting to suggest that the binding of CDK11 to the spliceosome could be stabilized by interactions between those two proteins. Alternatively, or additionally, cyclin L, which contains an RS-rich region, may also play a role in tethering CDK11 to the spliceosome. We anticipate that elucidating the structural basis of the targeting of CDK11 to SF3B1 will prove important for attaining a full understanding of the proposed kinase-dependent proofreading mechanism. (lines 690-708).

10c.In this context, it would also be important to discuss the role of SPF45, which has been shown in several studies to be recruited to unphosphorylated SF3B1 in earlier spliceosome intermediates and to function predominantly in the splicing of short introns or in alternative splicing regulation (PMID: 17589525, PMID: 34389706, PMID: 38065098). Once again, this raises the question of the extent to which the spliceosome structures described in this manuscript are specific to short introns due to the use of PM5-10 pre-mRNA, and to what extent the conclusions can be generalized, given that OTS964 blocks constitutive splicing in cells (PMID: 36104565, PMID: 38059508). These potential limitations should be discussed.

As outlined above, the role SPF45 appears to play in the splicing of very short pre-mRNAs (PMID: 3438970, PMID: 38065098), is different from possible roles that we envisage for SPF45 in the context of our studies.

…*or in alternative splicing regulation (PMID: 17589525, PMID: 34389706, PMID: 38065098).*

In the paper by Sattler, Valcarcel and coworkers (PMID: 17589525), it has been shown that overexpression of SPF45 in cells induces exon-6-skipping in the FAS receptor pre-mRNA, and that the alternative splicing regulation activity of SPF45 is dependent on the interaction of its UHM motif with a ULM motif of constitutive splicing factors such as SF1 and/or SF3B1. While the exact mechanism of how SPF45 may cause exon-6-skipping of FAS pre-mRNA remains an open question, it is evident that this regulation event will happen during early spliceosome formation at intron 5, before commitment-complex formation, and not after spliceosome activation, where the SPF45-SR140-CHERP complex comes into play in our study. It is interesting to note, though, that SPF45 regulates alternative splicing of exon 6, although the flanking introns 5 and 6, which are relevant for this regulation, are not members of the short intron type, studied by Mayeda and coworkers (PMID: 34389706, PMID: 38065098, and see above). Thus, SPF45 can in principle play various distinct roles in pre-mRNA splicing, and interact with the spliceosome at distinct stages of its assembly. However, as the molecular basis of those functions remains poorly understood, we do not think that it is helpful to discuss these roles in our manuscript in detail. We will, of course, refer to the papers discussed above in our revised manuscript as follows:

We note that our crosslink dataset also shows crosslinks between SPF45 and DHX15 (Supplementary Table 2), but not with $SF3B1^{HEAT}$. Whether SPF45 may recruit DHX15 to SF3B1 in the context of an aberrantly remodeled early activated spliceosome remains to be seen. As discussed below, SPF45 could also play a role in facilitating the productive interaction of CDK11 kinase with $pre\text{-}B^{act\text{-}OTS}$ stage spliceosomes. Previous studies have shown that SPF45 exerts additional functions in pre-mRNA splicing, such as promoting spliceosome formation on very short introns[46,47], or inducing exon 6 skipping in the FAS receptor pre-mRNA[48], roles in which SPF45 has to act at early stages of spliceosome formation, before the activation step. (lines 410-419)

Reviewer 3:

Zhang et al. present a structural and functional analysis of a novel early-activated spliceosome intermediate, termed pre-Bact-OTS complex, stalled by the CDK11 inhibitor OTS964. By integrating single-particle cryo-EM, crosslinking mass spectrometry, biochemical assays, and AlphaFold3 modeling, the authors elucidate the mechanism through which CDK11-mediated phosphorylation of SF3B1 regulates spliceosome activation and reveal how the helicase DHX15 may proofread this process. While post-translational modifications (PTMs) such as SF3B1 phosphorylation have been recognized as important regulatory events in splicing, the precise structural and functional consequences of this phosphorylation have remained unclear. This study demonstrates that phosphorylation of this core splicing factor triggers a critical structural rearrangement, thereby establishing a checkpoint for the proofreading of spliceosome assembly. Importantly, the work identifies the recruitment of DHX15 and its associated G-patch proteins to this intermediate, with SR140 serving as a key binding hub. Although DHX15 is known for its role in dismantling the ILS complex at the end of the splicing cycle and has recently been implicated in proofreading early assembly stages, this study significantly expands its functional repertoire, positioning DHX15 as a general proofreading factor active at multiple points in the splicing cycle.

Overall, this work provides crucial insights into how PTMs regulate the splicing cycle and how the spliceosome in higher eukaryotes may take advantage of such modifications as checkpoints to ensure fidelity. The implications of this research could extend beyond the splicing field, offering valuable perspectives also for other RNP biogenesis processes. Thus, I am happy to support publication of the manuscript in Nature Communications.

A few comments:

1. The Results section is generally dense and may be challenging for readers not deeply familiar with spliceosome structural biology. Given the broad readership of this journal, providing additional context and simplifying some of the detailed descriptions would greatly improve accessibility and broaden the impact of the work.

As requested also by the other referees, we have provided additional background information at several places in the revised manuscript (see also our response to points 1 and 2 of reviewer 1). Moreover, we have further streamlined the main text by moving some of the more detailed descriptions of the cryo-EM structure to the legends of the corresponding extended figures.

2. The discussion on the absence of this kinase-switch system in S. cerevisiae is interesting and highlights evolutionary complexity in humans/higher eukaryotes. It raises the question of whether this mechanism is conserved in other organisms such as S. pombe or C. elegans, which possess an SF3B6 counterpart. A brief bioinformatic analysis commenting on the conservation of SF3B6, the N-terminal region of SF3B1, and CDK11 across species would be a valuable addition.

Please see our response to a very similar request by reviewer 1 (point 14 above).

3. The mechanism of CDK11 complex recruitment to the spliceosome remains unclear. Specifically, why is CDK11 recruited specifically to this intermediate, and not to others where the SF3B1 N-terminus might also be accessible? Expanding the discussion on this point would be beneficial.

We agree with reviewer 3 that this is an important issue, which is still poorly understood. As the reviewer correctly states, the N-terminal domain of SF3B1 should be accessible in earlier stages of spliceosome assembly, such as the 17S U2 snRNP, A complex, pre-B complex and B complex, yet the $SF3B1^{N}$ domain is not phosphorylated. We were therefore excited to see that in our $pre\text{-}B^{act\text{-}OTS}$ complex, the N-terminal region of

$SF3B1^N$ is docked to the RT domain of PRP8, which positions the central, TP/SP rich domain of $SF3B1^N$ between $SF3B1^{HEAT}$ and $PRP8^{RT}$ (Extended Data Fig 6b). As the tethering of $SF3B1^N$ to the $PRP8^{RT}$ domain is exclusively observed at the pre-$B^{act\text{-}OTS}$ stage of spliceosome assembly, while in the B complex and later stages of spliceosome activation this region of PRP8 interacts in a mutually exclusive manner with other spliceosomal proteins (see Extended Data Fig. 6c), we propose that it facilitates the recruitment of the CDK11 kinase to $SF3B1^N$, and thus its efficient phosphorylation. For example, the tethering of the N-terminal domain of SF3B1 to $PRP8^{RT}$ in pre-$B^{act\text{-}OTS}$ may reorient its TP/SP rich central domain, thereby presenting a high-affinity binding site for the CDK11 kinase. At least, this would explain, why $SF3B1^N$ is phosphorylated first at the pre-$B^{act\text{-}OTS}$ stage, as it requires the significant movement of the U2 5' domain from its peripheral position in the B complex closer to the core of the spliceosome, that is to PRP8. Moreover, as SAP30BP, the stabilizing component of CDK11 (ref. 6), has been shown to interact in principle with SPF45 via their ULM and UHM motifs, respectively (ref. 47), we speculate that upon initial docking of CDK11 kinase to $SF3B1^N$, SAP30BP may be tethered to SPF45 to facilitate efficient, possibly processive, phosphorylation of the numerous TP and SP phosphorylation motifs. In the revised version, we discuss this point in the context of a potential facilitating role of the CDK11 kinase subunit SAP30BP at the end of the Discussion (lines 690-708; see also response to point 10b of reviewer 2)

4. The Results section includes a segment on the NTC/NTR complex and notes that IBC proteins are loosely associated. The implications of these observations are not discussed later. A brief comment on the potential significance of these associations in the Discussion would be helpful.

We have added the following sentences to the Discussion of the revised manuscript:

That is: Our pre-$B^{act\text{-}OTS}$ snapshot visualizes the handover of two outgoing proteins (SMU1/RED, which were recruited at the B complex stage), for new, incoming proteins – such as NTC and IBC proteins – that are positioned in pre-$B^{act\text{-}OTS}$ at intermediate spliceosomal docking sites (Fig. 4, Extended Data Fig. 4 and 5)). The provisional docking of NTC/IBC proteins will presumably enhance the kinetics of their final integration into the activated spliceosome – a principle that may be expected to apply to the exchange of many other proteins during the entire activation phase. (lines 511-518).
